# Repurposing Linezolid in Conjunction with Histone Deacetylase Inhibitor Access in the Realm of Glioblastoma Therapies

**DOI:** 10.1021/acs.jmedchem.4c02086

**Published:** 2025-01-21

**Authors:** I-Chung Chen, Hong-Yi Lin, Zheng-Yang Liu, Wei-Jie Cheng, Tzu-Yi Yeh, Wen-Bin Yang, Hoang Yen Tran, Mei-Jung Lai, Chung-Han Wang, Tzu-Yuan Kao, Chia-Yang Hung, Ya-Lin Huang, Ke-Chi Liou, Chien-Ming Hsieh, Tsung-I Hsu, Jing-Ping Liou

**Affiliations:** † School of Pharmacy, College of Pharmacy, Taipei Medical University, Taipei 110, Taiwan; ‡ Taipei Neuroscience Institute, New Taipei City 235, Taiwan; § Graduate Institute of Medical Sciences, College of Medicine, Taipei Medical University, Taipei 110, Taiwan; ∥ Ph.D. Program in Medical Neuroscience, College of Medical Science and Technology, Taipei Medical University and National Health Research Institutes, Taipei 110, Taiwan; ⊥ International Master Program in Medical Neuroscience, College of Medical Science and Technology, Taipei Medical University Taipei 110, Taiwan; # TMU Research Center of Neuroscience, Taipei Medical University, Taipei 110, Taiwan; ¶ Department of Pharmacology and Clinical Pharmacy, Faculty of Pharmacy, Can Tho University of Medicine and Pharmacy, Can Tho 902342, Vietnam; ∇ TMU Research Center for Drug Discovery, Taipei Medical University, Taipei 110, Taiwan; ○ Department of Immuno-Oncology, Beckman Research Institute, City of Hope, Duarte, California 91010, United States; ⧫ Ph.D. Program in Drug Discovery and Development Industry, College of Pharmacy, Taipei Medical University, Taipei 110, Taiwan; †† TMU Research Center of Cancer Translational Medicine, Taipei Medical University, Taipei 110, Taiwan; ‡‡ Department of Pharmaceutics, School of Pharmacy, University College, London WC1N 1AX, U.K.; ⬢ Taiwan Brain Disease Foundation, Taipei 100, Taiwan

## Abstract

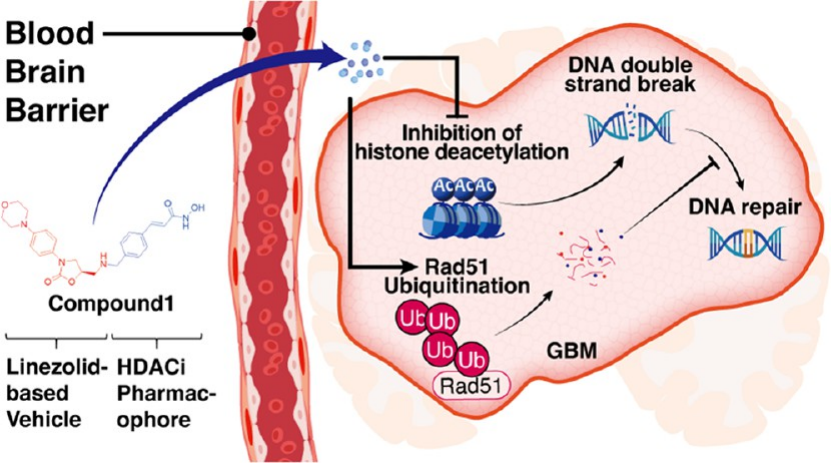

Since decades after temozolomide was approved, no effective drugs have been developed. Undoubtedly, blood–brain barrier (BBB) penetration is a severe issue that should be overcome in glioblastoma multiforme (GBM) drug development. In this research, we were inspired by linezolid through structural modification with several bioactive moieties to achieve the desired brain delivery. The results indicated that the histone deacetylase modification, referred to as compound **1**, demonstrated promising cytotoxic effects in various brain tumor cell lines. Further comprehensive mechanism studies indicated that compound **1** induced acetylation, leading to DNA double-strand breaks, and induced the ubiquitination of RAD51, disrupting the DNA repair process. Furthermore, compound **1** also exhibited dramatic improvement in the orthotopic GBM mouse model, demonstrating its efficacy and satisfying BBB penetration. Therefore, the reported compound **1**, provided with an independent therapeutic pathway, satisfying elongation in survival and tumor size reduction, and the ability to penetrate the BBB, was potent to achieve further development.

## Introduction

To date, the existing clinical technology still lacks a countering strategy against brain tumors, especially the most aggressive type, glioblastoma multiforme (GBM). Since temozolomide (TMZ), the first medical treatment for GBM, has been approved, the significant enhancement in overall survival (21.7 vs 12.7 months)^1^ conducted TMZ into first-line therapy; nevertheless, the hyper-methylation caused by TMZ often leads to a frustrated result. According to statistics, more than 50% of GBM patients generated TMZ resistance after treatment; this fact may be due to the overexpression of O6-methylguanine-DNA alkyl transferase (MGMT) and/or alkylpurine-DNA-*N*-glycosylase (APNG), activation of base excision repair, or DNA mismatch repair (MMR) silencing.^2^ In the past decades, researchers have dedicated themselves to seeking solutions for recurrent GBM. Indeed, several strategies, including bevacizumab, nitrogen mustards, and tumor-treating fields, have been approved. However, these therapies remained with some crucial defects, which are urgent to be solved, including no significance in overall survival,^3^ systemic toxicity,^3^ or the limitation of lesion location.^4^ Therefore, a novel therapeutic system should be developed for recurrent GBM.

The blood–brain barrier (BBB), the gatekeeper between the brain and the circulation system, resisted nearly 100% of small-molecule drugs penetrating through blood vessels into the brain,^5^ which acquired an enormous obstacle in GBM drug development. Recently, several tumor-aiming strategies have proved positive feedback in various solid tumors, such as a heptapeptide cyanine dye-mediated drug delivery system^6^ or numerous drug–drug conjugates.^7^ However, all of the successes were ruled out by GBM; hence, a more efficacious brain delivery system must be urgently developed. In this research, the established delivery system was inspired by the commercially available drug, linezolid, a synthetic antibiotic that has been used as a late-line therapy for infection from multiresistance Gram-positive bacteria.^6^ Clinically, linezolid has been widely applied in the treatment of central nervous system (CNS) infections, which demonstrated the promising penetration against the barrier between blood and the cerebrospinal fluid (CSF),^7,8^ especially in meningitis patients; some can even reach up to more than 70% penetration.^9^ On the other hand, by targeting the 50S ribosome in bacteria, the acting mechanism of linezolid was independent of affecting any of the homeostatic enzymes in *Homo sapiens*. Hence, the further repurposing of linezolid was expected to retain lipophilic barrier penetration ability with more predictable pharmacokinetic properties and acceptable safety profile.^10^

To investigate the penetration property of the linezolid carriers, several bioactive moieties were selected to achieve the desired modification. Recently, epigenetic dysregulation has been widely accepted as the initiation of numerous malignancies.^11,12^ Histone modification is the one which is crucial in altering not only cell cycle modulation, differentiation, and apoptosis but also tumor growth, progression, and drug resistance,^13^ which provides a potent target for GBM drug development. Study has revealed that the overexpression of class I histone deacetylases (HDACs) might be the major cause of TMZ resistance. The hyper-methylation caused by TMZ may have triggered the corresponding self-repair process, *O*6-methylguanine DNA damage repair, which enhanced the activation of RAD18, and class I HDACs were found to be the catalyst of RAD18 initiation.^14^ Besides, another DNA damage repair gene, RAD51, also upregulated in the clinical–pathological study of GBM patients and highly correlated with low overall survival.^15^ Current therapies for GBM were mainly targeting DNA double-strand breaking, including TMZ and radiation therapy, which indicated that the DNA repair process might be a key factor in determining the efficacy of corresponding therapies. Homologous recombination (HR) is one of the major pathways in repairing broken DNA double strands, during the process, and RAD51 facilitated the exchange between broken and unbroken DNA strand templates to repair the double-stand break.^16^ As the importance of RAD51 has been highlighted, several studies reported that RAD51 can be downregulated by HDAC inhibitors through silencing miR-182^17^^,^.^18^ Above all, the modulating effect of DNA repair by HDAC inhibition was expected to abolish the TMZ resistance. However, the identified zinc-binding motif in various HDAC inhibitors created a hydrophilic condition, which is unbenefited for the hydrophobic BBB penetration.^19^ Hence, the repurposing of linezolid-conjugated HDAC inhibitors was anticipated to achieve a high brain concentration.

**Figure 1:**
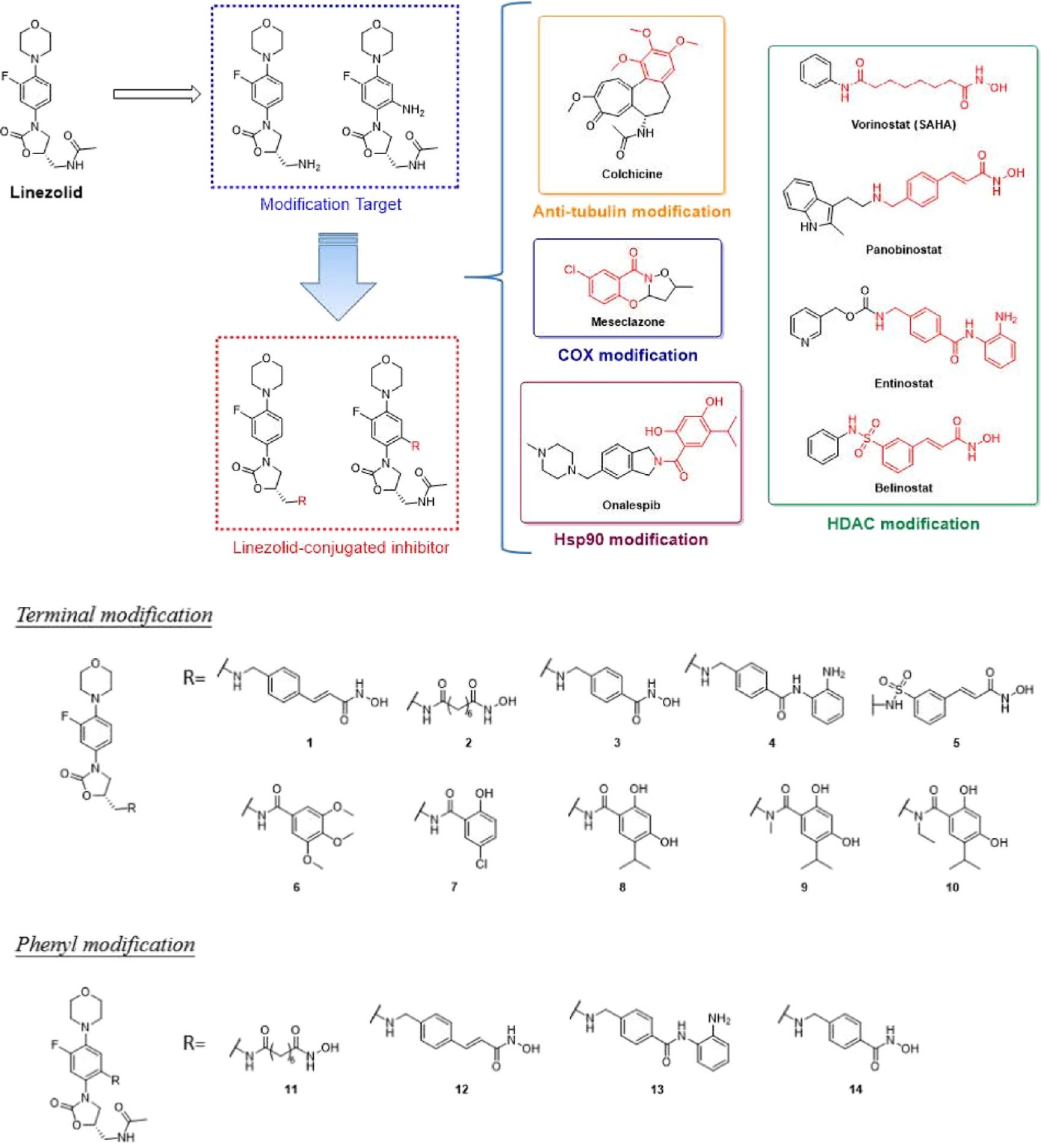
Rationale design of the linezolid-conjugated inhibitors and the synthesized compounds.

Beyond HDAC, heat shock protein 90 (Hsp90), a notable chaperone involved in protein folding, enzyme assembly, and ligand binding, was considered another potent therapeutic target. Based on our research, the inhibition of Hsp90 not only provides cell stress to induce apoptosis, but the tumor microenvironment modulation ability possessed a dramatic synergistic effect with other chemotherapy agents.^20^ Although the relationship between HSP90 inhibition and TMZ resistance was still unclear, HSP90 remains a promising target for developing novel GBM therapeutic strategies. Excluding direct modification on DNA, antitubulin agents have received promising feedback in various cancers, especially the two notable taxeme drugs, docetaxel and paclitaxel,^21^ but the low BBB penetration limited their application in brain tumors.^22^ Therefore, we aimed to fuse the pharmacophore, the 2,3,4-trimethoxy phenyl moiety, with linezolid. According to recent research, neuroinflammation facilitated drug resistance and progression in GBM,^23^ and the major cause of neuroinflammation was the enhancement of arachidonate metabolism to prostaglandins.^24^ Thus, we also considered the installation of 5-chlorosalicylic acid for the cyclooxygenase inhibition.^25^ In summary, the structural design was based on the structure of linezolid with the modification on either terminal amide or installed an amino group on the phenyl moiety to achieve our designing compound (Figure 1). Besides seeking for the potent target to attain a promising antitumor effect, BBB permeability was an inescapable obstacle in GBM drug development; therefore, based on the inherent lipophilic barrier penetrable ability of linezolid, the designing compounds were evaluated through two dimensions, the predicted logarithm of partition coefficient (log *P*) and topological polar surface area (tPSA). According to the reported analytical results of approved drugs, lower tPSA, approximately lower than 90 Å^2^, was beneficial for the BBB penetration;^26^ however, the currently approved GBM drug, TMZ, showed 103.72 Å^2^ in tPSA calculation, which indicated that tPSA is not the only parameter that effected BBB penetration; hence, log *P* was taken into consideration. Latest research has revealed that BBB rejected most of the pharmaceuticals, except for the hydrophilic compound with lower than 150 Da molecule weight and the hydrophobic compound lower than 400–600 Da.^27^ These two facts provided supportive evidence for both TMZ and linezolid brain transcytosis; therefore, in our designing strategy, the predicted log *P* was used for evaluating appreciative lipophilicity with the lower tPSA as possible (Supporting Information I).

## Results

### Chemistry

**Scheme 1:**
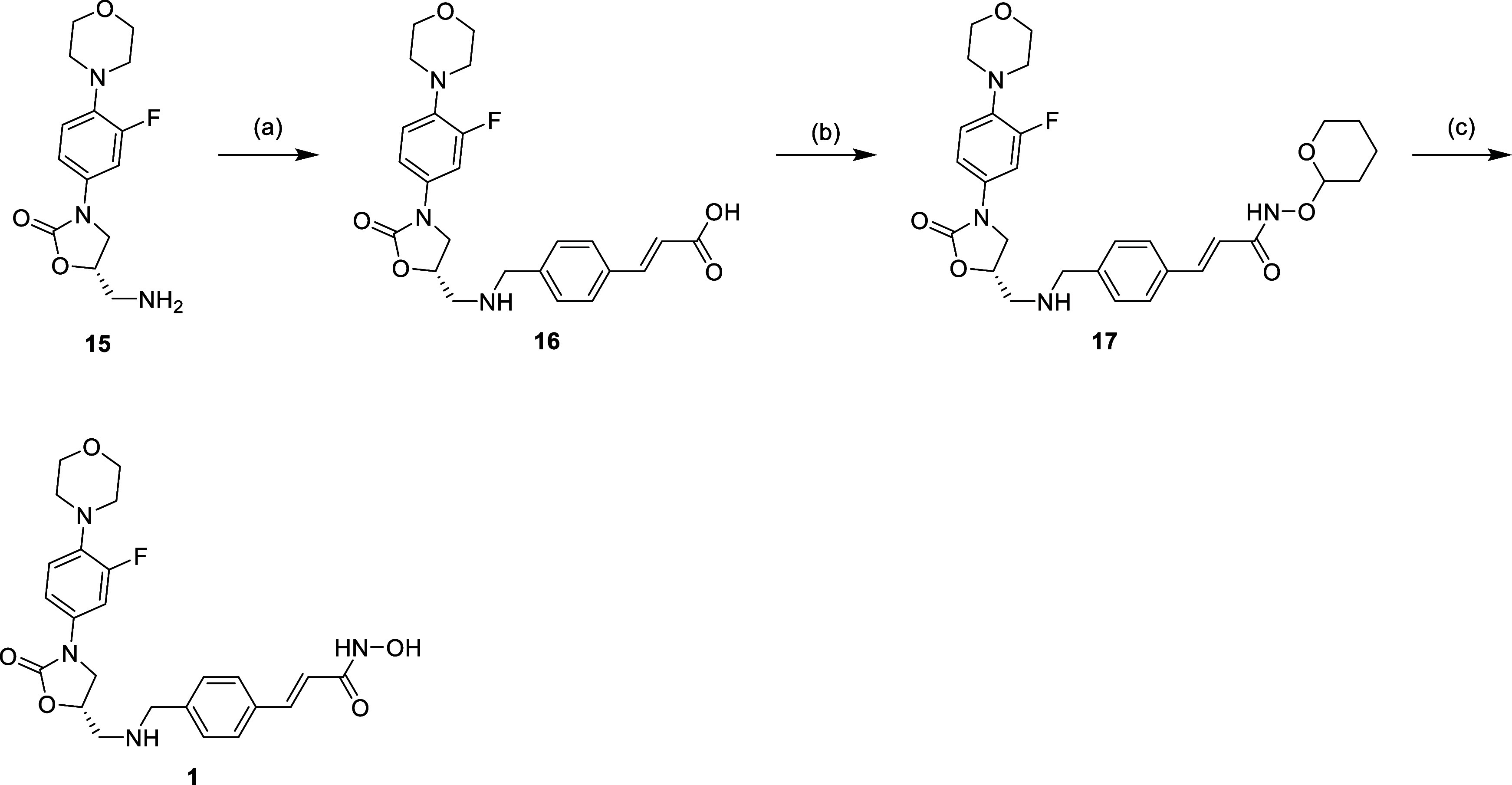
Reagents and Conditions: (a) (1) 4-Formyl Benzoic Acid, MeOH, Reflux, 16 h; (2) NaBH_4_, 0 °C to r.t., 2 h; (b) NH_2_OTHP, EDCi, HOBt, NMM, DMF, r.t., 16 h; and (c) 5% TFA_(MeOH)_, r.t., 16 h

**Scheme 2:**
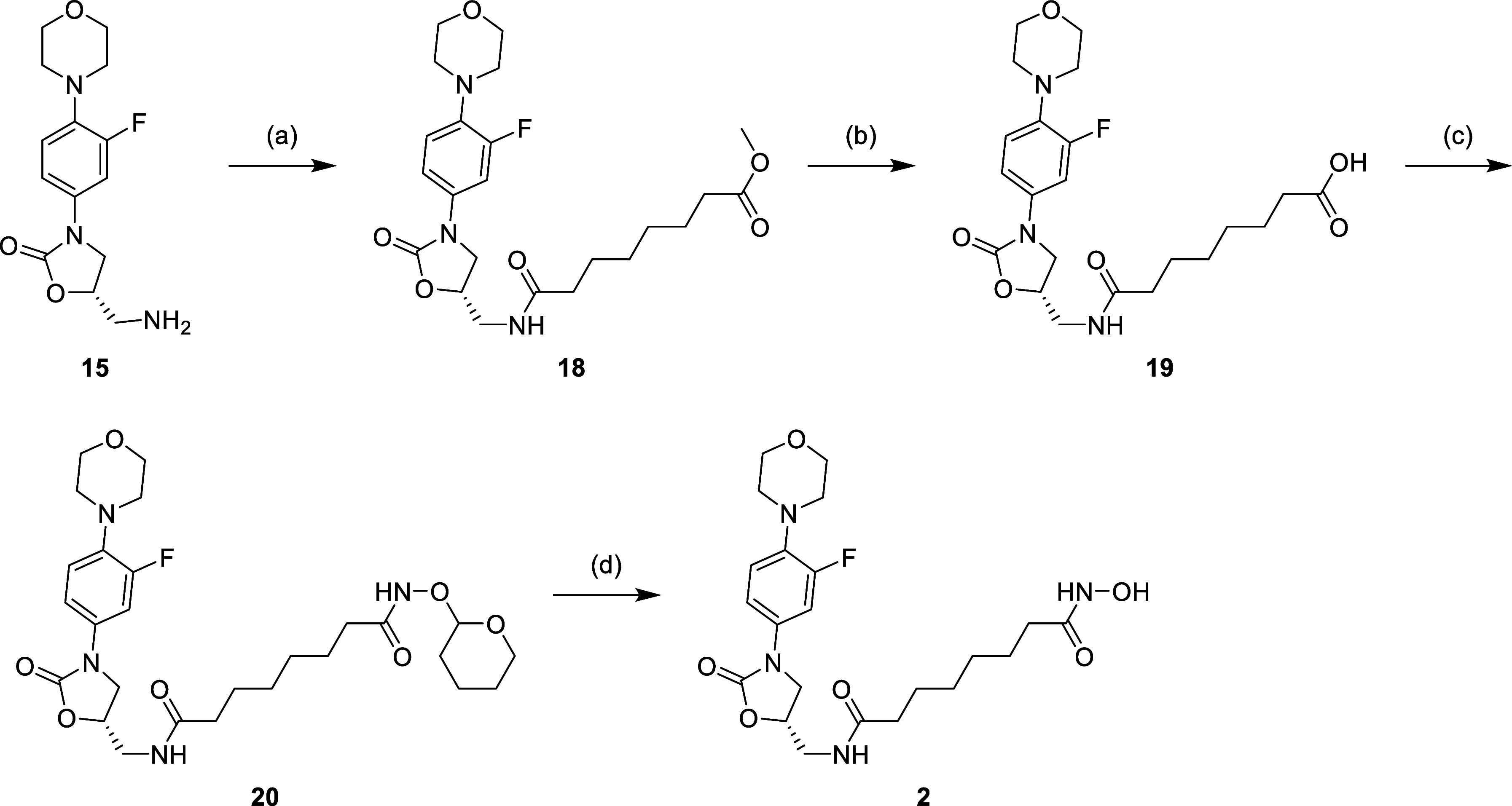
Reagents and Conditions: (a) (1) Monomethyl Suberate, Oxalyl Chloride, DCM, 0 °C to r.t., 2 h; (2) Pyridine, DCM, 0 °C to r.t., 16 h; (b) 1N LiOH(aq), Dioxane, r.t., 16 h; (c) NH_2_OTHP, EDCi, HOBt, NMM, DMF, r.t., 16 h; and (d) 5% TFA_(MeOH)_, r.t., 16 h

**Scheme 3:**
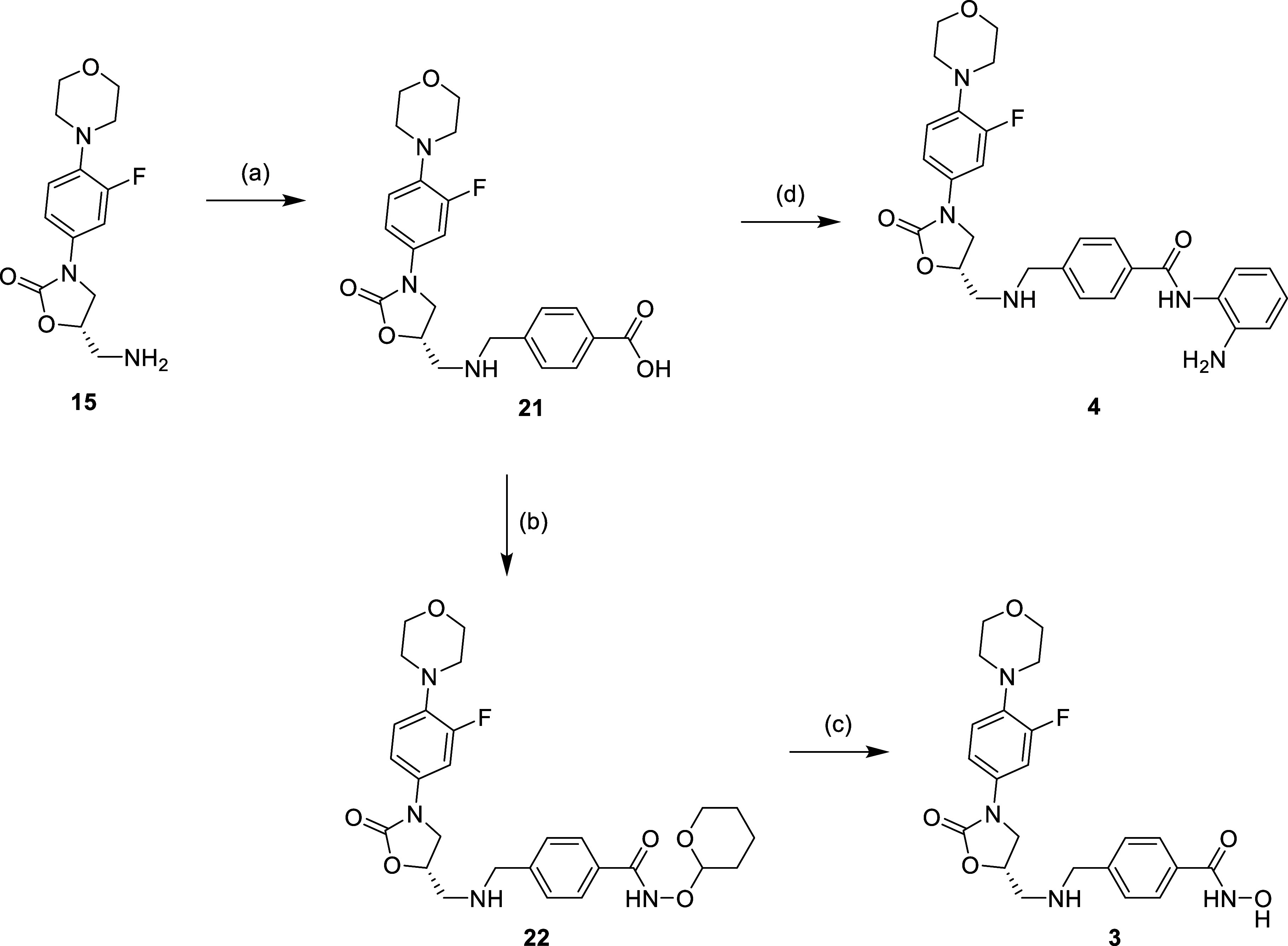
Reagents and Conditions: (a) (1) 4-Formyl Benzoic Acid, MeOH, Reflux, 16 h (2) NaBH_4_, 0 °C to r.t., 2 h; (b) NH_2_OTHP, EDCi, HOBt, NMM, DMF, r.t., 16 h; (c) 5% TFA_(MeOH)_, r.t., 16 h; and (d) *o*-phenylenediamine, EDCi, HOBt, NMM, DMF, r.t., 16 h

**Scheme 4:**
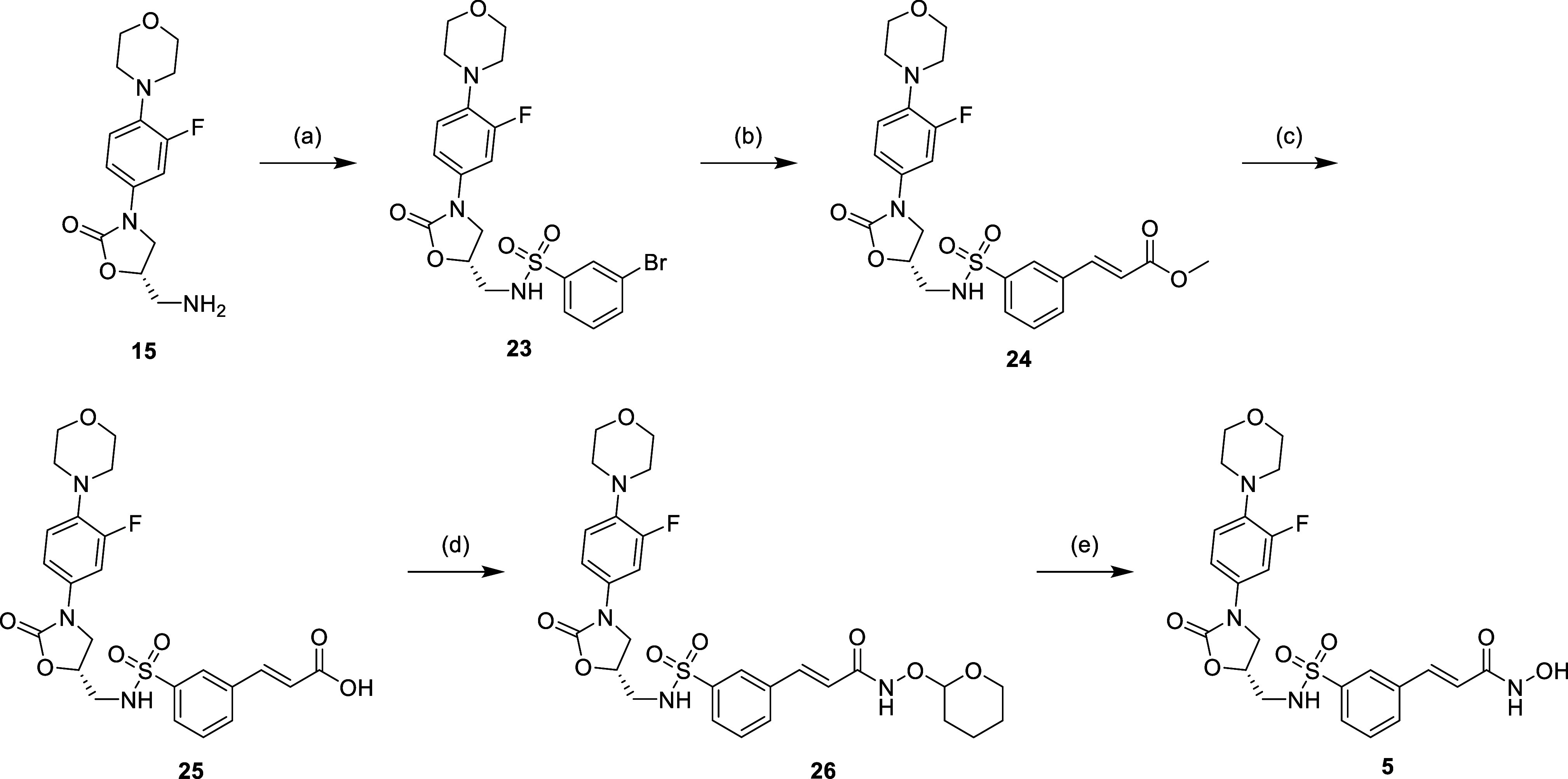
Reagents and Conditions: (a) 3-Bromobenzenesulfonyl Chloride, Pyridine, DCM, 0 °C to r.t., 1 h; (b) Methyl Acrylate, Pd(OAc)_2_, PPh_3_, NaHCO_3_, Et_3_N, DMF, 120 °C, 16 h; (c) 1N LiOH(aq), Dioxane, r.t., 16 h; (d) NH_2_OTHP, EDCi, HOBt, NMM, DMF, r.t., 16 h; and (e) 5% TFA_(MeOH)_, r.t., 16 h

**Scheme 5:**
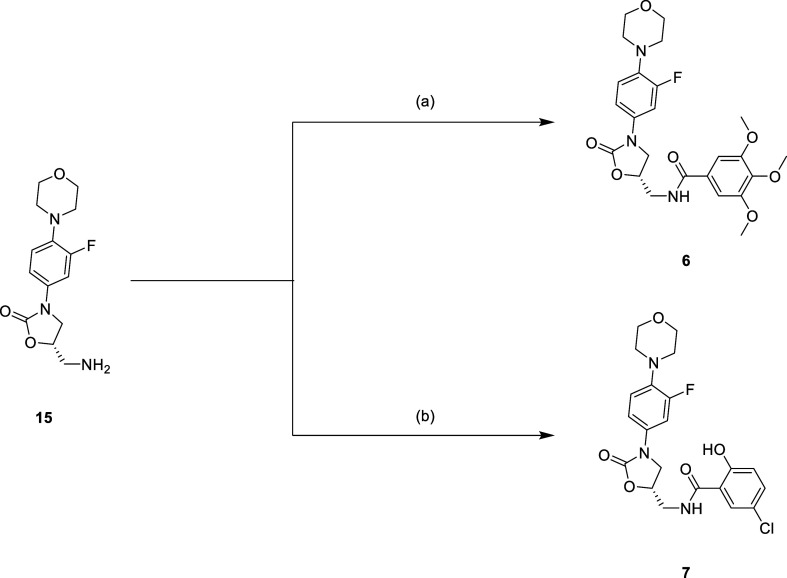
Reagents and Conditions: (a) 3,4,5-Trimethoxybenzoyl Chloride, Pyridine, DCM, 0 °C to r.t., 16 h; (b) 5-Chlorosalicylic Acid, EDCi, HOBt, NMM, DMF, r.t, 16 h

**Scheme 6:**
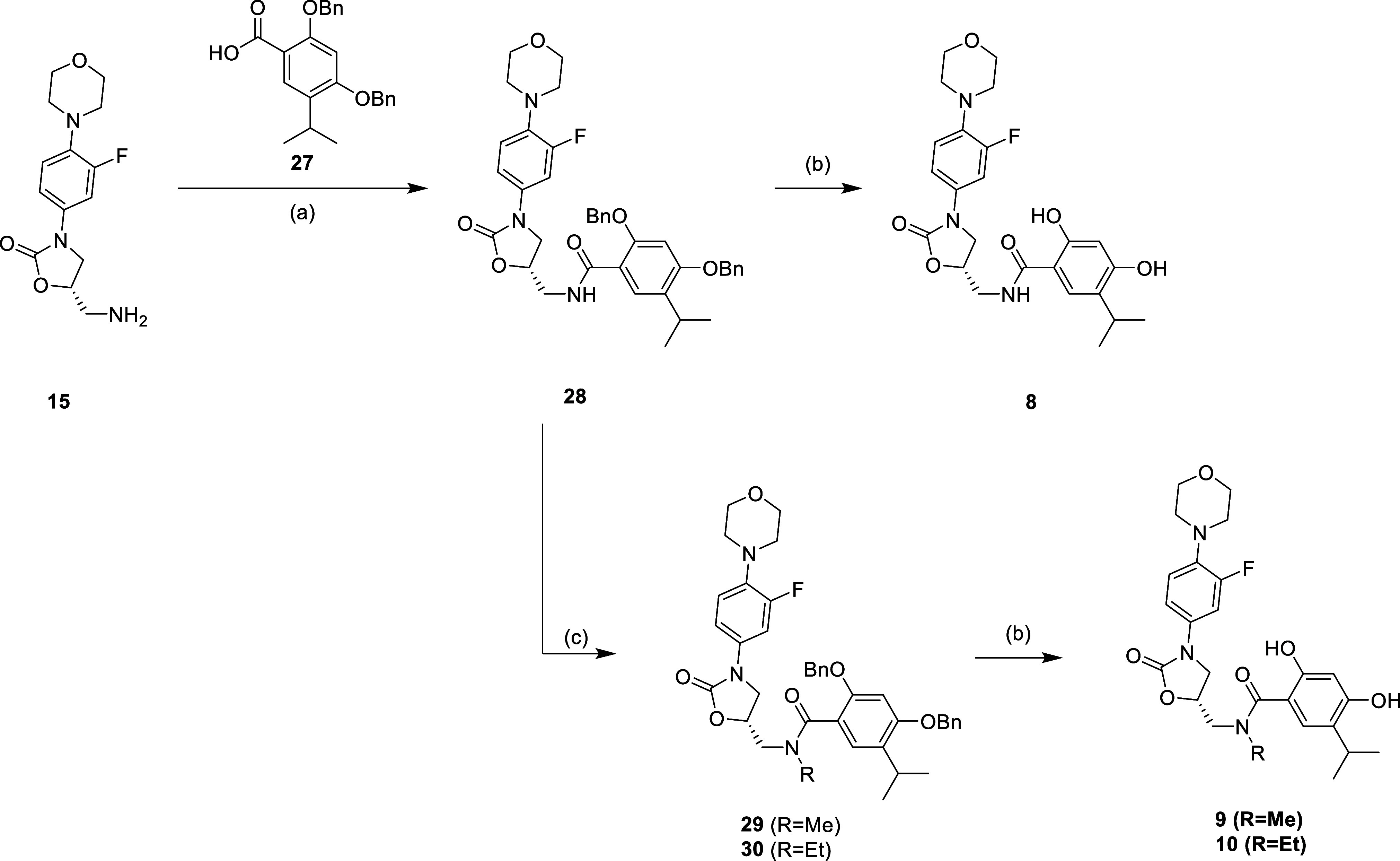
Reagents and Conditions: (a) EDCi, HOBt, NMM, DMF, r.t., 16 h; (b) H_2_, Pd/C, MeOH, r.t., 16 h; and (c) MeI or EtI, NaH, DMF, 0 °C to r.t., 8 h

Synthesis of compound **1** is illustrated in Scheme 1, starting with the linezolid-related amine (**15**) followed by the reductive amination with 4-formylcinnamic acid to afford the carboxylic acid (**16**). The carboxylic acid moiety was then subjected to amide coupling reaction with TPH-protected hydroxyl-amine to get compound **17**. Last, the protection group was removed under acidic conditions to afford desired compound **1**. Schemes 2–4 show the synthesis of other linezolid-based HDAC inhibitors; the synthetic routes only differed between the installation of the linker. For compound **2**, linezolid-related amine (**15**) was subjected to substitution with the corresponding acyl chloride of monomethyl suberate, which was then hydrolyzed to get carboxylic compound **19**, followed by the identical procedure for the construction of hydroxamic acid as shown in Scheme 2. For compounds **3** and **4**, the synthesis process was in the same manner as the synthesis of compound **1**, while *o*-phenylenediamine was used in the synthesis of compound **4**, instead of THP-protected hydroxylamine (Scheme 3). The synthesis of compound **5** started with S_N_2__ with 3-bromosulfonyl bromide, followed by the Heck coupling reaction with methyl acrylate to afford compound **24**. The further synthesis steps for the construction of hydroxamate were similar to those described previously (Scheme 4). For the variation of other linezolid-based enzyme inhibitors, was simply synthesized with the amide coupling reaction to afford compounds **6** and **7** (Scheme 5). The HSP90 inhibitors were synthesized with the corresponding carboxylic acid **27**, with or without the substitution on the amide moiety, followed by the removal of the benzyl protection group to afford compounds **8**, **9**, and **10** (Scheme 6).

**Scheme 7:**
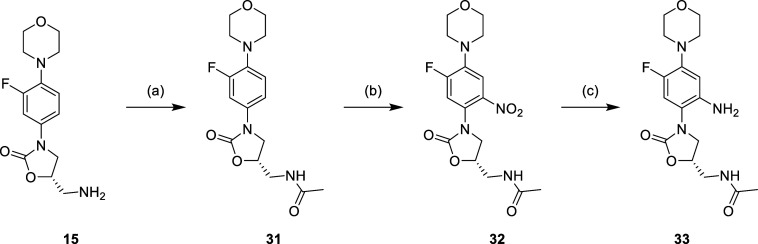
Reagents and Conditions: (a) Ac_2_O, PhMe, Reflux, 16 h; (b) HNO_3_, H_2_SO_4_, 0 °C to r.t., 1 h; and (c) H_2_, Pd/C, DCM, r.t., 16h

**Scheme 8:**
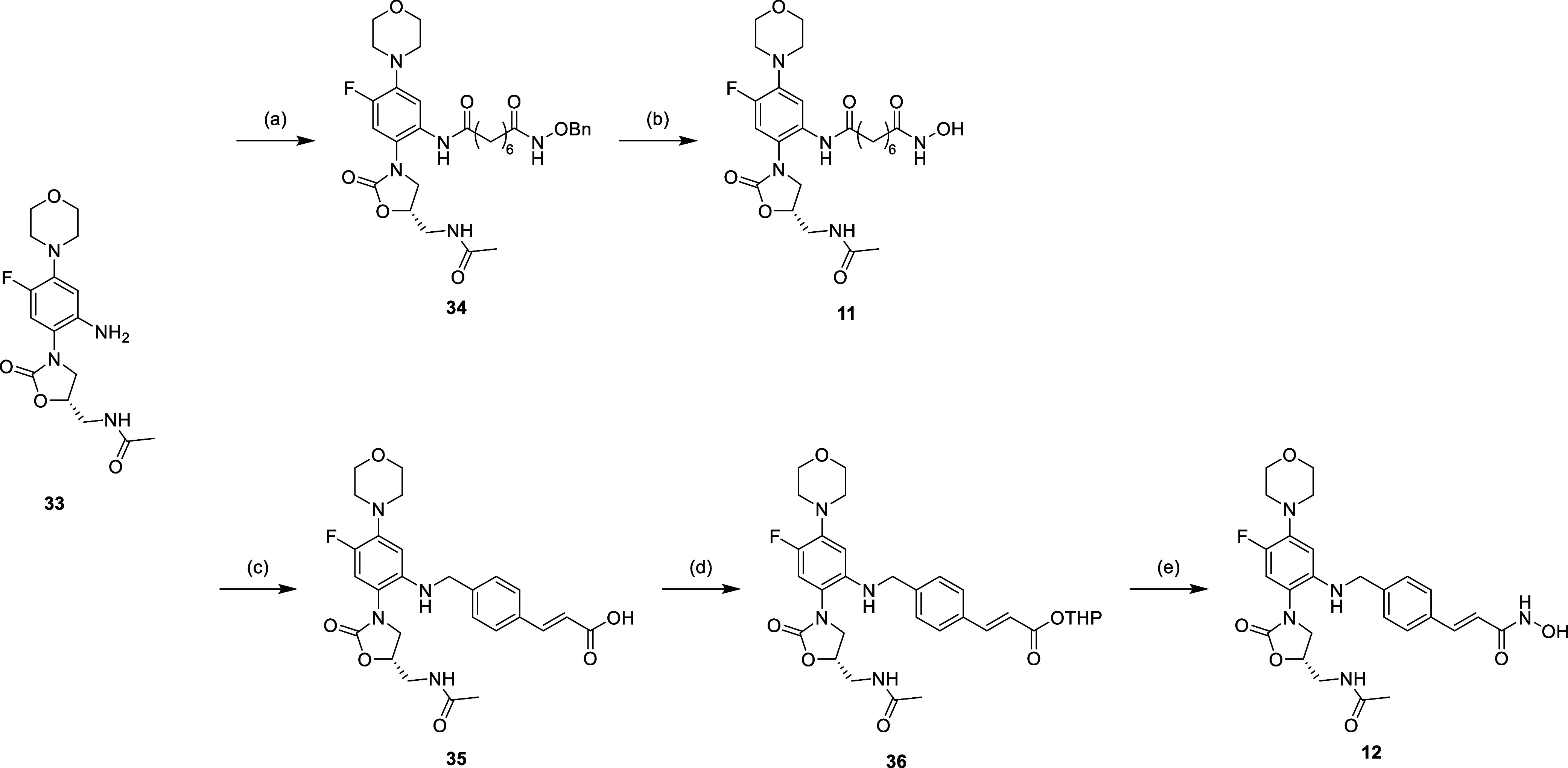
Reagents and Conditions: (a) Corresponding Carboxylic Acid, EDCi, HOBt, NMM, DMF, r.t., 16 h; (b) H_2_, Pd/C, MeOH, r.t., 16 h; (c) (1) 4-Formyl Cinnamic Acid, MeOH, Reflux, 16 h; (2) NaBH_4_, 0 °C to r.t., 2 h; (d) NH_2_OTHP, EDCi, HOBt, NMM, DMF, r.t., 16 h; and (e) 5% TFA_(MeOH)_, r.t., 16 h

**Scheme 9:**
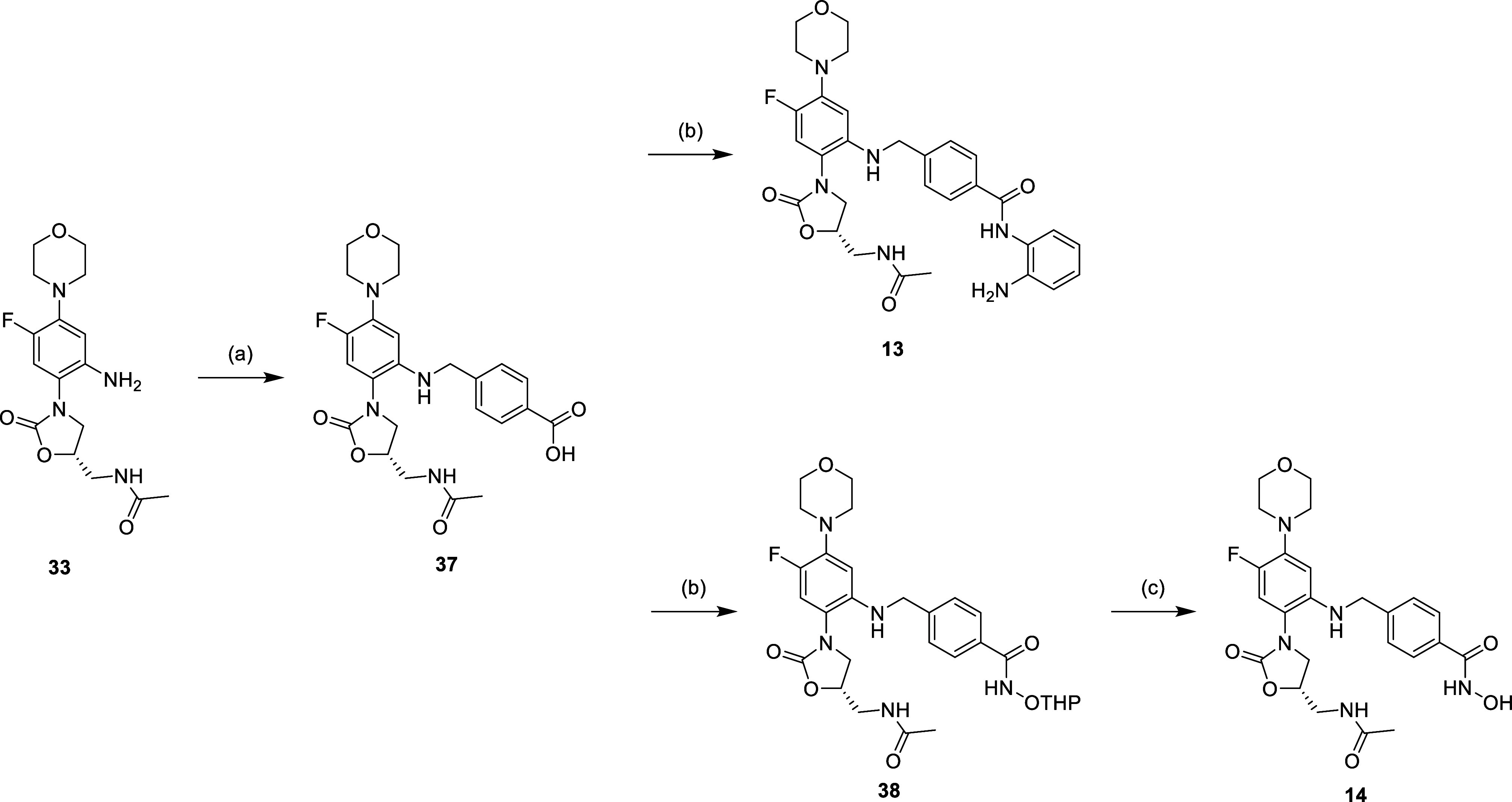
Reagents and Conditions: (a) (1) 4-Formyl Benzoic Acid, MeOH, Reflux, 16 h (2) NaBH_4_, 0 °C to r.t., 2 h; (b) *o*-Phenylenediamine, EDCi, HOBt, NMM, DMF, r.t., 16 h; (c) NH_2_OTHP, EDCi, HOBt, NMM, DMF, r.t., 16 h; and (d) 5% TFA_(MeOH)_, r.t., 16 h

The phenyl modification is shown in Schemes 7–9, starting with the linezolid-related amine (**15**) with the acetylation of the amino group to afford linezolid (**15**), then nitration though nitric acid, followed by the reduction of the nitro group to get aniline **33** (Scheme 7). For the further synthetic route, compound **33** was used as the starting material, which was then subjected to similar synthetic strategies as shown in Schemes 1–3 (Schemes 8 and 9). The results of nuclear magnetic resonance spectroscopy verification and HPLC purity analysis in this experiment are shown in Supporting Information II and III.

### Cytotoxicity of Linezolid-Conjugated Compound **1** against TMZ-Resistant GBM

**Figure 2:**
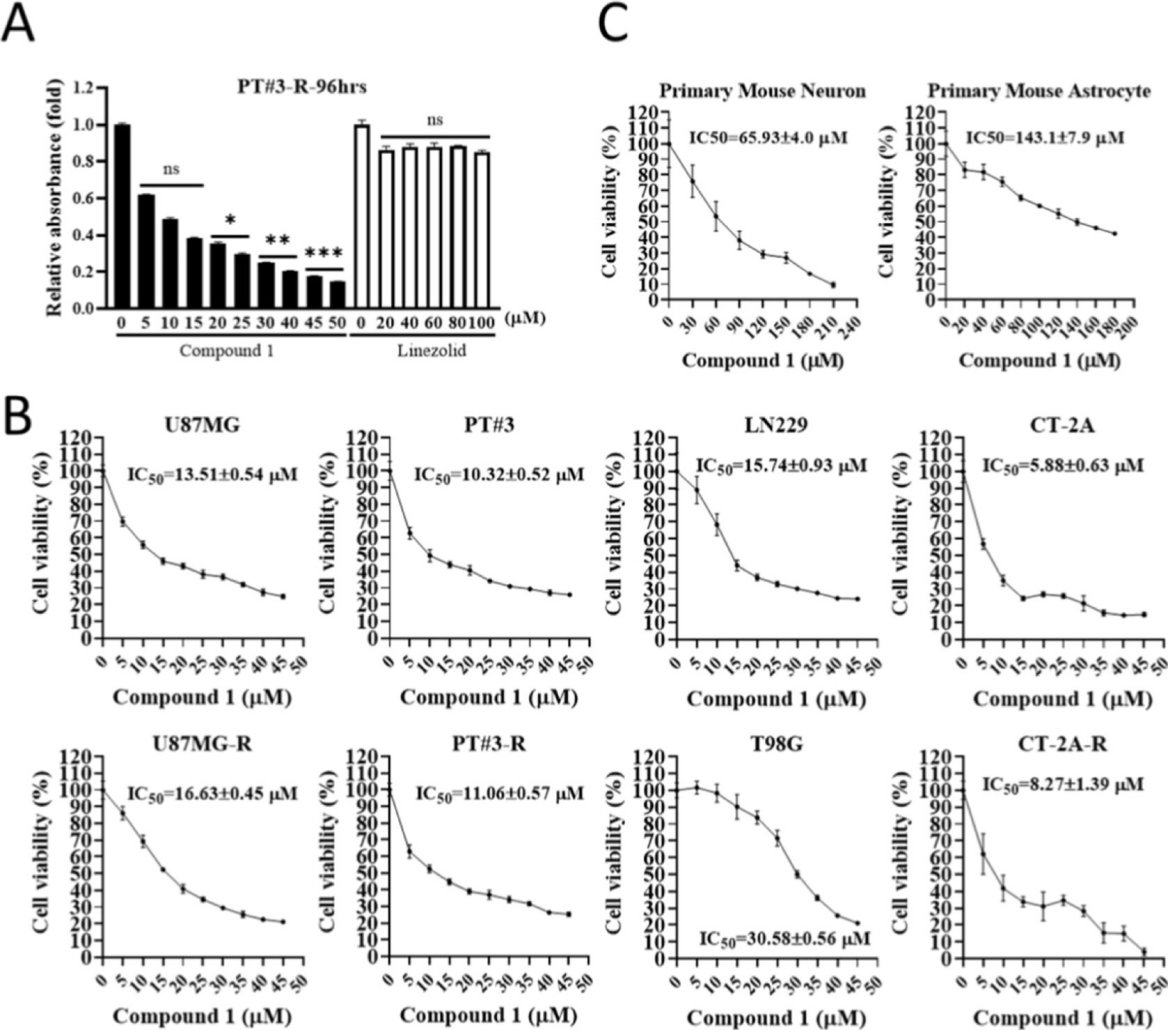
Inhibition of GBM and TMZ-resistant GBM growth by compound **1**. (A) Comparison of cytotoxic efficiency between compound **1** and linezolid using the MTT assay. (B) Evaluation of IC_50_ values for mouse astrocytoma, different GBM, TMZ-resistant variants, as well as (C) primary culture astrocytes and neurons using the CCK8 assay.

We utilized linezolid as the structural foundation to confirm the cytotoxicity of linezolid-conjugated functional domains against glioblastoma (GBM). We combined it with the binding structures of anti-histone deacetylases (HDACs) (denoted as compounds **1–5** and **11–14**), antitubulin (compound **6**), anticyclooxygenase (COX) (compound **7**), and anti-heat shock protein 90 (Hsp90) (compounds **8–10**). These constructs were then administered to TMZ-resistant GBM cell lines A172R and PT#3R at concentrations ranging from 0 to 80 μM over 72 h. The cytotoxic effects of these compounds were assessed using the MTT assay (Table 1 and Supporting Information IV). The results indicated that compounds **1**, **4**, and **9** exhibited the capacity to induce cytotoxicity in TMZ-resistant GBM, and the phenyl modification (compounds **11–14**) displayed no significant cytotoxicity. Given the remarkable cytotoxicity of compound **1** on both A172-R and PT#3-R TMZ-resistant cells, this study subsequently focused on compound **1** for further in-depth investigation and mechanistic exploration. To validate that the cytotoxic effect of compound **1** on GBM is not attributed to linezolid alone, we separately treated the TMZ-resistant GBM cell line PT#3-R with compound **1** and linezolid. The results revealed that linezolid, at concentrations ranging from 80 to 100 μM, did not induce cytotoxicity in resistant GBM cells. In contrast, compound **1**, at concentrations of 20 μM and above, exhibited a significant cytotoxic effect on resistant GBM cells, with cytotoxicity increasing proportionally with compound **1** concentration. This confirms that the cytotoxicity of compound **1** against GBM is primarily attributable to the inhibitory structure of linezolid-conjugated HDAC (Figure 2A). Furthermore, we compared the cytotoxic effects of compound **1** on various wild-type brain tumor cell lines, including U87MG, PT#3, LN229 GBM cells, and CT-2A mouse astrocyte cell line as well as TMZ-resistant variants cell lines, including U87MG-R, PT#3-R, CT-2A-R, and T98G (MGMT-positive). Compound **1** exhibited outstanding cytotoxicity, and the IC_50_ values remained consistent regardless of TMZ resistance (Figure 2B). Additionally, we further confirmed the efficacy of compound 1 in GBM treatment. While the induced IC_50_ in primary culture astrocytes was 143.1 ± 7.9 μM and in primary culture neurons was 65.93 ± 4.0 μM, it displayed inhibitory effects on proliferation and induced cell death in GBM, TMZ-resistant GBM, and patient-derived TMZ-resistant GBM cells, with IC_50_ values ranging from 10.32 to 30.58. Notably, the IC_50_ value for primary mouse astrocytes was higher, indicating a more excellent selectivity of compound **1** against GBM cells (Figure 2C).

**Table 1:** IC_50_ of Compounds **1–10** on A172R and PT#3R TMZ-Resistant GBM Cell Line Were Assessed Using the MTT Assay

	IC_50_ (μM)	
Cell line	A172-R	PT#3-R
compound **1**	17.41 ± 1.21	12.12 ± 1.48
compound **2**	N/A^a^	164.3 ± 41.5
compound **3**	N/A^a^	113.8 ± 20.79
compound **4**	18.98 ± 2.53	41.41 ± 5.01
compound **5**	N/A^a^	123.5 ± 17.5
compound **6**	N/A^a^	N/A^a^
compound **7**	224 ± 80.3	52.11 ± 6.26
compound **8**	65.25 ± 11.16	57.96 ± 6.23
compound **9**	35.62 ± 4.64	15.26 ± 2.10
compound **10**	80.56 ± 9.99	48.76 ± 4.77

a N/A indicated that the tested compound could not fit into the IC_50_ curve.

### Compound **1** Is a Targeted Inhibitor of Class I HDACs with Implications for Resistant GBM Treatment

**Figure 3:**
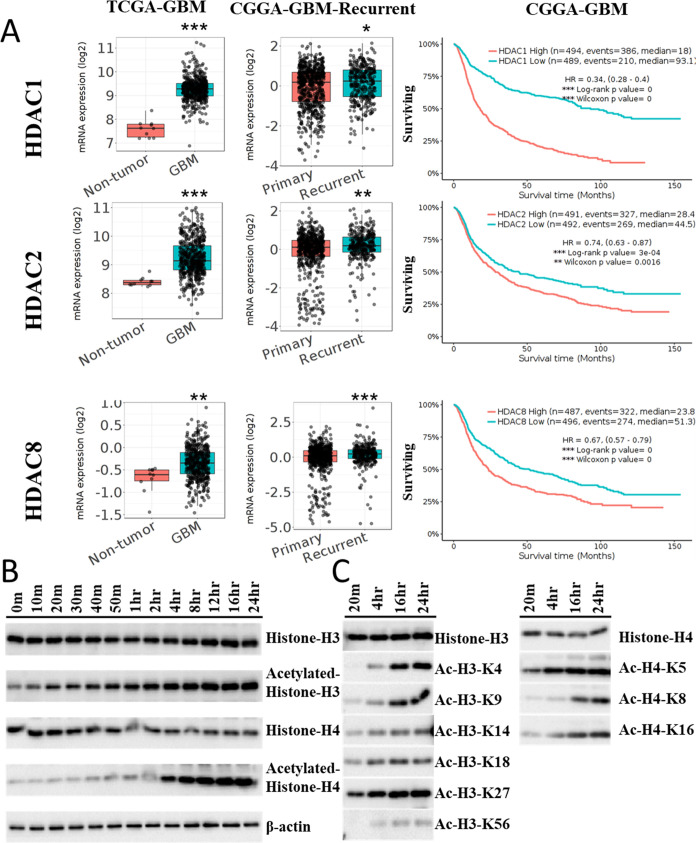
Compound **1** induces acetylation of histones H3 and H4. (A) Analysis of RNA expression levels of class I HDACs in nontumor, GBM, and recurrent GBM, along with Kaplan–Meier survival curves, based on TCGA and CGGA data. (B) Western blot analysis reveals a significant induction of acetylation in histones H3 and H4 within 1 h of treating U87MGR GBM cells with 10 μM compound **1**. (C) Analysis of acetylation patterns at different sites on histones H3 and H4.

To assess the inhibitory potential of compound **1** on hydroxydeacetylases (HDACs), we first identified its target. Enzyme inhibition assays unequivocally revealed that compound **1** effectively inhibits class I HDACs, specifically targeting HDACs 1, 2, and 8 (Table 2). Furthermore, a comprehensive analysis of The Cancer Genome Atlas Glioblastoma Multiforme (TCGA-GBM) and Chinese Glioma Genome Atlas (CGGA-GBM) databases unveiled notably elevated mRNA expression levels of class I HDACs within GBM compared to the normal brain tissue. Intriguingly, as primary GBM progresses to the recurrent stage, the abundance of class I HDACs further escalates. Moreover, the abnormally heightened expression of class I HDACs strongly correlates with unfavorable survival outcomes in GBM patients (Figure 3A). These collective findings underscore the pivotal role of class I HDACs in fueling GBM growth and fostering resistance to TMZ. To validate the inhibitory effects of compound **1** on class I HDACs and the resulting acetylation changes in GBM cells, we conducted an analysis of histone H3 and H4 acetylation levels after treatment with compound **1**. The results unveiled substantial acetylation modifications at distinct histone H3 and H4 sites following the compound **1** treatment (Figure 3B,C).

**Table 2:** IC_50_ of Compounds **1–5** on HDAC Activity

	IC_50_ (μM)^a^			
compound	HDAC1	HDAC2	HDAC6	HDAC8
1	1.09	2.81	0.106	0.447
2	3.37	6.43	0.304	1.11
3	>10	>10	0.061	0.938
4	0.979	2.02	N/A^b^	N/A^b^
5	0.735	1.66	0.095	0.803
trichostatin A	0.006	0.021	0.002	0.923

a These assays were conducted by the Reaction Biology Corporation, Malvern, Pennsylvania. All compounds were dissolved in DMSO and tested in a 10-dose IC_50_ mode with 3-fold serial dilution starting at 10 μM.

b N/A indicated that the tested compound could not fit into the IC_50_ curve.

### Compound **1** Disrupts GBM Cell Growth via DNA Function Inhibition

**Figure 4:**
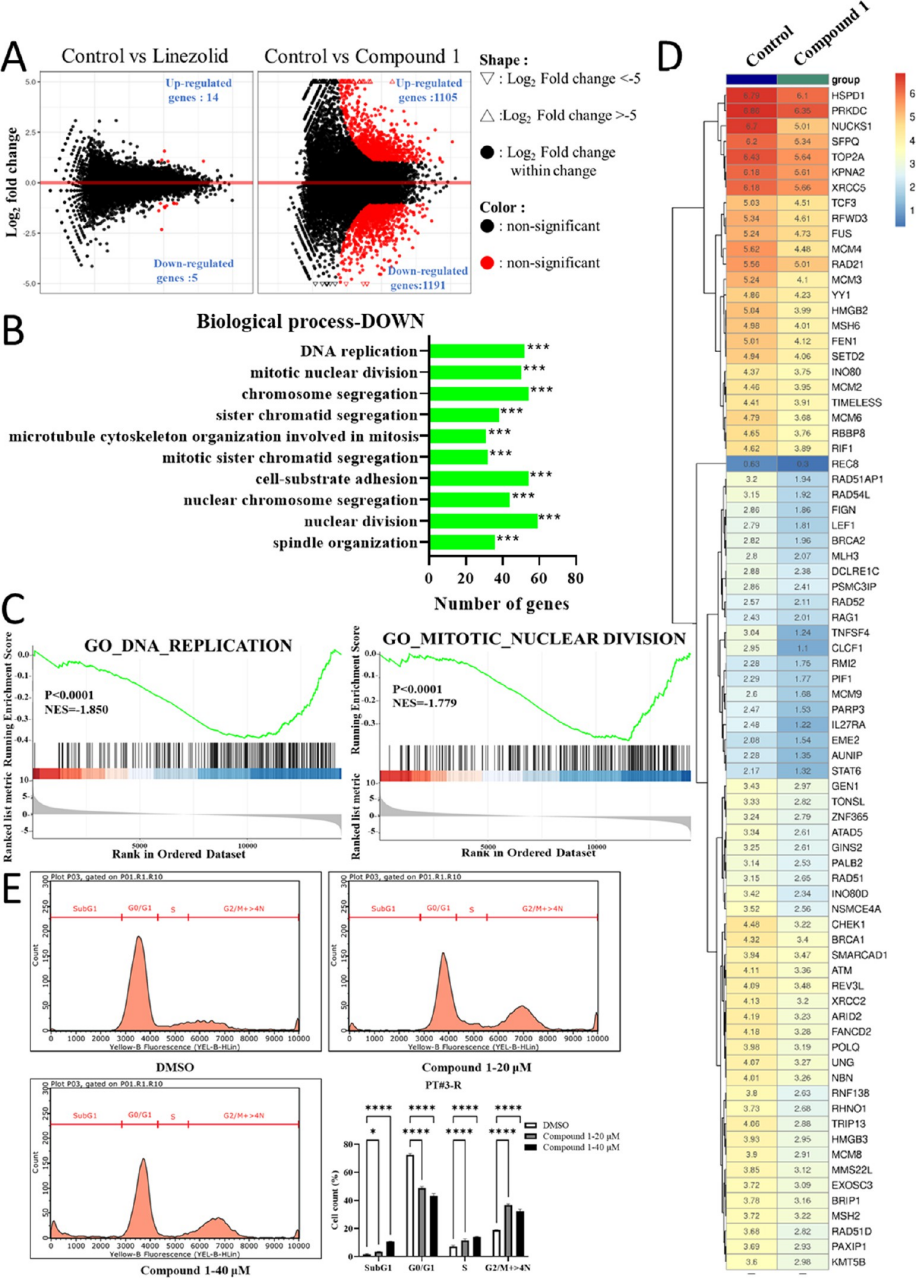
Compound **1** inhibits the growth of TMZ-resistant GBM by suppressing DNA physiological functions. (A) Differential gene expression in PT#3R GBM cells treated with 10 μM compound **1** for 72 h. (B) Analysis based on Gene Ontology (GO) biological processes and (C) GSEA reveals significant inhibition of DNA replication and mitotic nuclear division, key DNA physiological functions. (D) Heatmap displaying the suppressed expression of genes related to DNA replication. (E) Cell cycle analysis demonstrates that compound **1** treatment leads to G2/M phase arrest and increases in the sub-G1 cell population in PT#3R cells.

To investigate the mechanism of action of compound **1**, we conducted RNA-seq on TMZ-resistant GBM cells treated with this compound. In comparison to treatment with linezolid, a 72 h exposure to compound **1** resulted in the upregulation of 1105 genes and the downregulation of 1191 differential expression genes (DEGs), indicating a significant impact on cellular proliferation dynamics (Figure 4A). Further exploration through gene set enrichment analysis (GSEA) unveiled a profound suppression of DNA replication and mitotic nuclear division capabilities accompanied by a substantial reduction in the expression of genes associated with these processes (Figure 4B–D). To corroborate these findings, cell cycle experiments were conducted, confirming a decrease in the number of cells in the G0/G1 phase upon compound **1** treatment, while there was an increase in cells in the G2/M phase and sub-G1 phase (Figure 4E). These results collectively suggest that compound **1** treatment disrupts normal DNA physiological functions, induces cellular damage, and consequently inhibits cell growth.

### Compound **1** Disrupts DNA Repair Mechanisms in GBM Cells by Suppressing DNA-Repaired Proteins

**Figure 5:**
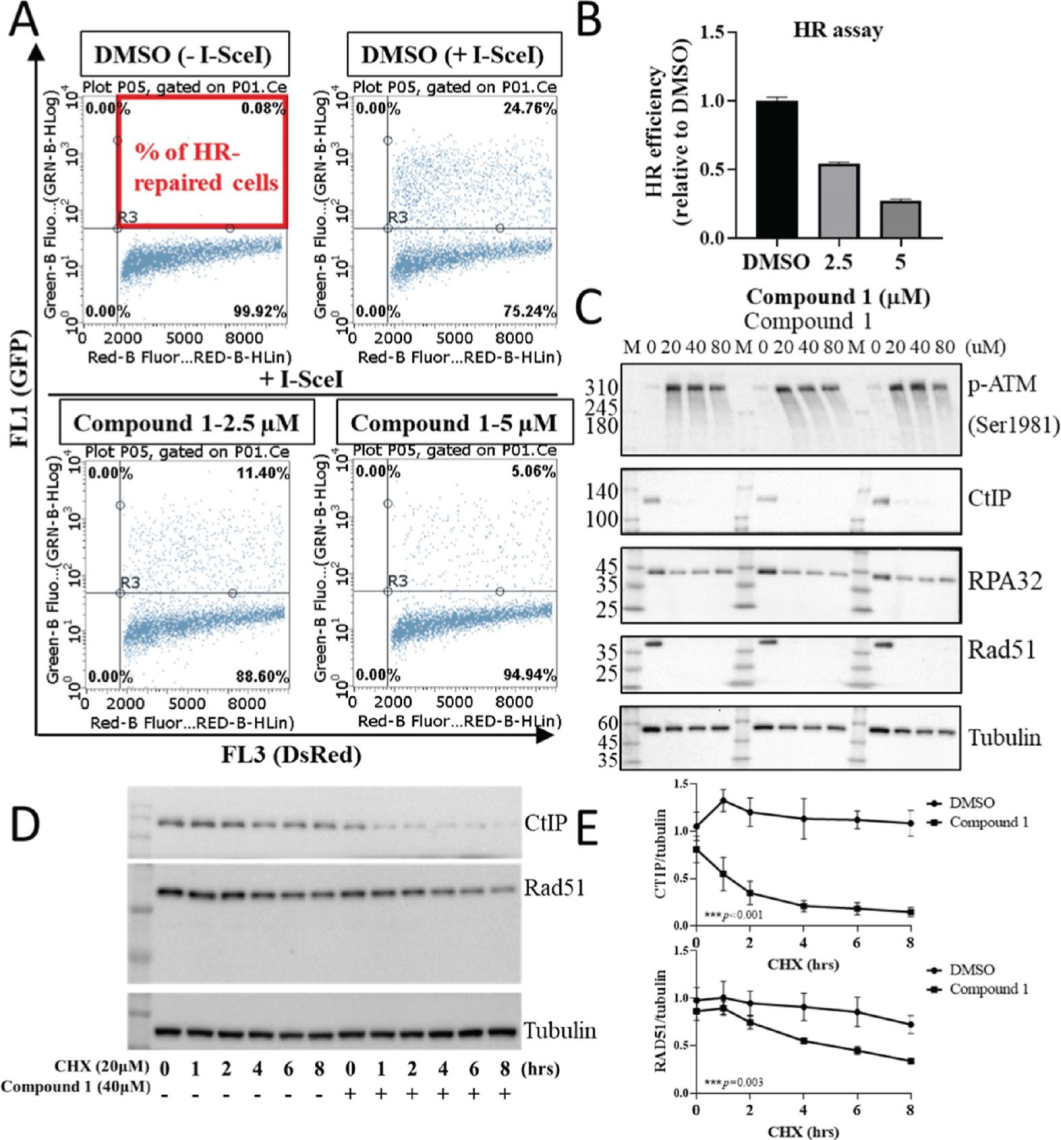
Compound 1 induces DNA repair dysfunction by reducing the stability of DNA repair proteins. (A) HR assay was conducted after treating with 2 and 2.5 μM compound 1 or DMSO for 48 h. (B) Analysis of HR efficiency post-treatment, calculated as the percentage of HR by comparing compound **1** and DMSO treatments, quantified after three independent experiments. (C) Treatment of PT#3-R GBM cells with compound **1** at different concentrations for 72 h. Western blot analysis of the protein expression levels of phospho-ATM, CtIP, RPA32, and RAD51. (D) PT#3-R cells were treated with CHX, and the protein stability of CtIP and Rad51 was assessed in the presence and absence of compound **1**. (E) Quantification was based on the results of (D) after three independent experiments.

To comprehensively evaluate the impact of compound **1** treatment on DNA damage, we conducted a DNA HR assay. HR repair plays a crucial role in the repair of DNA DSBs. However, upon exposure to compound **1**, we observed a substantial decrease in DNA HR repair capability with increasing drug concentrations, indicating the inhibition of DNA repair postcompound **1** treatment (Figure 5A,B). Further mechanistic analysis unveiled a significant increase in the phosphorylation of ataxia telangiectasia-mutated kinase (ATM), a pivotal protein in the DSB repair mechanism. This suggests activation of the physiological response to DSBs induced by compound **1**. Intriguingly, downstream proteins crucial for the repair process, including carboxy-terminal binding protein (CtIP), replication protein A 32 kDa subunit (RPA32), and DNA repair protein RAD51 homologue 1 (RAD51), exhibited significant suppression, signifying that compound **1** inhibits the downstream mechanisms of DNA repair (Figure 5C). Furthermore, when treated with cycloheximide (CHX), a protein synthesis inhibitor disrupting the translocation step in the protein synthesis process, we observed a less pronounced reduction in Rad51 and CtIP protein levels. In contrast, under compound **1** treatment, Rad51 and CtIP protein expression rapidly and significantly decreased over time (Figure 5D,E). This result suggests that compound **1** induces the degradation of Rad51 and CtIP, ultimately leading to cellular death due to the compromised stability of DNA repair proteins.

### Compound **1** Induces Ubiquitination and Degradation of Rad51 Proteins in GBM Cells

**Figure 6:**
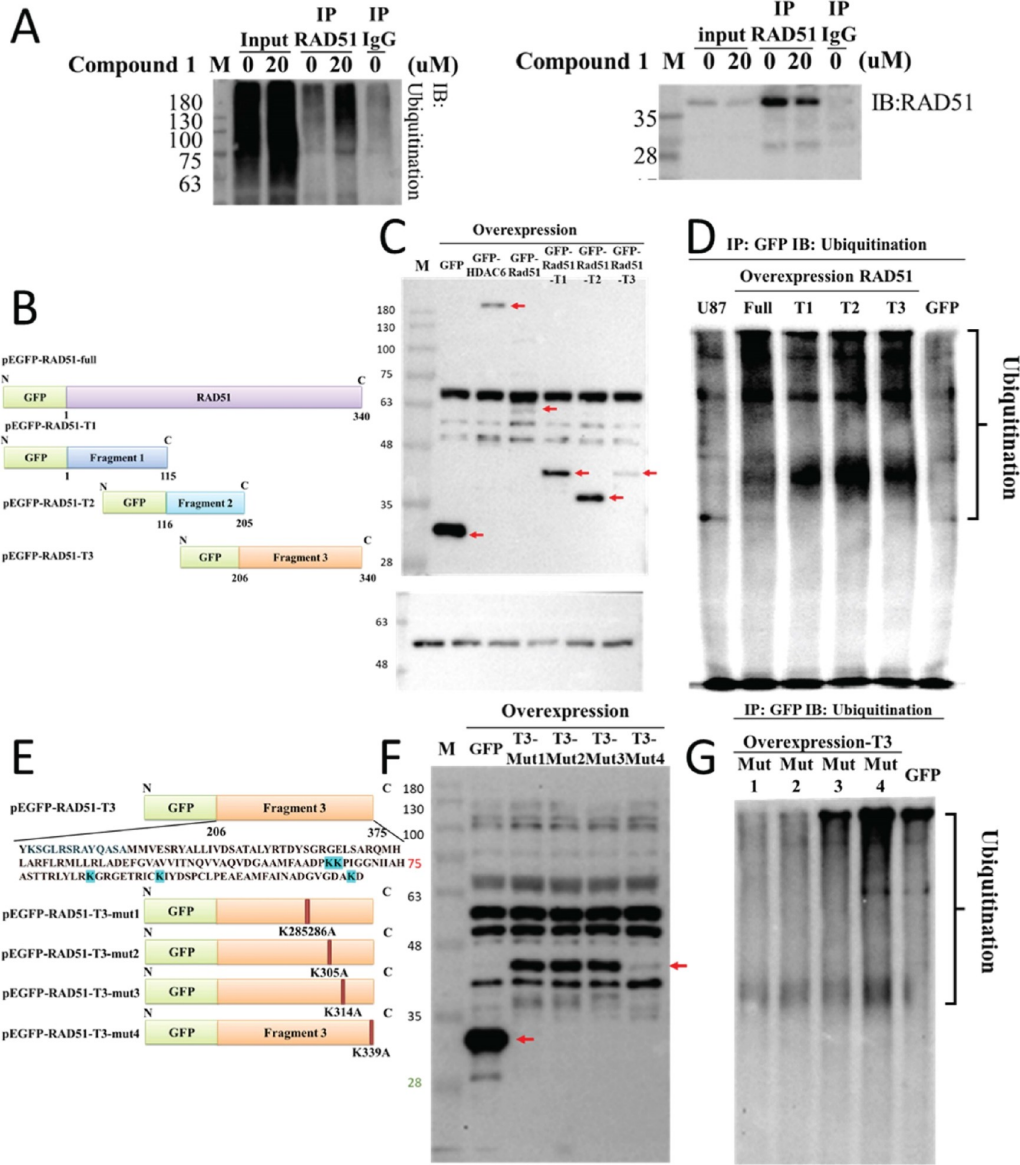
Compound **1** induces ubiquitination and degradation of Rad51 protein. (A) IP conducted using a Rad51 antibody after 24 h of compound **1** treatment in PT#3R GBM cells, confirming the ubiquitination-mediated degradation of Rad51 protein. (B) Schematic representation illustrating truncated Rad51 fragments based on different functional regions of the protein. (C,D) Overexpression of truncated Rad51 fragments in PT#3-R GBM cells, followed by IP with GFP and detection of ubiquitination on the fragments. (E) Plasmid constructs of Rad51 fragment 3 with lysine mutations to alanine. (F,G) Overexpression of the mutated fragment 3 in PT#3-R GBM cells, followed by IP with GFP and detection of changes in ubiquitination.

Ubiquitination, a pivotal physiological process for tagging proteins for degradation, plays a crucial role in cellular regulation. Upon treatment with compound **1**, we immunoprecipitated using an anti-Rad51 antibody to investigate the ubiquitination status of Rad51 protein. The results revealed a significant increase in Rad51 ubiquitination induced by compound **1**, subsequently leading to protein degradation (Figure 6A). To ascertain that this phenomenon is not an off-target effect, we further dissected Rad51 into three functional fragments based on its sequence (Supporting Information V-1). We performed immunoprecipitation (IP) using a GFP protein to detect ubiquitinated proteins. The findings demonstrated that overexpression of Rad51 fragment 3 in pEGFP-RAD51-T3 exhibited the most prominent ubiquitination upon exposure to compound **1** (Figure 6B–D). Moreover, through introduction of mutations at ubiquitination sites within fragment 3, specifically changing lysine residues to alanine (Supporting Information V-2), we observed a reduction in protein ubiquitination at both mutation 1 and mutation 2 positions (Figure 6E–G). These compelling results substantiate that compound **1** triggers the ubiquitination of DNA repair proteins, leading to their subsequent degradation. Consequently, this loss of DSB repair capabilities culminates in GBM cell death.

### Efficiency of Compound **1** in Treating In Vivo GBM and Assessing BBB Penetration

**Figure 7:**
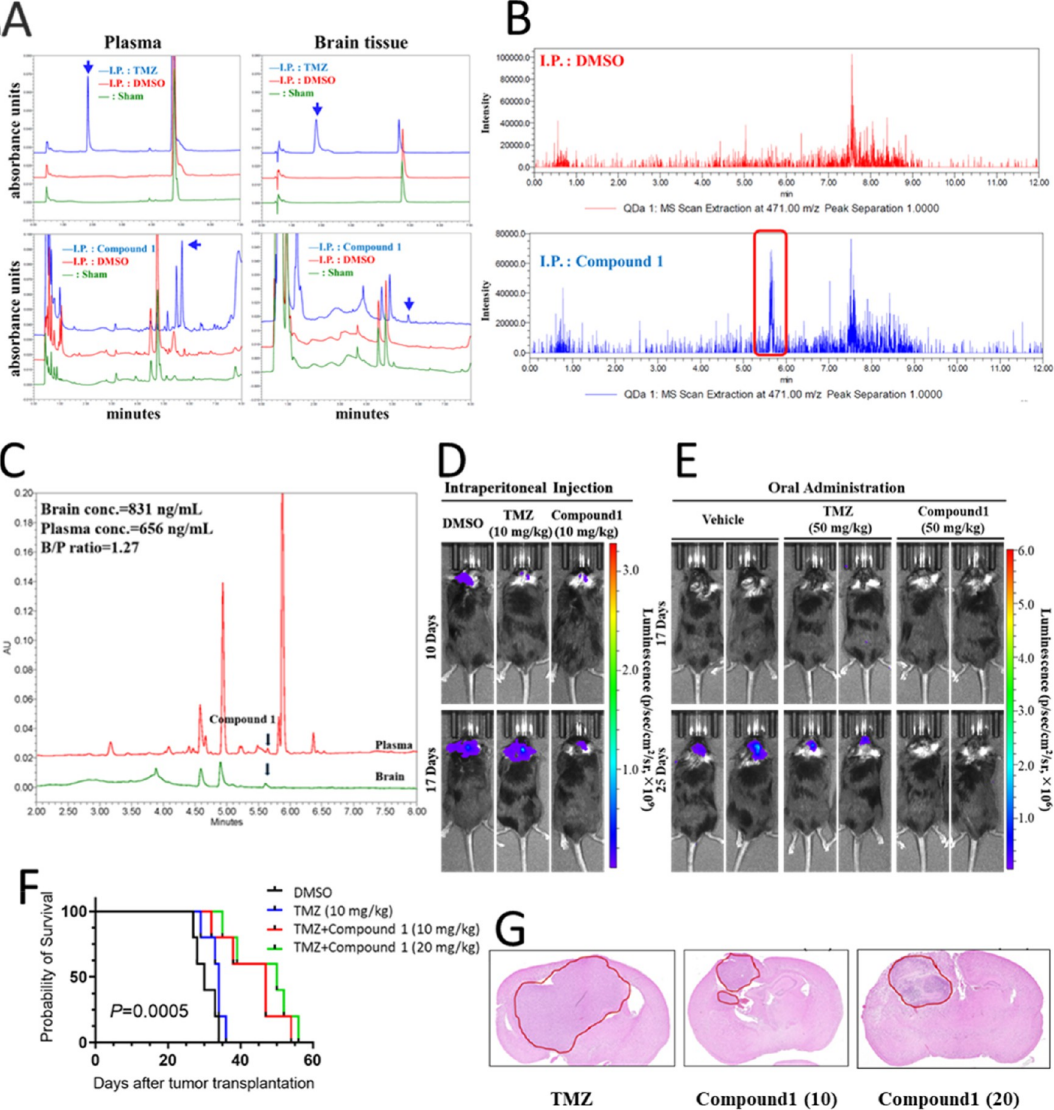
Penetration of compound **1** and its efficacy against TMZ-resistant GBM Cells. (A) UPLC signals corresponding to TMZ or compound **1** detected in plasma and brain tissue extract after intraperitoneal injection, confirmed by (B) mass spectrometry as compound **1** signals. (C) Compound **1** concentration in the samples and the brain/plasma (B/P) ratio. (D) Following orthotopic injection of CT-2A astrocytoma cells, DMSO, 10 mg/kg TMZ, and 10 mg/kg compound **1** were administered intraperitoneally, and tumor growth was monitored using in vivo imaging system (IVIS). (E) After orthotopic injection of CT-2A-R astrocytoma cells, vehicle, 50 mg/kg TMZ, or 50 mg/kg compound **1** was administered orally, with tumor growth monitored via IVIS. (F). Mice with orthotopic PT#3-R cell transplants were randomized into groups, followed by intraperitoneal injections of DMSO, 10 mg/kg TMZ alone, or a combination of 10 mg/kg or 20 mg/kg compound **1** with TMZ every 3 days starting from day 7, with survival analyzed by Kaplan–Meier. (G) Evaluation of the growth status of orthotopically transplanted tumors after treatment with TMZ alone or TMZ combined with compound **1**, assessed using HE staining.

The development of drugs for GBM has become increasingly challenging due to the selective control of substances entering the brain imposed by the BBB. Prior to conducting animal experiments, we performed machine learning model predictions, which suggest that structure modifications successfully retain the ability to pass the BBB while also enhancing the potency of compound **1** in treating GBM (Supporting Information VI). To further validate the effectiveness of compound **1** in vivo GBM treatment, we employed UPLC analysis. Fortunately, no significant overlaps compared compound **1** signal with either wild-type mice plasma or brain tissue extraction, which indicated the specificity of the established analysis method (Supporting Information VII-1). Subsequently, we conducted intraperitoneal injections of TMZ at a dose of 60 mg/kg and compound **1** at a concentration of 360 mg/kg. After a 30 min interval, we performed UPLC analysis on plasma and brain tissue extract, which revealed the presence of compound **1** signals in both plasma and brain tissue extract, in contrast to the sham and solvent control mice (Figure 7A). The corresponding compound **1** signal observed on the LC spectrum was then further analyzed with mass detecting at *m*/*z* = 471.0 (M + H+) (Figure 7B).

The resulting retention time corresponded to the LC result, which distinctly identified the peak of compound **1** in brain tissue extract compared to the DMSO-injected control, thereby demonstrating its potential to traverse the BBB and enter the brain as a potential therapeutic agent. To quantify the penetration ability of compound **1** through the BBB and ensure precise analysis during sampling, the establishing bioanalysis method has been validated according to the request by the US Food and Drug Administration (FDA) bioanalytical method validation guidance for industry (FDA, 2018). The assessed validation, including specificity, linearity, the lower limit of detection (LLOD), the lower limit of quantification (LLOQ), precision, accuracy, and matrix effect (ME), has all reached the criteria (Supporting Information VII-2–4). The detected concentrations of compound **1** in plasma and brain tissue were 656 and 831 ng/mL, respectively, with a brain/plasma ratio of 1.27 (Figure 7C).

In vivo, orthotopic tumor injections were performed using CT-2A astrocytoma cells with intraperitoneal administration (Figure 7D) or TMZ-resistant CT-2A cells with oral administration (Figure 7E). IVIS analysis confirmed that compound 1 effectively inhibited the growth of orthotopic tumors. Additionally, using PT#3-R GBM cells in mice, compound **1** demonstrated its potential to extend the survival rate in the in situ transplantation model of GBM cells (Figure 7F). Post-mortem brain tissue sections further revealed the effective inhibition of TMZ-resistant GBM cell growth in the brain by compound **1** (Figure 7G). These compelling results provide substantial evidence that compound **1** has the capability to penetrate the BBB and holds promise as a candidate for treating TMZ-resistant GBM without adverse reactions observed in the mice during the treatment period (Supporting Information VIII).

### Pharmacokinetic Study

**Figure 8:**
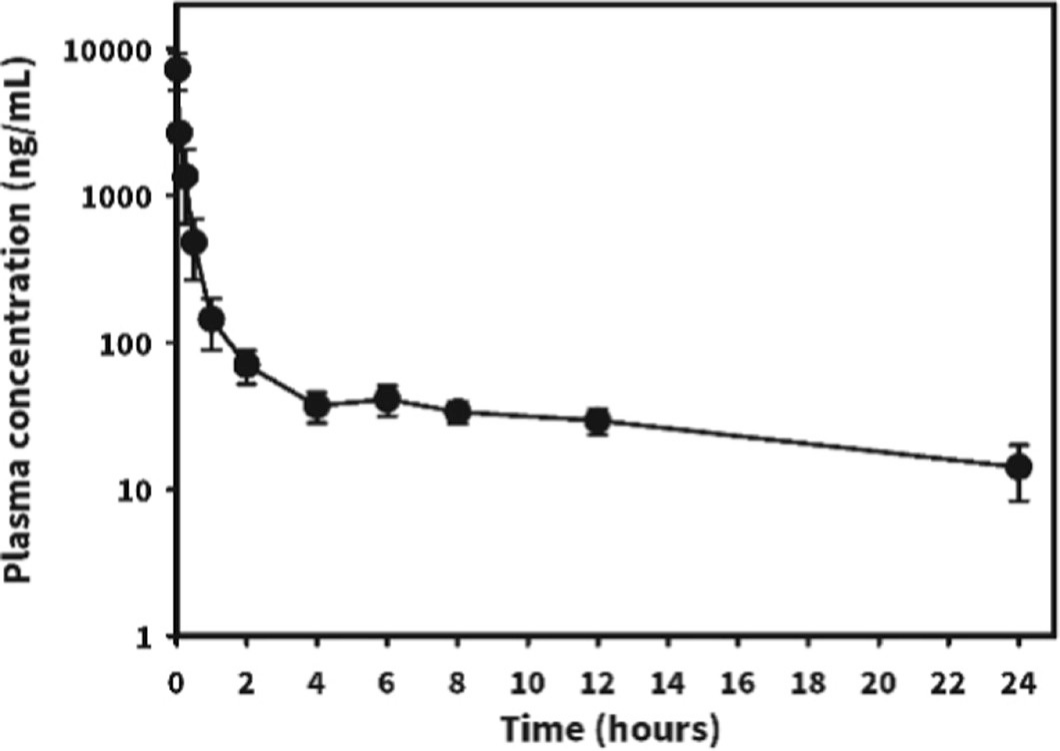
Rat pharmacokinetic profiles of compound 1 at a dosage of 10 mg/kg.

The in vivo pharmacokinetic study of compound **1** was conducted in Sprague–Dawley rats, and the pharmacokinetic profile is depicted in Figure 8. Compound **1** exhibited rapid biodistribution within the first 2 h, followed by a slower elimination phase. Pharmacokinetic parameters were calculated using noncompartmental analysis, as shown in Table 3. Particularly, the elimination half-life (*T*_1/2_) and mean residence time (MRT_inf_) were 12.04 ± 4.42 and 7.91 ± 3.11 h, respectively. These results indicated that compound **1** has a favorable pharmacokinetic profile, characterized by effective tissue penetration and a long circulation half-life, which may reduce the dosing frequency in clinical applications.

**Table 3:** Pharmacokinetic Parameters of Compound **1** at a Dosage of 10 mg/kg^a^

parameter	compound **1**
*C*_0_ (μg/mL)	12.29 ± 8.13
*T*_1/2_ (h)	12.04 ± 4.42
AUC_last_ (h·μg/mL)	2.01 ± 0.53
AUC_inf_ (h·μg/mL)	2.28 ± 0.76
CL (L/h/kg)	4.68 ± 1.33
Vd (L/kg)	76.17 ± 12.28
MRT_inf_	7.91 ± 3.11

a Abbreviations: *C*_0_, initial concentration; *T*_1/2_, elimination half-life; AUC_last_, area under the concentration–time curve from 0 h to the last measurable concentration; AUC_inf_, area under the plasma concentration–time curve extrapolated to infinity; CL, plasma clearance; Vd, volume of distribution; and MRT_inf_, mean residence time extrapolated to infinity.

## Conclusions

To date, TMZ and other alkylating agents remain the mainstay of GBM treatment, but the issue of resistance is pressing. Strobel et al. indicated that TMZ suppresses GBM primarily involves methylating DNA, causing DNA damage that leads to apoptosis. However, due to DNA repair mechanisms, many patients develop resistance during treatment.^1^ Therefore, developing drugs that can counteract the resistant GBM is urgently needed.

In the development of novel therapies, nitrofurans, initially used for treating gastrointestinal diseases caused by bacteria and protozoa, have been repurposed for cancer chemotherapy in colorectal cancer, breast cancer, cervical cancer, and liver cancer, demonstrating the potential of drug repurposing for treating refractory diseases.^9^ Linezolid is absorbed orally and widely distributed in the body, capable of penetrating various tissues and fluids, most importantly crossing the BBB effectively, and acting on central nervous system infections. These properties provide a solid theoretical basis for choosing linezolid as a foundation for GBM treatment.

Among the current linezolid industrial chemical manufacturing control processes, the final step utilized amine compound **15** for N-terminal acetylation to afford linezolid. In this study, we applied compound **15** for further modification instead of acetylation to achieve our anticipated delivery. This indicated that the acquisition of the desired starting material was accessible. Besides, the optimized compound **1**, simply going through reductive amination, amide coupling, and acidic deprotection, also provided an easily handled purification process by precipitation and recrystallization while we bulked up for in vivo studies, indicating that the procedure for producing compound **1** was beneficial for establishing an industrial-grade manufacturing process.

The safety focus in new drug development involves toxicology, pharmacokinetics, and pharmacodynamics studies. Linezolid is absorbed orally and widely distributed in the body and capable of penetrating various tissues and fluids, including the lungs, skin, soft tissues, and CSF. It undergoes hepatic metabolism and is excreted 30% in its original form in urine within 5–7 h, with the remainder as metabolites. Using linezolid requires close monitoring of blood indices and neurological conditions due to potential side effects.^28^ Fisher and Adamson pointed out that current treatments for high-grade malignant gliomas have limited efficacy and significant side effects. Although therapies like bevacizumab and nitrogen mustards have been approved, these methods have limited impact on overall survival and are often accompanied by severe systemic toxicity.^2^ In contrast, our results show that compound **1** has weaker cytotoxic effects on primary cultured neurons and astrocytes. Although the mechanism remains unclear, this suggests that compound **1** selectively targets and kills GBM cells.

In GBM, HDACs inhibit the expression of multiple damage response and repair (DDR) genes. For example, HDAC1 and HDAC2 have been found to suppress the expression of DNA repair genes such as BRCA1 and RAD18.^14^ Additionally, our previous study found that HDAC6 regulates RAD51 and CHEK1 through Sp1 expression, causing dysfunction in DNA repair mechanisms,^29^ thereby affecting tumor cell resistance and survival. This is consistent with our findings that compound **1** enhances TMZ efficacy by inhibiting HDAC, inducing RAD51 ubiquitination, disrupting DNA repair mechanisms, and leading to cell death. This finding highlights the potential of compound **1** in treating TMZ-resistant GBM.

Previous studies have shown that antibody–drug conjugates hold promise for treating GBM but still face challenges in penetrating the BBB.^30^ Dasatinib, a multitargeted tyrosine kinase inhibitor, showed potential in preclinical models. However, its limited efficacy in crossing the BBB and associated toxicity led to its failure in phase I/II clinical trials for recurrent GBM.^29^ Additionally, widely used in oncology, paclitaxel failed in early clinical trials for GBM because it could not effectively cross the BBB. Recent research suggests that combining paclitaxel with strategies to enhance BBB permeability could improve its therapeutic potential for GBM.^31^ Thus, the challenge of crossing the BBB remains a critical barrier in GBM treatment, necessitating the development of novel drug delivery systems and combination therapies to improve drug permeability and efficacy in targeting GBM cells.

So far, compound **1** has displayed promising potency and BBB penetration, and further optimization and evaluation were still necessary to investigate. According to our current studies, the designed derivatives were based on the direct linkage with linezolid. However, the poly ring structure might hinder HDAC inhibition; hence, linker insertion was a latent strategy for optimization.^32,33^ Besides, the structure–activity relationship studies of various HDAC inhibitors have revealed that the surface recognition region modulated the selectivity against different HDAC isoforms,^34−36^ which corresponds to the linezolid moiety in compound **1**; thus, the insertion of the linker also acquired a diverse modification site. On the other hand, the preliminary result has validated the BBB penetration. However, comprehensive pharmacokinetics studies in different species should proceed in future development. Furthermore, due to the ability to penetrate the lipophilic barrier, several toxicity issues should also be considered, including CNS toxicity and long-term brain influence.

Our study primarily explored the role of HDAC inhibitors. Future studies should investigate other potential conjugates. For example, celecoxib, a COX-2 inhibitor, theoretically reduces pro-inflammatory factors in the tumor microenvironment to inhibit tumor growth, but it faces challenges in crossing the BBB, meaning the drug may not reach the tumor site at sufficient concentrations, thus affecting its therapeutic efficacy.^37−39^ Previously, we highlighted the significant role of the arachidonic acid metabolism pathway in TMZ resistance, particularly the upregulation of COX-2, which is associated with GBM progression.^24^ Therefore, combining linezolid with COX-2 inhibitors could further enhance the therapeutic efficacy.

In conclusion, our study demonstrates the substantial potential of linezolid derivatives in GBM treatment by effectively penetrating the BBB and inhibiting tumor cell proliferation, providing new therapeutic options for GBM patients. However, further studies are needed to verify their safety and efficacy in clinical applications.

## Experimental Section

### Chemistry

All chemicals were purchased from commercial suppliers and used without purification, except for compound **27** which was synthesized by our laboratory. Melting points were determined on a Buchi 545 melting point apparatus. Nuclear magnetic resonance (^1^H NMR and ^13^C NMR) spectra were acquired in CDCl_3_, MeOD, or DMSO-*d*_6_ as indicated using a Fourier NMR spectrometer (Bruker Avance, Germany). ^1^H NMR spectral data are presented as illustrated: chemical shift (δ) in ppm, multiplicity [s (singlet), d (doublet), t (triplet), and m (multiple over the range specified)], number of protons, (*n*H), coupling constants (*J*) in hertz, and assignment of protons. High-resolution mass spectra (HRMS) were collected using AB Sciex QStar XL electrospray ionization quadrupole time-of-flight mass spectrometry. The purity of the final compounds was determined by a Shimadzu 2030C-NT HPLC system equipped with an SPD-20A UV detector detecting at a wavelength of 254 nm. The separation was performed at an Agilent ZORBAX Eclipse XDB-C18 column (4.6 mm × 150 mm, 5 μm) with a gradient, elution A: acetonitrile and elution B: water (10 mM ammonium acetate and 0.1% formic acid). The gradient program is as follows: 10% A (initial), 90% A (45 min), 10% A (50 min), and 10% A (60 min). The flow rate was set at 0.5 mL/min, and the injection volume was 20 μL. All of the purity result was found to be ≥ 95%. The characterization data of the compounds has been placed in the Supporting Information.

#### (*S*,*E*)-3-(4-((((3-(3-Fluoro-4-morpholinophenyl)-2-oxooxazolidin-5-yl)methyl)ami-no)methyl)phenyl)acrylic Acid (**16**)

Linezolid-related amine **15** (300 mg, 1.02 mmol, 1 equiv) and 4-formyl cinnamic acid (176 mg, 1.02 mmol, 1 equiv) were suspended in MeOH (25 mL). The resulting reaction mixture was heated up to reflux and stirred for 16 h under a N_2_ atmosphere. The reaction was monitored by TLC. After the reaction was finished, the reaction mixture was cooled down to 0 °C, followed by the addition of sodium borohydride (58 mg, 1.53 mmol, 1.5 equiv) portionwise. A pale-white solid precipitated immediately, and the reaction mixture was stirred for an additional 2 h. After the reaction was finished, the precipitate was collected through filtration to afford the desired product as a pale-white solid (440 mg, 95%). ^1^H NMR (300 MHz, DMSO-*d*_6_): δ 7.52 (dd, *J* = 14.9, 3.3 Hz, 1H), 7.41 (d, *J* = 7.9 Hz, 2H), 7.29 (d, *J* = 8.0 Hz, 2H), 7.22 (dd, *J* = 8.8, 2.5 Hz, 1H), 7.08 (dd, *J* = 9.4, 9.1 Hz, 1H), 7.04 (d, *J* = 15.9 Hz, 1H), 6.33 (d, *J* = 15.8 Hz, 1H), 4.78–4.70 (m, 1H), 4.07 (t, *J* = 8.6 Hz, 1H), 3.83–3.74 (m, 6H), 2.98 (t, *J* = 4.4 Hz, 4H), 2.81–2.75 (m, 2H).

#### (*E*)-3-(4-(((((*S*)-3-(3-Fluoro-4-morpholinophenyl)-2-oxooxazolidin-5-yl)methyl)a-mino)methyl)phenyl)-*N*-((tetrahydro-2*H*-pyran-2-yl)oxy)acrylamide (**17**)

Compound **16** (400 mg, 0.88 mmol, 1 equiv), HOBt (144 mg, 1.05 mmol, 1.2 equiv), and EDCi (254 mg, 1.32 mmol, 1.5 equiv) were suspended in dry DMF (5 mL), and then NMM (0.14 mL, 1.32 mmol, 1.5 equiv) was added. The reaction mixture was stirred for 10 min, and then *O*-(tetrahydro-2*H*-pyran-2-yl) hydroxylamine (124 mg, 1.05 mmol, 1.2 equiv) was added to the reaction mixture and stirred for 16 h under a N_2_ atmosphere. The reaction was monitored by TLC. After the reaction was finished, the reaction mixture was extracted with EA/H_2_O three times and washed with saturated NH_4_Cl(aq) once and brine once. The organic layer was collected and dried over MgSO_4_, and the solvent was removed under reduced pressure to get the crude product. The crude product was purified by column chromatography (DCM/MeOH = 15:1) to afford the desired product as a pale-white solid (340 mg, 70%). ^1^H NMR (300 MHz, DMSO-*d*_6_): δ 11.19 (br s, 1H), 7.52–7.43 (m, 4H), 7.36 (d, *J* = 8.16 Hz, 2H), 7.19 (dd, *J* = 8.4, 1.9 Hz, 1H), 7.04 (dd, *J* = 9.6, 9.2 Hz, 1H), 6.47 (d, *J* = 16.1 Hz, 1H), 4.89 (s, 1H), 4.71 (m, 1H), 4.04 (t, *J* = 8.91 Hz, 1H), 3.95 (m, 1H), 3.80–3.71 (m, 3H), 3.72 (t, *J* = 4.5 Hz, 4H), 3.48–3.56 (m, 1H), 2.95 (t, *J* = 4.7 Hz, 4H), 2.77 (d, *J* = 5.19 Hz, 2H), 1.68 (br s, 3H), 1.52 (br s, 3H).

#### (*S*,*E*)-3-(4-((((3-(3-Fluoro-4-morpholinophenyl)-2-oxooxazolidin-5-yl)methyl)ami-no)methyl)phenyl)-*N*-hydroxyacrylamide (**1**)

**17** (340 mg, 0.62 mmol, 1 equiv) in 5% TFA was dissolved in MeOH (10 mL) and stirred for 16 h at room temperature. The reaction was checked by TLC. After the reaction was finished, the solvent was removed by reduced pressure. The residue was triturated with a mixture of diethyl ether and methanol, filtered, and dried under vacuum to afford the desired product as a pale-white solid. (290 mg, quant.); HPLC purity = 95.14%; mp = 162.2 °C; ^1^H NMR (300 MHz, DMSO-*d*_6_): δ 10.80 (br s, 1H), 9.32 (br s, 1H), 9.11 (br s, 1H)7.63 (d, *J* = 8.2, 2H), 7.53 (d, *J* = 8.3 Hz, 2H), 7.52 (d, *J* = 14.9 Hz, 2H), 7.46 (dd, *J* = 14.9, 2.4 Hz, 1H), 7.17 (dd, *J* = 8.9, 2.1 Hz, 1H), 7.07 (dd, *J* = 9.3, 9.0 Hz, 1H), 6.50 (d, *J* = 15.8 Hz, 1H), 5.04–4.90 (m, 1H), 4.24 (br s, 2H), 4.15 (t, *J* = 9.3 Hz, 1H), 3.80 (dd, *J* = 9.3, 6.6 Hz, 1H), 3.72 (t, *J* = 4.8 Hz, 4H), 2.95 (t, *J* = 4.7 Hz, 4H). ^13^C NMR (150 MHz, DMSO-*d*_6_): δ: 158.85, 158.23, 155.77, 153.93, 136.27, 133.51, 131.06, 128.10, 120.41, 119.70, 118.66, 116.68, 114.84, 107.41, 69.38, 66.57, 51.11, 51.09, 50.83, 49.42, 49.02, 47.93. HRMS calcd for C_24_H_27_FN_4_O_5_, [M + H]^+^ 471.2038; found, 471.2044.

#### Methyl (*S*)-8-(((3-(3-Fluoro-4-morpholinophenyl)-2-oxooxazolidin-5-yl)methyl)a-mino)-8-oxooctanoate (**18**)

Monomethyl suberate (192 mg, 1.02 mmol, 1 equiv) in dry DCM (3 mL) was dissolved, and then oxalyl chloride (0.13 mL, 1.02 mmol, 1 equiv) was added under 0 °C. The resulting reaction mixture was stirred for 2 h at room temperature under a N_2_ atmosphere. After the reaction was finished, the solvent and excess oxalyl chloride were removed under reduced pressure, and the crude product was used in the next step without further purification. The linezolid-related amine **15** (300 mg, 1.02 mmol, 1 equiv) was taken up in dry DCM (3 mL) and added dropwise into crude acyl chloride, followed by pyridine (0.12 mL, 1.53 mmol, 1.5 equiv) under 0 °C. The reaction mixture was warmed to ambient temperature and stirred for 16 h under a N_2_ atmosphere. The reaction was checked by TLC. After the reaction was finished, the reaction mixture was washed with water three times and brine once. The organic layer was collected and dried over MgSO_4_, and the solvent was removed under reduced pressure to get the crude product. The crude product was purified by column chromatography (DCM/MeOH = 15:1) to afford the desired product as a pale yellow solid (200 mg, 42%). ^1^H NMR (300 MHz, CDCl_3_): δ 7.45 (dd, *J* = 14.5, 2.6 Hz, 1H), 7.07 (dd, *J* = 8.9, 2.6 Hz, 1H), 6.96 (dd, *J* = 8.9, 8.8 Hz, 1H), 6.02 (dd, *J* = 6.2, 5.8 Hz, 1H), 4.71–4.79 (m, 1H), 4.01 (t, *J* = 9.0 Hz, 1H), 3.87 (t, *J* = 4.7 Hz, 4H), 3.75 (dd, *J* = 9.1, 6.5 Hz, 1H), 3.67–3.63 (m, 2H), 3.65 (s, 3H), 3.06 (t, *J* = 4.6 Hz, 4H), 2.36–2.16 (m, 4H), 1.63–1.54 (m, 2H), 1.36–1.33(m, 2H), 1.30–1.25 (m, 4H).

#### (*S*)-8-(((3-(3-Fluoro-4-morpholinophenyl)-2-oxooxazolidin-5-yl)methyl)amino)-8-oxooctanoic Acid (**19**)

To a solution of **18** (500 mg, 1.08 mmol, 1 equiv) in dioxane(18 mL) was added 1N LiOH(aq) (1.07 mL, 2.7 mmol, 2.5 equiv). The resulting reaction mixture was stirred for 16 h at room temperature. The reaction was checked by TLC. After the starting material fully transformed to lithium salt, dioxane was removed under reduced pressure. Then, the reaction mixture was adjusted to pH < 2 by using 3 N HCl(aq), and a white solid precipitated. The precipitate was filtered, collected, and dried under vacuum to afford the desired product as a white solid (310 mg, 64%). ^1^H NMR (300 MHz, DMSO-*d*_6_): δ 8.19 (t, *J* = 6 Hz, 1H), 7.50 (dd, *J* = 15.1, 2.6 Hz, 1H), 7.18 (dd, *J* = 8.9, 2.6 Hz, 1H), 7.07 (dd, *J* = 9.3, 9.0 Hz, 1H), 4.78–4.69 (m, 1H), 4.09 (t, *J* = 9.2 Hz, 1H), 3.75 (t, *J* = 4.6 Hz, 4H), 3.72–3.69 (m, 1H), 3.54–3.38 (m, 2H), 2.97 (t, *J* = 4.7 Hz, 4H), 2.27–2.06 (m, 4H), 1.46–1.42 (m, 4H), 1.25–1.17 (m, 4H).

#### *N*1-(((*S*)-3-(3-Fluoro-4-morpholinophenyl)-2-oxooxazolidin-5-yl)methyl)-*N*8-((tetrahydro-2*H*-pyran-2-yl)oxy)octanediamide (20)

**19** (260 mg, 0.58 mmol, 1 equiv), HOBt (93 mg, 0.69 mmol, 1.2 equiv), and EDCi (165 mg, 0.86 mmol, 1.5 equiv) were suspended in dry DMF (5 mL), and then NMM (0.1 mL, 0.86 mmol, 1.5 equiv) was added. The reaction mixture was stirred for 10 min, and then *O*-(tetrahydro-2*H*-pyran-2-yl) hydroxylamine (118 mg, 0.69 mmol, 1.2 equiv) was added to the reaction mixture and stirred for 16 h under a N_2_ atmosphere. The reaction was monitored by TLC. After the reaction was finished, the reaction mixture was extracted with EA/H_2_O three times and washed with saturated NH_4_Cl(aq) once and brine once. The organic layer was collected and dried over MgSO_4_, and the solvent was removed under reduced pressure to get the crude product. The crude product was purified by column chromatography (DCM/MeOH = 15:1) to afford the desired product as a yellow solid (320 mg, quant.) ^1^H NMR (300 MHz, DMSO-*d*_6_): δ 10.88 (s, 1H), 8.19 (t, *J* = 6.2 Hz, 1H), 7.50 (dd, *J* = 15.1, 2.5 Hz, 1H), 7.18 (dd, *J* = 9.1, 2.7 Hz, 1H), 7.08 (dd, *J* = 9.3, 9.1 Hz, 1H), 4.81 (br s, 1H), 4.78–4.70 (m, 1H), 4.10 (t, *J* = 9.0 Hz, 1H), 3.93–3.89 (m, 1H), 3.75 (t, *J* = 4.4 Hz, 4H), 3.72–3.69 (m, 1H), 3.53–3.38 (m, 3H), 2.98 (t, *J* = 4.7 Hz, 4H), 2.11 (t, *J* = 7.3 Hz, 2H), 1.95 (t, *J* = 7.4 Hz, 2H), 1.66–1.43 (m, 10H), 1.18 (m, 4H).

#### (S)-*N*1-((3-(3-Fluoro-4-morpholinophenyl)-2-oxooxazolidin-5-yl)methyl)-*N*8-hydroxyoctanediamide (**2**)

**20** (320 mg, 0.58 mmol, 1 equiv) was dissolved in 5% TFA in MeOH (10 mL) and stirred for 16 h at room temperature. The reaction was checked by TLC. After the reaction was finished, the solvent was removed by reduced pressure. The crude product was purified by column chromatography (DCM/MeOH = 15:1) to afford the desired product as an orange solid (190 mg, 70%); HPLC purity = 99.12%; mp = 136.0 °C; ^1^H NMR (300 MHz, DMSO-*d*_6_): δ 10.30 (br s, 1H), 8.63 (br s, 1H), 8.17 (t, *J* = 5.9 Hz, 1H), 7.48 (dd, *J* = 15.1, 2.5 Hz, 1H), 7.16 (dd, *J* = 8.7, 2.3 Hz, 1H), 7.05 (dd, *J* = 9.3, 9.1 Hz, 1H), 4.74–4.68 (m, 1H), 4.07 (t, *J* = 8.9 Hz, 1H), 3.73 (t, *J* = 4.5 Hz, 4H), 3.70–3.68 (m, 1H), 3.50–3.38 (m, 2H), 2.95 (t, *J* = 4.6 Hz, 4H), 2.06 (t, *J* = 7.4 Hz, 2H), 1.89 (t, *J* = 7.4 Hz, 2H), 1.40 (br s, 4H), 1.15 (br s, 4H). ^13^C NMR (150 MHz, DMSO-*d*_6_): δ: 173.53, 169.53, 155.83, 154.50, 135.96, 133.90, 119.70, 114.45, 107.09, 71.99, 66.59, 51.15, 49.03, 47.65, 41.70, 35.67, 32.65, 21.11, 28.77, 25.64. HRMS calcd for C_22_H_32_FN_4_O_6_, [M + H]^+^ 467.2300; found, 467.2307.

#### (*S*)-2-(4-((((3-(3-Fluoro-4-morpholinophenyl)-2-oxooxazolidin-5-yl)methyl)ami-no)methyl)phenyl)acetic Acid (**21**)

Linezolid-related amine **15** (500 mg, 1.69 mmol, 1 equiv) and 4-formylbenzoic acid (254 mg, 1.69 mmol, 1 equiv) were suspended in MeOH (35 mL). The resulting reaction mixture was heated up to reflux and stirred for 16 h under a N_2_ atmosphere. The reaction was monitored by TLC. After the starting material vanished, the reaction mixture was cooled down to 0 °C, sodium borohydride (58 mg, 1.53 mmol, 1.5 equiv) was added, and the mixture stirred for an additional 2 h. After the reaction was finished, the solvent was removed under reduced pressure to afford a yellow solid (1.13 g, crude). The crude product was used in the next step without further purification.

#### 4-(((((*S*)-3-(3-Fluoro-4-morpholinophenyl)-2-oxooxazolidin-5-yl)methyl)amin-o)methyl)-*N*-((tetrahydro-2*H*-pyran-2-yl)oxy)benzamide (**22**)

Crude product **21** (1.13 g), HOBt (273 mg, 2.02 mmol, 1.2 equiv), and EDCi (487 mg, 2.54 mmol, 1.5 equiv) were suspended in dry DMF (10 mL), and then NMM (0.28 mL, 2.54 mmol, 1.5 equiv) was added. The resulting reaction mixture was stirred for 10 min; then, *O*-(tetrahydro-2*H*-pyran-2-yl) hydroxylamine (237 mg, 2.02 mmol, 1.2 equiv) was added to the reaction mixture and stirred for 16 h under a N_2_ atmosphere. The reaction was monitored by TLC. After the reaction was finished, the reaction mixture was extracted with EA/H_2_O three times and washed with saturated NH_4_Cl(aq) once and brine once. The organic layer was collected and dried over MgSO_4_, and the solvent was removed under reduced pressure to get the crude product. The crude product was purified by column chromatography (DCM/MeOH = 15:1) to afford the desired product as a pale-white solid (720 mg, 80%). ^1^H NMR (300 MHz, DMSO-*d*_6_): δ 11.61 (s, 1H), 7.74 (d, *J* = 8.2 Hz, 2H), 7.53 (dd, *J* = 14.9, 2.4 Hz, 1H), 7.44 (d, *J* = 8.2 Hz, 2H), 7.22 (dd, *J* = 8.4, 1.7 Hz, 1H), 7.08 (dd, *J* = 9.2, 9.0 Hz, 1H), 5.01 (br s, 1H), 4.79–4.70 (m, 1H), 4.08 (t, *J* = 8.9 Hz, 2H), 3.82 (t, *J* = 4.8 Hz, 3H), 3.76 (t, *J* = 4.9 Hz, 4H), 3.55–3.49 (m, 2H), 2.98 (t, *J* = 4.6 Hz, 1H), 2.80 (d, *J* = 5.6 Hz, 1H), 1.74 (br s, 3H), 1.57 (br s, 3H).

#### (*S*)-4-((((3-(3-Fluoro-4-morpholinophenyl)-2-oxooxazolidin-5-yl)methyl)amin-o)methyl)-*N*-hydroxybenzamide (**3**)

**22** (720 mg, 1.36 mmol, 1 equiv) was dissolved in 5% TFA in MeOH (10 mL) and stirred for 16 h at room temperature. The reaction was checked by TLC. After the reaction was finished, the solvent was removed by reduced pressure. The residue was triturated with a mixture of ethyl acetate and methanol, filtered, and dried under vacuum to afford the desired product as a pale-white solid (380 mg, 63%); HPLC purity = 96.55%; mp = 194.5 °C; ^1^H NMR (300 MHz, DMSO-*d*_6_): δ 11.29 (br s, 1H), 9.30 (br s, 1H), 9.09 (br s, 1H), 7.81 (d, *J* = 8.2 Hz, 2H), 7.57 (d, *J* = 8.2 Hz, 2H), 7.47 (dd, *J* = 15.0, 2.5 Hz, 1H), 7.18 (dd, *J* = 9.2, 2.3 Hz, 1H), 7.07 (dd, *J* = 9.3, 9.2 Hz, 1H), 5.04–4.99 (m, 1H), 4.26 (br s, 2H), 4.16 (t, *J* = 14.9, 2.4 Hz, 1H), 3.80 (dd, *J* = 9.2, 7.9 Hz, 1H), 3.73 (t, *J* = 4.8 Hz, 4H), 2.96 (t, *J* = 4.7 Hz, 4H). ^13^C NMR (150 MHz, DMSO-*d*_6_): δ: 155.77, 154.15, 153.87, 136.28, 133.48, 130.59, 127.58, 119.70, 114.83, 107.41, 69.15, 66.57, 51.11, 50.64, 49.46, 47.88. HRMS calcd for C_24_H_27_FN_4_O_5_, [M + H]^+^ 445.1882; found, 445.1887.

#### (*S*)-*N*-(2-Aminophenyl)-4-((((3-(3-fluoro-4-morpholinophenyl)-2-oxooxazolidin-5-yl)methyl)amino)methyl)benzamide (**4**)

Crude product **21** (670 mg), HOBt (162 mg, 1.21 mmol, 1.2 equiv), and EDCi (288 mg, 1.52 mmol, 1.5 equiv) were suspended in dry DMF (10 mL), and then NMM (0.28 mL, 2.54 mmol, 1.5 equiv) was added. The resulting reaction mixture was stirred for 10 min, and then *o*-phenylenediamine (130 mg, 1.21 mmol, 1.2 equiv) was added to the reaction mixture and stirred for 16 h under a N_2_ atmosphere. The reaction was monitored by TLC. After the reaction was finished, the reaction mixture was extracted with EA/H_2_O three times and washed with saturated NH_4_Cl(aq) once and brine once. The organic layer was collected and dried over MgSO_4_, and the solvent was removed under reduced pressure to get the crude product. The crude product was purified by column chromatography (EA) to afford the desired product as a yellow solid (130 mg, 25%); HPLC purity = 95.07%; mp 136.4 °C ^1^H NMR (300 MHz, DMSO-*d*_6_): δ 9.61 (s, 1H), 7.93 (d, *J* = 8.2 Hz, 2H), 7.51 (dd, *J* = 15.2, 2.6 Hz, 1H), 7.46 (d, *J* = 8.3 Hz, 2H), 7.21 (ddd, *J* = 8.9, 2.5, 0.6 Hz, 1H), 7.16 (dd, *J* = 7.9, 1.4 Hz, 1H), 7.06 (dd, *J* = 10.0, 9.0 Hz, 1H), 6.97 (ddt, *J* = 7.9, 1.5, 0.6 Hz, 1H). ^13^C NMR (75 MHz, DMSO-*d*_6_): δ: 170.55, 165.64, 156.69, 147.70, 144.45, 143.60, 142.24, 140.30, 133.51, 128.31, 127.40, 127.15, 126.92, 123.85, 116.74, 116.61, 115.98, 101.83, 72.93, 66.57, 55.38, 50.79, 49.42, 46.32, 42.05, 22.96. HRMS calcd for C_28_H_31_FN_5_O_4_, [M + H]^+^ 520.2355; found, 520.2351.

#### (*R*)-3-Bromo-*N*-((3-(3-fluoro-4-morpholinophenyl)-2-oxooxazolidin-5-yl)methy-l)benzeneesulfonamide (**23**)

To a solution of linezolid-related amine **15** (600 mg, 2.04 mmol, 1 equiv), pyridine (0.24 mL, 3.06 mmol, 1.5 equiv) in dry DCM (20 mL) and 3-bromobenzenesulfonyl chloride (0.36 mL, 2.44 mmol, 1.2 equiv) were added dropwise at 0 °C and then warmed up to room temperature; the reaction mixture was stirred for 2 h under a N_2_ atmosphere. The reaction was monitored by TLC. After the reaction was finished, the reaction mixture was extracted with EA/H_2_O three times and washed with brine once. The organic layer was collected and dried over MgSO_4_, and the solvent was removed under reduced pressure to get the crude product. The crude product was purified by column chromatography (EA/*n*-hexane = 1:1) to afford the desired product as a white solid (550 mg, 52%). ^1^H NMR (300 MHz, DMSO-*d*_6_): δ 8.27 (br s, 1H), 7.95 (t, *J* = 1.8 Hz, 1H), 7.86 (ddd, *J* = 7.8, 1.9, 0.8 Hz, 1H), 7.81 (ddd, *J* = 7.8, 2.0, 1.1 Hz, 1H), 7.56 (t, *J* = 8.0 Hz, 1H), 7.47 (dd, *J* = 15.2, 2.6 Hz, 1H), 7.15 (dd, *J* = 8.9, 2.6 Hz, 1H), 7.06 (dd, *J* = 9.2, 8.9 Hz, 1H), 4.74–4.66 (m, 1H), 4.07 (t, *J* = 9.2 Hz, 1H), 3.76–3.70 (m, 5H), 3.15 (dd, *J* = 6.8, 5.4 Hz, 2H), 2.96 (t, *J* = 4.6 Hz, 4H).

#### Methyl (*R*,*E*)-3-(3-(*N*-((3-(3-fluoro-4-morpholinophenyl)-2-oxooxazolidin-5-yl)methyl)sulfamoyl)phenyl)acrylate (**24**)

**23** (550 mg, 1.07 mmol, 1 equiv), Pd(OAc)_2_ (22 mg, 0.1 mmol, 0.1 equiv), PPh_3_ (32 mg, 0.2 mmol, 0.2 equiv), and NaHCO_3_ (90 mg, 1.07 mmol, 1eq) were suspended in dry DMF (10 mL), and then methyl acrylate (0.11 mL, 1.28 mmol, 1.2 equiv) and Et_3_N (0.17 mL, 1.07 mmol, 1 equiv) were added. The resulting reaction mixture was heated to 120 °C and stirred for 16 h under a N_2_ atmosphere. The reaction was monitored by TLC. After the reaction was finished, the reaction mixture was filtered through Celite cake and washed with EA, and the filtrate was collected. The solvent was removed under reduced pressure, and the residue was purified by flash column chromatography (DCM/MeOH = 15:1) to afford the product with slice methyl acrylate as a brown solid (630 mg, crude). ^1^H NMR (300 MHz, DMSO-*d*_6_): δ 8.18 (br s, 1H), 8.13 (t, *J* = 1.6 Hz, 1H), 8.03 (d, *J* = 7.9 Hz, 1H), 7.86 (ddd, *J* = 7.8, 1.9, 1.1 Hz, 1H), 7.76 (d, *J* = 16.1 Hz, 1H), 7.67 (dd, *J* = 7.9, 7.8 Hz, 1H), 7.49 (dd, *J* = 15.1, 2.5 Hz, 1H), 7.16 (dd, *J* = 8.9, 2.6 Hz, 1H), 7.08 (dd, *J* = 9.2, 9.0 Hz, 1H), 6.77 (d, *J* = 16.1 Hz, 1H), 4.78–4.69 (m, 1H), 4.09 (t, *J* = 9.1 Hz, 1H), 3.77 (s, 3H), 3.77–3.73 (m, 5H), 3.21–3.17 (m, 2H), 2.98 (t, *J* = 4.7 Hz, 4H).

#### (*R*,*E*)-3-(3-(*N*-((3-(3-Fluoro-4-morpholinophenyl)-2-oxooxazolidin-5-yl)methyl)sulfamoyl)phenyl)acrylic Acid (**25**)

To a solution of crude **24** (630 mg) in dioxane (20 mL) was added 1N LiOH(aq) (3.13 mL, 3.13 mmol, 2.5 equiv). The resulting reaction mixture was stirred for 16 h at room temperature. The reaction was checked by TLC. After the starting material fully transformed to lithium salt, dioxane was removed under reduced pressure. Then, the reaction mixture was adjusted to pH < 2 by using 3 N HCl(aq), and a white solid precipitated. The precipitate was filtered, collected, and dried under vacuum to afford the desired product as a yellow solid (240 mg, 44%). ^1^H NMR (300 MHz, DMSO-*d*_6_): δ 8.23 (t, *J* = 6.1 Hz, 1H), 8.01 (s, 1H), 7.96 (d, *J* = 7.6 Hz, 1H), 7.82 (d, *J* = 7.7 Hz, 1H), 7.64 (d, *J* = 16.0 Hz, 1H), 7.63 (t, *J* = 7.6 Hz, 1H), 7.47 (dd, *J* = 15.2, 2.4 Hz, 1H), 7.14 (dd, *J* = 8.8, 2.2 Hz, 1H), 7.08 (dd, *J* = 9.1, 9.0 Hz, 1H), 6.63 (d, *J* = 16.1 Hz, 1H), 4.73–4.68 (m, 1H), 4.06 (t, *J* = 9.2 Hz, 1H), 3.78–3.72 (m, 1H), 3.74 (t, *J* = 4.8 Hz, 4H), 3.18–3.12 (m, 2H), 2.97 (t, *J* = 4.6 Hz, 1H).

#### (*E*)-3-(3-(*N*-(((*R*)-3-(3-Fluoro-4-morpholinophenyl)-2-oxooxazolidin-5-yl)meth-yl)sulfamoyl)phenyl)-*N*-((tetrahydro-2*H*-pyran-2-yl)oxy)acrylamide (**26**)

**25** (240 mg, 0.47 mmol, 1 equiv), HOBt (76 mg, 0.56 mmol, 1.2 equiv), and EDCi (136 mg, 0.71 mmol, 1.5 equiv) were suspended in dry DMF (4 mL), and then NMM (0.08 mL, 0.71 mmol, 1.5 equiv) was added. The reaction mixture was stirred for 10 min, and then *O*-(tetrahydro-2*H*-pyran-2-yl) hydroxylamine (66 mg, 0.56 mmol, 1.2 equiv) was added to the reaction mixture and stirred for 16 h under a N_2_ atmosphere. The reaction was monitored by TLC. After the reaction was finished, the reaction mixture was extracted with EA/H_2_O three times and washed with saturated NH_4_Cl(aq) once and brine once. The organic layer was collected and dried over MgSO_4_, and the solvent was removed under reduced pressure to get the crude product. The crude product was purified by column chromatography (DCM/MeOH = 15:1) to afford the desired product as a yellow solid (240 mg, 84%). ^1^H NMR (300 MHz, DMSO-*d*_6_): δ 8.00 (s, 1H), 7.78–7.85 (m, 2H), 7.64 (d, *J* = 153 Hz, 1H), 7.52–7.64 (m, 1H), 7.47 (dd, *J* = 15.0, 2.4 Hz, 1H), 7.15 (dd, *J* = 8.8, 2.3 Hz, 1H), 7.06 (dd, *J* = 9.2, 8.9 Hz, 1H), 6.63 (d, *J* = 15.7 Hz, 1H), 4.93 (br s, 1H), 4.73–4.67 (m, 1H), 4.07 (t, *J* = 9.5 Hz, 1H), 3.99–3.90 (m, 1H), 3.77–3.72 (m, 1H), 3.74 (t, *J* = 4.9 Hz, 4H), 3.54 (d, *J* = 12.4 Hz, 1H), 3.18–3.14 (m, 2H), 2.97 (t, *J* = 4.9 Hz, 4H), 1.70 (br s, 3H), 1.55 (br s, 1H).

#### (*R*,*E*)-3-(3-(*N*-((3-(3-Fluoro-4-morpholinophenyl)-2-oxooxazolidin-5-yl)methyl)su-lfamoyl)phenyl)-*N*-hydroxyacrylamide (**5**)

**26** (240 mg, 0.40 mmol, 1 equiv) was dissolved in 5% TFA in MeOH (10 mL) and stirred for 16 h at room temperature. The reaction was checked by TLC. After the reaction was finished, the solvent was removed by reduced pressure. The residue was triturated with a mixture of ethyl acetate and methanol, filtered, and dried under vacuum to afford the desired product as an orange solid (170 mg, 82%); HPLC purity = 95.07%; mp = 123.0 °C; ^1^H NMR (300 MHz, DMSO-*d*_6_): δ 8.17 (t, *J* = 6.2 Hz, 1H), 7,97 (s, 1H), 7.80 (dd, *J* = 9.3, 8.8 Hz, 2H), 7.62 (t, *J* = 7.8 Hz, 1H), 7.51 (d, *J* = 15.1 Hz, 1H), 7.46 (dd, *J* = 15.1, 2.4 Hz, 1H), 7.14 (dd, *J* = 8.9, 2.3 Hz, 1H), 7.05 (dd, *J* = 9.2, 9.0 Hz, 1H), 6.56 (d, *J* = 15.1 Hz, 1H), 4.74–4.65 (m, 1H), 4.06 (t, *J* = 9.1 Hz, 1H), 3.76–3.71 (m, 1H), 3.72 (t, *J* = 5.0 Hz, 1H), 3.16–3.10 (m, 2H), 2.95 (t, *J* = 4.7 Hz, 4H). ^13^C NMR (75 MHz, DMSO-*d*_6_): δ: 156.67, 154.38, 153.44, 141.62, 136.44, 136.08, 135.96, 133.89, 133.74, 132.14, 130.53, 127.43, 125.14, 119.77, 114.53, 107.24, 106.90, 77.67, 66.63, 60.23, 51.20, 46.35, 45.65, 21.23, 14.56. HRMS calcd for C_23_H_26_FN_4_O_7_S, [M + H]^+^ 521.1501; found, 521.1508.

#### (*S*)-*N*-((3-(3-Fluoro-4-morpholinophenyl)-2-oxooxazolidin-5-yl)methyl)-3,4,5-trimethoxybenzamide (**6**)

To a solution of linezolid-related amine **15** (300 mg, 1.02 mmol, 1 equiv), pyridine (0.13 mL, 1.33 mmol, 1.3 equiv) in dry DCM (5.7 mL) and 3,4,5-trimethoxybenzoyl chloride (282 mL, 1.22 mmol, 1.2 equiv) were added dropwise at 0 °C. The resulting reaction mixture was warmed up to room temperature and stirred for 2 h under a N_2_ atmosphere. The reaction was monitored by TLC. After the reaction was finished, the reaction mixture was extracted with EA/H_2_O three times and washed with brine once. The organic layer was collected and dried over MgSO_4_, and the solvent was removed under reduced pressure to get the crude product. The crude product was purified by column chromatography (EA/MeOH = 1:1) to afford the desired product as a white solid (250 mg, 52%); HPLC purity = 99.89%; mp = 121.3; ^1^H NMR (300 MHz, DMSO-*d*_6_): δ 8.81 (t, *J* = 5.9 Hz, 1H), 7.50 (dd, *J* = 15.0, 2.5 Hz, 1H), 7.21 (dd, *J* = 8.8, 2.3 Hz, 1H), 7.18 (s, 2H), 7.07 (dd, *J* = 9.4, 9.0 Hz, 1H), 4.91–4.80 (m, 1H), 4.17 (t, *J* = 9.1 Hz, 1H), 3.86 (dd, *J* = 9.2, 7.5 Hz, 1H), 3.82 (s, 6H), 3.75 (t, *J* = 4.8 Hz, 4H), 3.71 (s, 3H), 3.64 (t, *J* = 5.5 Hz, 2H), 2.97 (t, *J* = 4.6 Hz, 1H). ^13^C NMR (75 MHz, DMSO-*d*_6_): δ: 166.95, 156.67, 154.59, 153.03, 140.57, 136.08, 135.96, 133.96, 133.82, 129.64, 119.73, 114.61, 107.30, 106.96, 105.37, 71.97, 66.63, 60.55, 56.43, 51.20, 47.99, 42.93. HRMS calcd for C_24_H_29_FN_3_O_7_, [M + H]^+^ 490.1984; found, 490.1991.

#### (*S*)-5-Chloro-*N*-((3-(3-fluoro-4-morpholinophenyl)-2-oxooxazolidin-5-yl)methyl)-2-hydroxybenzamide (**7**)

5-Chlorosalicylic acid (211 mg, 1.22 mmol, 1.2 equiv), m HOBt (165 mg, 1.22 mmol, 1.2 equiv), and EDCi (293 mg, 1.53 mmol, 1.5 equiv) were suspended in dry DMF (4 mL), and then NMM (0.13 mL, 1.22 mmol, 1.5 equiv) was added. The reaction mixture was stirred for 10 min, and then linezolid-related amine **15** (300 mg, 1.02 mmol, 1 equiv) was added to the reaction mixture and stirred for 16 h under a N_2_ atmosphere. The reaction was monitored by TLC. After the reaction was finished, the reaction mixture was extracted with EA/H_2_O three times and washed with saturated NH_4_Cl(aq) once and brine once. The organic layer was collected and dried over MgSO_4_, and the solvent was removed under reduced pressure to get the crude product. The crude product was purified by column chromatography (EA/*n*-hexane = 1:2) to afford the desired product as a yellow solid (230 mg, 50%); HPLC purity = 99.69%; mp = 239.0 °C; ^1^H NMR (300 MHz, DMSO-*d*_6_): δ 12.22 (br s, 1H), 9.11 (t, *J* = 5.0 Hz, 1H), 7.91 (d, *J* = 2.7 Hz, 1H), 7.49 (dd, *J* = 15.1, 2.5 Hz, 1H), 7.45 (dd, *J* = 8.9, 2.6 Hz, 1H), 7.20 (dd, *J* = 8.6, 2.0 Hz, 1H), 7.07 (dd, *J* = 9.3, 9.0 Hz, 1H), 6.97 (d, *J* = 8.9 Hz, 1H), 4.93–4.84 (m, 1H), 4.17 (t, *J* = 9.0 Hz, 1H), 3.84 (dd, *J* = 9.0, 7.6 Hz, 1H), 3.75 (t, *J* = 4.8 Hz, 4H), 3.69 (t, *J* = 5.6 Hz, 2H), 2.97 (t, *J* = 4.7 Hz, 1H). ^13^C NMR (75 MHz, DMSO-*d*_6_): δ 168.04, 158.38, 156.67, 154.47, 153.44, 136.12, 133.91, 128.32, 122.96, 119.70, 117.78, 114.65, 107.38, 107.03, 71.60, 66.63, 51.19, 48.00, 42.53. HRMS calcd for C_21_H_22_ClFN_3_O_5_, [M + H]^+^ 450.1227; found, 450.1227.

#### (*S*)-2,4-Bis(benzyloxy)-*N*-((3-(3-fluoro-4-morpholinophenyl)-2-oxooxazolidin-5-yl)methyl)-5-isopropylbenzamide (**28**)

**27** (459 mg, 1.22 mmol, 1.2 equiv), HOBt (165 mg, 1.22 mmol, 1.2 equiv), and EDCi (293 mg, 1.53 mmol, 1.5 equiv) were suspended in dry DMF (4 mL), and then NMM (0.13 mL, 1.22 mmol, 1.5 equiv) was added. The reaction mixture was stirred for 10 min, and then linezolid-related amine **15** (300 mg, 1.02 mmol, 1 equiv) was added to the reaction mixture and stirred for 16 h under a N_2_ atmosphere. The reaction was monitored by TLC. After the reaction was finished, the reaction mixture was extracted with EA/H_2_O three times and washed with saturated NH_4_Cl(aq) once and brine once. The organic layer was collected and dried over MgSO_4_, and the solvent was removed under reduced pressure to get the crude product. The crude product was purified by column chromatography (DCM) to afford the desired product as a white solid (350 mg, 53%). ^1^H NMR (300 MHz, DMSO-*d*_6_): δ 8.36 (t, *J* = 5.8 Hz, 1H), 7.63 (s, 1H), 7.50–7.30 (m, 11H), 7.16 (dd, *J* = 8.7, 3.2 Hz, 1H), 7.04 (dd, *J* = 9.3, 9.1 Hz, 1H), 6.88 (s, 1H), 5.28 (s, 2H), 5.17 (s, 2H), 4.83–4.77 (m, 1H), 4.07 (t, *J* = 9.21 Hz, 1H), 3.80–3.70 (m, 1H), 3.73 (t, *J* = 5.0 Hz, 4H), 3.63 (q, *J* = 5.4 Hz, 2H), 3.21–3.12 (m, 1H), 2.95 (t, *J* = 4.5 Hz, 4H), 1.11 (d, *J* = 6.9 Hz, 6H).

#### (*S*)-*N*-((3-(3-Fluoro-4-morpholinophenyl)-2-oxooxazolidin-5-yl)methyl)-2,4-dihydroxy-5-isopropylbenzamide (**8**)

**28** (200 mg, 0.31 mmol, 1 equiv) was dissolved in MeOH (10 mL), and then 5% Pd/C (cat.) was added. After degassing three times, the resulting reaction mixture was stirred for 16 h under a H_2_ atmosphere. The reaction was monitored by TLC. After the reaction was finished, the reaction mixture was filtered through Celite cake and washed with EA, and the filtrate was collected. The solvent was removed under reduced pressure, and the residue was purified by column chromatography (DCM/MeOH = 15:1) to afford the desired product as a colorless solid (150 mg, quant.); HPLC purity = 98.98%; mp = 113.8 °C; ^1^H NMR (300 MHz, DMSO-*d*_6_): δ 7.56 (s, 1H), 7.48 (dd, *J* = 15.0, 2.5 Hz, 1H), 7.18 (dd, *J* = 9.0, 2.1 Hz, 1H), 7.04 (dd, *J* = 9.3, 8.9 Hz, 1H), 6.26 (s, 1H), 4.87–4.80 (m, 1H), 4.15 (t, *J* = 8.9 Hz, 1H), 3.81 (dd, *J* = 9.1, 7.6 Hz, 1H), 3.73 (t, *J* = 4.8 Hz, 4H), 3.64–3.58 (m, 2H), 3.12–3.01 (m, 1H), 2.95 (t, *J* = 4.7 Hz, 4H), 1.12 (d, *J* = 6.9 Hz, 6H). ^13^C NMR (75 MHz, DMSO-*d*_6_) 170.36, 160.28, 154.53, 136.11, 133.94, 126.67, 125.97, 119.67, 114.66, 107.03, 106.68, 102.87, 71.85, 66.63, 51.20, 48.00, 42.41, 26.43, 23.16. HRMS calcd for C_24_H_29_FN_3_O_6_, [M + H]^+^ 474.2035; found, 474.2045.

#### (*S*)-2,4-Bis(benzyloxy)-*N*-((3-(3-fluoro-4-morpholinophenyl)-2-oxooxazolidin-5-yl)methyl)-5-isopropyl-*N*-methylbenzamide (**29**)

**28** (300 mg, 0.46 mmol, 1 equiv) and 60% NaH in mineral oil (24 mg, 0.6 mmol, 1.3 equiv) were suspended in dry DMF (3 mL). Then, MeI (0.03 mL, 0.6 mmol, 1.3 equiv) was added dropwise at 0 °C. The resulting reaction mixture was warmed up to room temperature and stirred for 8 h under a N_2_ atmosphere. The reaction was monitored by TLC. After the reaction was finished, the reaction mixture was extracted with EA/H_2_O three times and washed with saturated NH_4_Cl(aq) once and brine once. The organic layer was collected and dried over MgSO_4_, and the solvent was removed under reduced pressure to get the crude product. The crude product was purified by column chromatography (DCM/MeOH = 30:1) to afford the desired product as a colorless solid (300 mg, 98%). ^1^H NMR (300 MHz, CDCl_3_): δ 7.40 (dd, *J* = 14.6, 2.6 Hz, 1H), 7.38–7.20 (m, 10H), 7.04 (dd, *J* = 8.6, 2.1 Hz, 1H), 7.03 (s, 1H), 6.90–6.84 (m, 1H), 6.45 (s, 1H), 5.02–4.98 (m, 1H), 4.98 (s, 2H), 4.92 (s, 2H), 3.92–3.82 (m, 2H), 3.84 (t, *J* = 4.7 Hz, 4H), 3.30–3.17 (m, 2H), 3.04–2.98 (m, 5H), 3.01 (s, 3H), 1.15 (d, *J* = 6.9 Hz, 6H).

#### (*S*)-*N*-((3-(3-Fluoro-4-morpholinophenyl)-2-oxooxazolidin-5-yl)methyl)-2,4-dihydroxy-5-isopropyl-*N*-methylbenzamide (**9**)

**29** (300 mg, 0.45 mmol, 1 equiv) was dissolved in MeOH (10 mL), and then 5% Pd/C (cat.) was added. After degassing three times, the resulting reaction mixture was stirred for 16 h under a H_2_ atmosphere. The reaction was monitored by TLC. After the reaction was finished, the reaction mixture was filtered through Celite cake and washed with EA, and the filtrate was collected. The solvent was removed under reduced pressure, and the residue was purified by column chromatography (DCM/MeOH = 60:1) to afford the desired product as a colorless solid (100 mg, 47%); HPLC purity = 99.56%; mp = 118.3 °C; ^1^H NMR (300 MHz, DMSO-*d*_6_): δ 9.50–9.46 (m, 2H), 7.50 (dd, *J* = 15.5, 2.1 Hz, 1H), 7.20 (dd, *J* = 9.2, 2.5 Hz, 1H), 7.08 (dd, *J* = 9.3, 9.2 Hz, 1H), 6.78 (s, 1H), 6.36 (s, 1H), 4.90–5.01 (m, 1H), 4.11 (t, *J* = 9.3 Hz, 1H), 3.92–3.62 (m, 3H), 3.76 (t, *J* = 4.9 Hz, 4H), 3.10–2.99 (m, 1H), 2.98 (t, *J* = 4.6 Hz, 4H), 2.98 (s, 3H), 1.08 (d, *J* = 6.9 Hz, 6H). ^13^C NMR (150 MHz, DMSO-*d*_6_): δ 156.52, 155.84, 154.37, 136.03, 133.83, 125.86, 119.71, 114.75, 107.19, 102.61, 66.59, 51.15, 49.03, 47.86, 31.12, 26.23, 23.03. HRMS calcd for C_25_H_31_FN_3_O_6_, [M + H]^+^ 488.2191; found, 488.2197.

#### (*S*)-2,4-Bis(benzyloxy)-*N*-((3-(3-fluoro-4-morpholinophenyl)-2-oxooxazolidin-5-yl)methyl)-5-isopropyl-*N*-ethylbenzamide (**30**)

**28** (250 mg, 0.38 mmol, 1 equiv) and 60% NaH in mineral oil (20 mg, 0.6 mmol, 1.3 equiv) were suspended in dry DMF (3 mL). Then, EtI (0.04 mL, 0.5 mmol, 1.3 equiv) was added dropwise at 0 °C. The resulting reaction mixture was warmed up to room temperature and stirred for 8 h under a N_2_ atmosphere. The reaction was monitored by TLC. After the reaction was finished, the reaction mixture was extracted with EA/H_2_O three times, and washed with saturated NH_4_Cl(aq) once and brine once. The organic layer was collected and dried over MgSO_4_, and the solvent was removed under reduced pressure to get the crude product. The crude product was purified by column chromatography (DCM/MeOH = 30:1) to afford the desired product as a colorless solid (240 mg, 93%). ^1^H NMR (300 MHz, CDCl_3_): δ 7.45 (dd, *J* = 14.6, 2.4 Hz, 1H), 7.48–7.24 (m, 10H), 7.15–7.05 (m, 1H), 7.08 (s, 1H), 6.99–6.90 (m, 1H), 6.57–6.61 (m, 1H), 5.10–4.93 (m, 5H), 4.17–1.07 (m, 1H), 4.02–3.77 (m, 3H), 3.90 (q, *J* = 4.2 Hz, 4H), 3.47–3.28 (m, 1H), 3.33 (q, *J* = 7.1 Hz, 2H), 3.13–2.98 (m, 4H), 1.22 (d, *J* = 6.8 Hz, 6H), 1.08 (t, *J* = 7.1 Hz, 3H)

#### (*S*)-*N*-((3-(3-Fluoro-4-morpholinophenyl)-2-oxooxazolidin-5-yl)methyl)-2,4-dihydroxy-5-isopropyl-*N*-ethylbenzamide (**10**)

**30** (240 mg, 0.35 mmol, 1 equiv) was dissolved in MeOH (10 mL), and then 5% Pd/C (cat.) was added. After degassing three times, the resulting reaction mixture was stirred for 16 h under a H_2_ atmosphere. The reaction was monitored by TLC. After the reaction was finished, the reaction mixture was filtered through Celite cake and washed with EA, and the filtrate was collected. The solvent was removed under reduced pressure, and the residue was purified by column chromatography (DCM/MeOH = 60:1) to afford the desired product as a colorless solid (160 mg, 91%); HPLC purity = 96.98%; mp = 121.0 °C; ^1^H NMR (300 MHz, DMSO-*d*_6_): δ 9.44 (br s, 2H), 7.50 (dd, *J* = 15.4, 2.6 Hz, 1H), 7.20 (dd, *J* = 9.5, 2.6 Hz, 1H), 7.08 (dd, *J* = 9.3, 9.2 Hz, 1H), 6.76 (s, 1H), 6.37 (s, 1H), 4.99–4.85 (m, 1H), 4.10 (t, *J* = 8.6 Hz, 1H), 3.80–3.70 (m, 3H), 3.76 (t, *J* = 4.9 Hz, 4H), 3.12–3.02 (m, 1H), 2.98 (t, *J* = 4.7 Hz, 4H), 1.09 (d, *J* = 6.9 Hz, 6H), 1.06 (t, *J* = 6.7 Hz, 3H). ^13^C NMR (150 MHz, DMSO-*d*_6_): δ: 156.17, 155.83, 154.41, 152.34, 136.02, 133.87, 125.73, 119.69, 115.19, 114.58, 107.22, 102.69, 72.29, 66.60, 51.16, 49.03, 47.98, 31.12, 26.17, 23.0. HRMS calcd for C_26_H_33_FN_3_O_6_, [M + H]^+^ 502.2348; found, 502.2353.

#### (S)-*N*-((3-(3-Fluoro-4-morpholinophenyl)-2-oxooxazolidin-5-yl)methyl)acetamide (**31**)

Linezolid-related amine **15** (2.5 g, 8.47 mmol, 1 equiv) was dissolved in toluene (37.5 mL), and then acetic anhydride (2.02 mL, 21.18 mmol, 2.5 equiv) was added. The resulting mixture was heated to reflux and reacted for 16 h. After the reaction was finished, the white precipitation was collected through filtration to afford the desired product as a white solid (2.3 g, 80%). ^1^H NMR (300 MHz, DMSO-*d*_6_): δ 8.23 (t, *J* = 5.9, 1H), 7.47 (dd, *J* = 15.0, 2.5, 1H), 7.17 (dd, *J* = 6.2, 2.7, 1H), 7.06 (dd, *J* = 9.6, 9.6), 4.77–4.62 (m, 1H), 4.07 (t, *J* = 9.0, 1H), 3.78–3.63 (m, 5H), 2.99–2.90 (m, 4H), 1.83 (s, 3H).

#### (S)-*N*-((3-(5-Fluoro-4-morpholino-2-nitrophenyl)-2-oxooxazolidin-5-yl)methyl)acetamide (**32**)

Linezolid **31** (2 g, 5.93 mmol, 1eq) was suspended in sulfuric acid (20 mL), and then fuming nitric acid (0.4 mL) was added dropwise under an iced bath; then, the resulting mixture was stirred for an additional 1 h. After the reaction was finished, the reaction was quenched by water and then adjusted to pH > 9. The mixture was extracted with EA/H_2_O three times and brine once. The organic layer was collected and dried over MgSO_4_, and the solvent was removed under reduced pressure to get the crude product. The crude product was used in the next step without further purification. ^1^H NMR (300 MHz, CDCl_3_): δ 7.63 (d, *J* = 8.3, 1H), 7.11 (d, *J* = 12.3, 1H), 6.25 (t, *J* = 6.1, 1H), 4.98–4.85 (m, 1H), 4.07 (t, *J* = 8.6, 1H), 3.95–3.86 (m, 4H), 3.86–3.73 (m, 2H), 3.73–3.61 (m, 1H), 3.24–3.14 (m, 4H), 2.11 (s, 3H).

#### (S)-*N*-((3-(2-Amino-5-fluoro-4-morpholinophenyl)-2-oxooxazolidin-5-yl)methyl)acetamide (**33**)

Crude product **32** was dissolved in MeOH (20 mL), and then 10% Pd/C (cat.) was added. After degassing three times, the resulting reaction mixture was stirred for 16 h under a H_2_ atmosphere. The reaction was monitored by TLC. After the reaction was finished, the reaction mixture was filtered through Celite cake and washed with MeOH, and the filtrate was collected. The solvent was removed under reduced pressure to afford the crude product. The crude product was used in the next step without further purification. ^1^H NMR (300 MHz, DMSO-*d*_6_): δ 8.26 (t, *J* = 5.9, 1H), 6.93 (d, *J* = 13.3, 1H), 6.39 (d, *J* = 8.7, 1H), 5.00 (br s, 2H), 4.78–4.64 (m, 1H), 4.20–2.09 (m, 2H), 3.85 (t, *J* = 8.6, 1H), 3.78–3.67 (m, 4H), 3.56–3.46 (m, 1H), 3.01–2.90 (m, 4H), 1.88 (s, 3H).

#### (S)-*N*1-(2-(5-(Acetamidomethyl)-2-oxooxazolidin-3-yl)-4-Fluoro-5-morpholinophenyl)-*N*8-(benzyloxy)octanediamide (**34**)

**33** (200 mg, 0.57 mmol, 1 equiv), 3-((benzyloxy)amino)-3-oxopropanoic acid (191 mg, 0.683 mmol, 1.2 equiv), HOBt (92 mg, 0.683 mmol, 1.2 equiv), and EDCi (164 mg, 0.85 mmol, 1.5 equiv) were suspended in dry DMF (5.7 mL), and then NMM (0.1 mL, 0.85 mmol, 1.5 equiv) was added. The reaction mixture was stirred for 16 h under a N_2_ atmosphere. The reaction was monitored by TLC. After the reaction was finished, the reaction mixture was extracted with EA/H_2_O three times and washed with saturated NH_4_Cl(aq) once and brine once. The organic layer was collected and dried over MgSO_4_, and the solvent was removed under reduced pressure to get the crude product. The crude product was purified by column chromatography (DCM/MeOH = 30:1) to afford the desired product as a pale-white solid (80 mg, 23%). ^1^H NMR (300 MHz, CD_3_OD): δ 7.64 (d, *J* = 8.4, 1H), 7.50–7.32 (m, 5H), 7.38 (d, *J* = 12.7, 1H), 4.87 (s, 2H), 4.18 (t, *J* = 8.7, 1H), 3.91–3.79 (m, 5H), 3.66–3.58 (m, 2H), 3.21–3.13 (m, 4H), 2.30 (t, *J* = 7.4, 2H), 2.08 (t, *J* = 7.3, 2H), 2.05 (s, 3H), 1.68–1.51 (m, 4H), 1.42–1.26 (m, 4H).

#### (S)-*N*1-(2-(5-(Acetamidomethyl)-2-oxooxazolidin-3-yl)-4-fluoro-5-morpholinophenyl)-*N*8-hydroxyoctanediamide (**11**)

**34** (80 mg, 0.13 mmol, 1 equiv) was dissolved in MeOH (10 mL), and then 10% Pd/C (cat.) was added. After degassing three times, the resulting reaction mixture was stirred for 16 h under a H_2_ atmosphere. The reaction was monitored by TLC. After the reaction was finished, the reaction mixture was filtered through Celite cake and washed with MeOH, and the filtrate was collected. The solvent was removed under reduced pressure to afford the crude product. The crude product was purified by column chromatography (DCM/MeOH = 10:1) to afford the desired product as a pale-white solid (14 mg, 21%); HPLC purity = 97.07%; mp = 141.2 °C; ^1^H NMR (300 MHz, CD_3_OD): δ 7.26 (d, *J* = 9.0, 1H), 7.19 (d, *J* = 12.9), 4.05 (t, *J* = 9.0, 1H), 3.88–3.84 (m, 4H), 3.77–3.67 (m, 1H), 3.60–3.57 (m, 2H), 2.43 (t, *J* = 7.5, 2H), 2.14 (t, *J* = 7.2, 2H), 1.75–1.44 (m, 4H), 1.44–1.41 (m, 4H). ^13^C NMR (150 MHz, CD_3_OD): δ: 172.65, 157.69, 147.69, 146.12, 144.15, 141.59, 140.36, 130.76, 126.93, 115.55, 114.94, 102.22, 73.21, 66.52, 50.67, 49.31, 48.42, 46.47, 41.75, 29.23, 22.78, 21.06. HRMS calcd for C_24_H_35_FN_5_O_7_ [M + H]^+^ 524.2515, found 524.2520.

#### (*S*,*E*)-3-(4-(((2-(5-(Acetamidomethyl)-2-oxooxazolidin-3-yl)-4-fluoro-5-morpholinophenyl)amino)methyl)phenyl)acrylic Acid (**35**)

**33** (200 mg, 0.57 mmol, 1 equiv) and 4-formyl cinnamic acid (100 mg, 0.57 mmol, 1 equiv) were suspended in MeOH (5.7 mL). The resulting reaction mixture was heated up to reflux and stirred for 16 h under a N_2_ atmosphere. The reaction was monitored by TLC. After the reaction was finished, the reaction mixture was cooled down to 0 °C, followed by the addition of sodium cyanoborohydride (240 mg, 2.27 mmol, 4 equiv) portionwise. A pale-white solid precipitated immediately, and the reaction mixture was stirred for additional 2 h. After the reaction was finished, the precipitate was collected through filtration to afford the desired product as a pale-white solid (300 mg, quant.). ^1^H NMR (300 MHz, DMSO-*d*_6_): δ 8.29 (t, *J* = 5.6, 1H), 7.49 (d, *J* = 8.2, 2H), 7.37 (d, *J* = 8.2, 2H), 7.23 (d, *J* = 15.8, 1H), 7.00 (d, *J* = 13.1, 1H), 6.41 (d, *J* = 15.8, 1H), 6.07 (d, *J* = 8.1, 1H), 5.00 (br s, 1H), 4.84–4.65 (m, 1H), 4.35 (d, *J* = 6.2, 2H), 3.90 (t, *J* = 8.6, 1H), 3.76–3.70 (m, 2H), 3.70–3.63 (m, 4H), 3.56–3.49 (m, 1H), 2.98–2.90 (m, 2H), 3.88–3.78 (m, 4H), 1.89 (s, 3H).

#### Tetrahydro-2*H*-pyran-2-yl (*E*)-3-(4-(((2-((*S*)-5-(Acetamidomethyl)-2-oxooxazolidin-3-yl)-4-fluoro-5-morpholinophenyl)amino)methyl)phenyl)acrylate (**36**)

Compound **35** (300 mg, 0.57 mmol, 1 equiv), HOBt (92 mg, 0.68 mmol, 1.2 equiv), and EDCi (164 mg, 0.85 mmol, 1.5 equiv) were suspended in dry DMF (5.7 mL), and then NMM (0.1 mL, 0.85 mmol, 1.5 equiv) was added. The reaction mixture was stirred for 10 min, and then *O*-(tetrahydro-2*H*-pyran-2-yl) hydroxylamine (80 mg, 0.68 mmol, 1.2 equiv) was added to the reaction mixture and stirred for 16 h under a N_2_ atmosphere. The reaction was monitored by TLC. After the reaction was finished, the reaction mixture was extracted with EA/H_2_O three times and washed with saturated NH_4_Cl(aq) once and brine once. The organic layer was collected and dried over MgSO_4_, and the solvent was removed under reduced pressure to get the crude product. The crude product was purified by column chromatography (DCM/MeOH = 15:1) to afford the desired product as a pale-white solid (200 mg, 58%). ^1^H NMR (300 MHz, DMSO-*d*_6_): δ 8.29 (t, *J* = 6.0, 1H), 7.59–7.39 (m, 5H), 7.01 (d, *J* = 13.2, 1H), 7.48 (d, *J* = 16.0, 1H), 6.15–6.01 (m, 2H), 4.91 (br s, 1H), 4.84–4.69 (m, 1H), 4.38 (d, *J* = 6.0, 2H), 4.04–3.84 (m, 2H), 3.72–3.62 (m, 4H), 3.61–3.48 (m, 3H), 2.89–2.75 (m, 4H), 1.89 (s, 3H), 1.78–1.63 (m, 3H), 1.62–1.46 (m, 3H).

#### (*S*,*E*)-3-(4-(((2-(5-(Acetamidomethyl)-2-oxooxazolidin-3-yl)-4-fluoro-5-morpholinophenyl)amino)methyl)phenyl)-*N*-hydroxyacrylamide (**12**)

**36** (200 mg, 0.33 mmol, 1 equiv) was dissolved in 5% TFA in MeOH (10 mL) and stirred for 16 h at room temperature. The reaction was checked by TLC. After the reaction was finished, the solvent was removed by reduced pressure. The residue was triturated with a mixture of diethyl ether and methanol, filtered, and dried under vacuum to afford the desired product as a pale-white solid (151 mg, 87%); HPLC purity = 99.67%; mp = 187.5 °C; ^1^H NMR (300 MHz, DMSO-*d*_6_): δ 8.29 (t, *J* = 6.3, 1H), 7.52 (d, *J* = 8.4, 2H), 7.46–7.39 (m, 3H), 7.00 (d, *J* = 13.2, 1H), 6.43 (d, *J* = 15.9), 6.08–6.04 (m, 2H), 4.78–4.74 (m, 1H), 4.37 (d, *J* = 6.3, 2H), 3.90 (t, *J* = 8.7, 1H), 3.68–3.64 (m, 4H), 2.85–2.83 (m, 4H), 1.89 (s, 3H). ^13^C NMR (150 MHz, DMSO-*d*_6_): δ: 171.36, 164.12, 157.44, 147.66, 146.10, 143.15, 140.99, 139.46, 134.53, 128.87, 119.72, 116.96, 102.62, 73.66, 67.31, 51.58, 50.15, 47.17, 42.80, 23.70. HRMS calcd for C_26_H_31_FN_5_O_6_, [M + H]^+^ 528.2253; found, 524.2272.

#### (*S*)-4-(((2-(5-(Acetamidomethyl)-2-oxooxazolidin-3-yl)-4-fluoro-5-morpholinophenyl)amino)methyl)benzoic Acid (**37**)

**33** (300 mg, 0.85 mmol, 1 equiv) and 4-formylbenzoic acid (128 mg, 0.85 mmol, 1 equiv) were suspended in MeOH (8.5 mL). The resulting reaction mixture was heated up to reflux and stirred for 16 h under a N_2_ atmosphere. The reaction was monitored by TLC. After the starting material vanished, the reaction mixture was cooled down to 0 °C, sodium cyanoborohydride (214 mg, 3.4 mmol, 4 equiv) was added, and the mixture was stirred for an additional 2 h. After the reaction was finished, the solvent was removed under reduced pressure to afford the crude product as a yellow solid. The crude product was used in the next step without further purification. ^1^H NMR (300 MHz, CO_3_OD): δ 7.93 (d, *J* = 7.5, 2H), 7.41 (d, *J* = 8.2, 2H), 6.94 (d, *J* = 12.8, 1H), 6.14 (d, *J* = 8.3, 1H), 4.46 (s, 2H), 4.06 (t, *J* = 8.9, 1H), 3.79–3.70 (m, 4H), 3.67–3.53 (m, 3H), 2.92–2.85 (m, 4H), 2.03 (s, 3H).

#### (*S*)-4-(((2-(5-(Acetamidomethyl)-2-oxooxazolidin-3-yl)-4-fluoro-5-morpholinophenyl)amino)methyl)-*N*-(2-aminophenyl)benzamide (**13**)

Crude product **37** (300 mg, 0.62 mmol, 1 equiv), HOBt (100 mg, 0.74 mmol, 1.2 equiv), and EDCi (180 mg, 0.93 mmol, 1.5 equiv) were suspended in dry DMF (6 mL), and then NMM (0.1 mL, 0.93 mmol, 1.5 equiv) was added. The resulting reaction mixture was stirred for 10 min, and then *o*-phenylenediamine (80 mg, 0.74 mmol, 1.2 equiv) was added to the reaction mixture and stirred for 16 h under a N_2_ atmosphere. The reaction was monitored by TLC. After the reaction was finished, the reaction mixture was extracted with EA/H_2_O three times and washed with saturated NH_4_Cl(aq) once and brine once. The organic layer was collected and dried over MgSO_4_, and the solvent was removed under reduced pressure to get the crude product. The crude product was purified by column chromatography (EA) to afford the desired product as a yellow solid (224 mg, 63%); HPLC purity = 96.79%; mp = 144.5 °C; ^1^H NMR (300 MHz, DMSO-*d*_6_): δ 9.65 (s, 1H), 8.30 (t, *J* = 5.7, 1H), 7.94 (d, *J* = 8.1, 2H), 7.52 (d, *J* = 8.4, 2H), 7.27–7.24 (m, 2H), 7.20–6.90 (m, 2H), 6.81 (d, *J* = 8.1, 1H), 6.63 (t, *J* = 7.2, 1H), 6.15–6.07 (m, 2H), 4.79–4.76 (m, 1H), 4.44 (s, 2H), 3.92 (t, *J* = 8.7, 1H), 3.76–3.66 (m, 5H), 2.96–2.93 (m, 1H), 2.86–2.83 (m, 4H), 1.90 (s, 3H). ^13^C NMR (150 MHz, DMSO-*d*_6_): δ 170.50, 165.58, 156.62, 146.87, 145.31, 144.39, 143.54, 142.19, 140.21, 133.46, 128.24, 127.34, 123.80, 116.69, 115.97, 101.76, 72.86, 66.57, 50.91, 49.38, 49.03, 46.26, 42.00, 31.11, 22.90. HRMS calcd for C_30_H_34_FN_6_O_5_, [M + H]^+^ 577.2569; found, 577.2582.

#### 4-(((2-((*S*)-5-(Acetamidomethyl)-2-oxooxazolidin-3-yl)-4-fluoro-5-morpholinophenyl)amino)methyl)-*N*-((tetrahydro-2*H*-pyran-2-yl)oxy)benzamide (**38**)

Crude product **37** (400 mg, 0.82 mmol, 1.0 equiv), HOBt (132 mg, 0.98 mmol, 1.2 equiv), and EDCi (240 mg, 1.23 mmol, 1.5 equiv) were suspended in dry DMF (8 mL), and then NMM (0.14 mL, 1.23 mmol, 1.5 equiv) was added. The reaction mixture was stirred for 10 min, and then *O*-(tetrahydro-2*H*-pyran-2-yl) hydroxylamine (115 mg, 0.98 mmol, 1.2 equiv) was added to the reaction mixture and stirred for 16 h under a N_2_ atmosphere. The reaction was monitored by TLC. After the reaction was finished, the reaction mixture was extracted with EA/H_2_O three times and washed with saturated NH_4_Cl(aq) once and brine once. The organic layer was collected and dried over MgSO_4_, and the solvent was removed under reduced pressure to get the crude product. The crude product was purified by column chromatography (DCM/MeOH = 15:1) to afford the desired product as a pale white solid (220 mg, 46%) ^1^H NMR (300 MHz, CD_3_OD): δ 7.76 (d, *J* = 8.2, 2H), 7.50 (d, *J* = 8.2, 2H), 6.96 (d, *J* = 12.8, 1H), 6.10 (d, *J* = 8.2), 5.07 (s, 1H), 4.46 (s, 2H), 4.21–3.99 (m, 2H), 3.80–3.70 (m, 4H), 3.70–3.55 (m, 4H), 2.93–2.82 (m, 4H), 2.03 (s, 3H), 1.98–1.76 (m, 3H), 1.77–1.52 (m, 3H).

#### (*S*)-4-(((2-(5-(Acetamidomethyl)-2-oxooxazolidin-3-yl)-4-fluoro-5-morpholinophenyl)amino)methyl)-*N*-hydroxybenzamide (**14**)

**38** (220 mg, 0.38 mmol, 1 equiv) was dissolved in 5% TFA in MeOH (10 mL) and stirred for 16 h at room temperature. The reaction was checked by TLC. After the reaction was finished, the solvent was removed by reduced pressure. The residue was triturated with a mixture of ethyl acetate and methanol, filtered, and dried under vacuum to afford the desired product as an orange solid (176 mg, 93%); HPLC purity = 97.19%; mp = 190.2 °C; ^1^H NMR (300 MHz, CD_3_OD): δ 7.73 (d, *J* = 8.1, 2H),7.51 (d, *J* = 7.8, 2H), 6.96 (d, *J* = 12.9, 1H), 6.11 (d, *J* = 8.4, 1H), 4.50 (s, 2H), 4.05 (t, *J* = 8.7, 1H), 3.78–3.74 (m, 4H), 3.70–3.59 (m, 3H), 2.91–2.87 (m, 4H), 2.03 (s, 3H). ^13^C NMR (150 MHz, CD_3_OD): δ: 173.52, 172.60, 171.52, 156.51, 153.41, 151.78, 139.60, 130.27, 124.94, 120.03, 116.07, 113.80, 73,24, 66.48, 50.49, 49.43, 41.85, 35.87, 32.25, 28.47, 25.21, 21.08. HRMS calcd for C_24_H_29_FN_5_O_6_, [M + H]^+^ 502.2096; found, 524.2103.

### Cell Culture

The human glioblastoma cell lines U87MG (HTB-14), T98G (CRL-1690), and A172 (CRL-1620) were provided by the American Type Culture Collection (Manassas, VA, USA). The patient-derived cell line PT#3 was purified under the approval of the Institutional Review Board of Taipei Medical University (TMU) (No. 201006011). The murine astrocytoma cell line CT-2A (SCC194) was purchased from Merch Millipore (MA, USA). TMZ-resistant cell lines were established as described previously.^40^ All cell lines were cultured in Dulbecco’s modified Eagle’s medium basal medium (Cat# 10-013-CM, CORNING, MA, USA), supplemented with 10% fetal bovine serum (Cat# 12676029, Thermo Fisher Scientific, MA, USA), 100 units/mL penicillin, and 100 μg/mL streptomycin (Cat# 15140122, Thermo Fisher Scientific) at 37 °C in the environment of 95% air and 5% carbon dioxide and maintained resistant characteristic with 50–100 μg of TMZ (Cat# HY-17364, MedChemExpress, NJ, USA). Primary cultured astrocytes and neurons were derived from E17 embryonic mice. The cortex was isolated, dissociated, and cultured in the Neurobasal Medium (cat. no. 21103049, Thermo Fisher Scientific) supplemented with the 1X B-27 Supplement (cat. no. 17504044, Thermo Fisher Scientific) to promote neuronal cell growth. Alternatively, cells were cultured in 10% DMEM to induce astrocyte growth.

### MTT Assay

MTT assay was employed to evaluate the cytotoxicity of the synthesized compound against resistant GBM cells. The MTT (3-(4,5-dimethylthiazol-2-yl)-2,5-diphenyltetrazolium bromide, catalog no. AM0818-0005) powder was purchased from BIONOVAS (Ontario, Canada). The MTT was prepared at 10 mg/mL stock with ultrapure water and then stored at −20 °C. All cells were seeded 2.5 × 10^4^ per well in the 24-well plate for 24 h, followed by treating at different concentrations of compounds for 72 h. Before the experiment, the MTT stock was 10-fold diluted with the culture medium and then incubated with cells for 30 min. After the incubation, the MTT medium was discarded and added 300 μL of dimethyl sulfoxide (DMSO, cat. no. D2650, Sigma-Aldrich, MO, USA) to dissolve the cells. Finally, 100 μL of the mixture was transferred to measure the OD 595 nm absorbance.

### Cell Proliferation Assay

Cell viability and drug cytotoxicity were determined by a CCK-8 kit (Cell Counting Kit-8, CK04-13, MD, USA) (CCK8, C0005, Target Molecule Corp., MA, USA) reagent. Cells were seeded 2 × 10^3^ per well in the 96-well plate for 24 h, followed by treating at different concentrations of compounds for 72 h. Before the experiment, CCK8 was 10-fold diluted with the culture medium and then incubated with cells for 30 min. Finally, the OD was 450 nm absorbance and calculated for cell viability.

### HDAC Enzyme Inhibition Assays

Enzyme inhibition assays were performed by the Reaction Biology Corporation, Malvern, PA (http://www.reactionbiology.com). The substrate for HDAC-1, −2, −8, and −6 is a fluorogenic peptide derived from p53 residues 379–382 [RHKK(Ac)]. Compounds were dissolved in DMSO and tested in a 10-dose IC_50_ mode with a 3-fold serial dilution starting at 10 μM. Trichostatin A was the reference.

### Gene Expression and Survival Rate Analysis

For the analysis of the impact on gene expression and survival rates, the TCGA-GBM database was employed to analyze the expression levels in normal brain tissue and GBM. Furthermore, the CGGA-GBM database was utilized to analyze the expression of class I HDAC in primary and resistant GBM. The analysis of HDAC and survival rates was conducted by using the CGGA database. All these data were processed through the GlioVis Web Site (http://gliovis.bioinfo.cnio.es/).

### Western Blotting

The experimental procedure was performed as described in our previous study.^41^ On the day prior to the experiment, 5 × 10^2^ cells were seeded into each well of a 6-well plate. The following day, the cells were washed twice with PBS and treated with 10 μM compound **1**. The acetylation status of histones H3 and H4, DNA repair, and protein stability assay were assessed at 0–24 h post-treatment. The antibody models and dilution ratios used are detailed in Table S1. Protein image collection and quantification were done by the ChemiDoc Touch Imaging System (Cat. No. 12676029, Bio-Rad Laboratories, Inc., CA, USA).

### Bioinformatics Analysis

After treating PT#3R cell lines with 10 μM compound **1** for 72 h, cells were lysed using the QIAzol Lysis Reagent (Cat# 79306, QIAGEN, Hilden, Germany), and the extraction and analysis were entrusted to BioTools (Taipei, Taiwan). RNA Purity and quantification were checked using SimpliNano—Biochrom Spectrophotometers (Biochrom, MA, USA). RNA degradation and integrity were monitored by a Qsep 100 DNA/RNA Analyzer (BiOptic Inc., Taiwan). A total amount of 1 μg of total RNA per sample was used as an input material for the RNA sample preparations. Sequencing libraries were generated using the KAPA mRNA HyperPrep Kit (KAPA Biosystems, Roche, Basel, Switzerland) following manufacturer’s recommendations, and index codes were added to attribute sequences to each sample. The strand marked with dUTP in not amplified, allowing strand-specific sequencing. At last, PCR products were purified using the KAPA Pure Beads system, and the library quality was assessed on the Qsep 100 DNA/RNA Analyzer (BiOptic Inc.). The library quality was assessed on the Qubit 2.0 Fluorometer (Thermo Scientific) and the Agilent Bioanalyzer 2100 system. At last, the library was sequenced on an Illumina NovaSeq6000 platform, and a 150 bp paired-end reads were generated. The raw data, generated by high-throughput sequencing (Illumina NovaSeq 6000 platform), were initially converted into raw sequenced reads using CASAVA base calling and were stored in FASTQ format. Subsequently, DEGs under two conditions were identified using standard methods. The DEGs were then subjected to GO and Kyoto Encyclopedia of Genes and Genomes pathway enrichment analyses. Simultaneously, the DOSE package was employed for Disease Ontology (DO) enrichment analysis using DO, DisGeNET, and NCG databases. Additionally, GSEA was performed to identify enriched biological functions and activated pathways using a molecular signature database (MSigDB) with 1000 permutations. MSigDB provides annotated gene sets for use with the GSEA software, including hallmark gene sets, positional gene sets, curated gene sets, motif gene sets, computational gene sets, GO gene sets, oncogenic gene sets, and immunologic gene sets.

### Cell Cycle

To measure the cell cycle, cells were seeded 5 × 10^4^ per well in a 12-well plate for 24 h, followed by treatment of the different concentrations of compounds for 72 h. Next, the cells were fixed with 70% ethanol and stored at −20 °C for at least 24 h. After washing, the cells were stained with 30 μg/mL propidium iodide (PI, Cat# P4170, Sigma-Aldrich) and contented 10 ng/mL RNaseA (Cat# R6148, Sigma-Aldrich) for 30 min, followed by detection using flow cytometry Guava easyCyte Flow Cytometers and then analyzed by the Guava easyCyte System 3.3 (Cytec Industries Incorporated, NJ, USA).

### Homologous Recombination Repair Assays

For the DNA, HR repair assay continued to use our previous study.^30^ U87MG cells were cotransfected at a 2:1 ratio using the NHEJ reporter and DsRed-Monomer plasmids. Cells were harvested 2 days after transfection and subjected to flow cytometry analysis by a Guava EasyCyte System. Only DsRed-positive cells were analyzed for HR and NHEJ efficiencies to circumvent possible differences in transfection efficiencies. Data were analyzed to reveal the percentage of GFP-positive cells relative to those of DsRed-positive cells. Data were set to 1% of the background level of GFP-positive cells in every internal control.

### Immunoprecipitation and DNA Ubiquitination Assay

The mRNA coding sequence of RAD51 (NM_001164269.2) was referenced from the National Center for Biotechnology Information. Subsequently, truncated fragments (T1–T3) were designed based on different functional domains. The whole gene synthesis and construction of the pEGFP-C1 vector were outsourced to MDBio, Inc. (New Taipei City, Taiwan). The pEGFP-RAD51-T3 plasmid was further modified by mutating the codons from lysine to alanine at specific positions, resulting in Mut1-Mut4. Details regarding plasmid construction are provided in (Figure S1). After overexpressing the truncated or mutated plasmids in the U87MG cell line for 24 h, the cells were treated with compound **1** for an additional 24 h. Subsequently, cells were harvested, and protein lysate was extracted using 1 × RIPA Lysis Buffer (Cat# 20-188, Merck Millipore). Following protein quantification, 1 μg of protein lysate was combined with 1 μg of GFP antibody and subjected to IP using the Catch and Release Reversible IP System (Cat# 17-500, Merck Millipore) for 12 h. The resulting products from the IP were then subjected to immunoblotting using an antiubiquitination antibody.

### Experimental Animals and Approval by the Ethical Committee

The animal experiments in this study were approved by the Institutional Animal Care and Use Committee (IACUC) of TMU (IACUC approval number: LAC-2021-0359). The mice used for pharmacokinetic and brain distribution studies were 7–8 week-old male C57BL/6 mice. The mice used for orthotopic tumor models were 7–8 week-old male immunodeficient NOD.CB17-Prkdcscid/JNarl (SCID) mice. All mice were purchased from BioLASCO Taiwan Co. Ltd. (Taipei, Taiwan) and housed in the animal facilities at TMU.

### In Vivo Imaging System (IVIS)

Animals were imaged using an IVIS to monitor tumor growth and target expression changes. Initially, animals were anesthetized with isoflurane prior to imaging to minimize movement artifacts. D-luciferin (150 mg/kg) was then administered via an intraperitoneal injection. After 60 s, animals were positioned on the imaging platform, and images were acquired with a 10-s exposure. Experimental data were collected and quantified for fluorescence or bioluminescence signals by using Living Image software in the IVIS system. After each imaging session, animal ID, imaging parameters, and imaging time were recorded for subsequent analysis.

### Computational BBB Penetration Prediction

The data set used to train the deep learning model is obtained from Kaggle (https://www.kaggle.com/datasets/priyanagda/bbbp-smiles/data) [1]. It contains 2050 compounds along with SMILES representations of their molecular structure and binary labels for BBB penetration/nonpenetration. RDKit and Python were used to generate 210 molecular fingerprints from the SMILES representations of these compounds. The data set was split by the proportion of 80:20 as the training and testing set. Initially, all 210 fingerprints were used to train various machine learning models using the classification learner app in Matlab software. However, because some irrelevant fingerprints might interfere with the training results, we used ANOVA to select the top 60 most relevant fingerprints and train the models again with those 60 fingerprints. The classification learning app in Matlab has a total of 34 machine learning models. To select the model with the highest accuracy, all models were trained using the data set we generated with 60 fingerprints and a 5-fold cross-validation. Among all 34 models, the K-nearest neighbors algorithm has both the highest validation accuracy rate of 0.88 and the highest test accuracy rate of 0.92; therefore, we used this model to make further predictions.

### UPLC-MS Analysis Method

#### Chemical Reagents and Animals

Our laboratory-synthesized compound **1**, ammonium acetate, was purchased from Acros (Massachusetts, U.S.) and formic acid from Thermo Fischer (Massachusetts, U.S.). LC-grade acetonitrile (>99%) was purchased from Honeywell (Michigan, U.S.), and LC-grade DMSO (>99%) was purchased from Merck (Darmstadt, Germany). All of the solvents were filtered with a 0.22 μm filter before use.

#### Instrument and Analytical Conditions

The liquid chromatography was performed on an Acquity H class UPLC system (Waters Corp., Massachusetts, U.S.) equipped with an ACQUITY Premier PDA eλ detector (Waters Corp., Massachusetts, U.S.) with an autosampler temperature of 15 °C. Separation was performed at 25 °C on a Waters Acquity HSS T3 C_18_ column (2.1 mm × 100 mm, 1.8 μm particle size) with a gradient elution using A: water (containing 2 mM ammonium acetate and 0.1% formic acid) and B: acetonitrile as the mobile phase. The gradient program is as follows: 96% A (initial), 96% A (1 min), 65% A (7 min), 65% A (7.5 min), 96% A (8 min), and 96% A (12 min). The flow rate was set at 0.5 mL/min, and the injection volume was 3 μL. The UV wavelength was adjusted to 254 nm for compound **1** detection.

#### Stock Solution

A stock solution of compound **1** (1 mg/mL) was prepared in DMSO and then diluted with DMSO to give a series of standard solutions. The stock solution for the validation of brain matrices was directly prepared with the brain extraction in an identical concentration.

#### Extraction of Plasma and Brain Tissue Samples

Following anesthesia of mice with isoflurane, blood samples were collected via a cardiac puncture. The blood was centrifuged at 2000*g* for 20 min at 4 °C to obtain plasma. Plasma samples were then mixed with an equal volume of 1 M zinc sulfate heptahydrate solution (cat. no. 221376, Sigma-Aldrich) and allowed to stand for 30 min. Subsequently, the mixture was centrifuged at 12,000*g* for 30 min at 4 °C, and the supernatant was collected for analysis. Brain tissues were homogenized using a Tenbroeck tissue grinder at a ratio of 5 μL of DMSO per mg of tissue. The homogenates were then centrifuged at 12,000*g* for 30 min at 4 °C, and the supernatant was collected for analysis.

#### Storage Condition of Biological Matrices

The biological matrices, including brain extraction and plasma, were stored at −80 °C until use. All of the matrices were used for validation of the method after appropriate homogenization.

#### Standard and Quality Control Sample

The preparation of the standard compound **1** sample was directly diluted from the stock solution with DMSO into seven different concentrations ranging from 10,000 to 125 ng/mL and four concentrations were selected (10,000, 1000, 500, and 125 ng/mL) as QC samples. For the matrix-spiked samples, the stock solution of compound **1** in brain extraction was diluted with brain extraction, whereas plasma samples were prepared with the addition of standard compound **1** stock solution and diluted with plasma. Both matrix-spiked standard samples were prepared at seven different concentrations ranging from 10,000 ng/mL to 125 ng/mL, and four concentrations were selected (10,000, 1000, 500, and 125 ng/mL) as QC samples. All of the injecting samples were filtered with a 0.22 μm filter before use.

#### Pharmacokinetics and Brain Distribution Study

Compound **1** was prepared in DMSO at a concentration of 180 mg/mL, and in TMZ at 30 mg/mL. C57BL/6 mice were intraperitoneally injected with compound **1** at 360 mg/kg, TMZ at 60 mg/mL, or DMSO at 2 μL/g as the control group. Mice were sacrificed by intraperitoneal injection after 30 min, and plasma as well as brain tissue extracts were collected following the aforementioned procedures.

#### Validation of the UPLC Method

The analysis method was validated according to the request by US FDA for bioanalytical method validation and guidance for the industry (FDA, 2018). The validation of specificity, linearity, LLOD, LLOQ, precision, accuracy, and ME were assessed.

#### Specificity

The evaluation specificity was assessed with the injection of each matrix and standard compound **1** solution to analyze the possible interference in the corresponding composition.

#### Linearity of Calibration Curves and Lower Limits of Quantification

The linearity of each matrix was tested against ANOVA. Three calibration curves were constructed by plotting the area versus theoretical concentration to obtain the linear equation and correlation coefficient (*R*^2^) for the evaluation through linear regression analysis.

The LLOD was defined as the amount of compound **1** that could be detected with a signal-to-noise ratio of 3. The LLOQ was considered as the lowest drug concentration that could be quantified with a relative precision and accuracy of ≤20%.

#### Precision and Accuracy

Intraday accuracy and precision were established through the performance of four different QC samples, as described with at least five replicates. Interday accuracy and precision were established through the performance in three consecutive days. The accuracies were evaluated by the deviation of the mean concentration of the measured samples versus the theoretical concentration and expressed as a percentage of bias. The precisions were evaluated from the relative standard deviation of the measured samples and expressed as the percentage of the coefficient of variation from the mean concentration of all replicates. The intraday and interday accuracies and precisions for QC concentrations should be less than or equal to ±15% or ±20% if LLOQ.

#### Extraction Recovery

The extraction recovery of compound **1** from mice brain and plasma was expressed as a percentage, determined by the comparison of the theoretical concentration added to each matrix and the experimental result obtained after the extraction procedure. All of the extraction recovery assays were performed in three different concentrations with at least duplicates in run.

#### Matrix Effect

The assessment of ME was determined by the comparison of the slopes of each calibration curves for each matrix toward compound **1** standard curve with the following equation
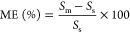
where *S*_m_ is the slope of the calibration curve of compound **1** in the corresponding matrix and *S*_s_ represents the slope of compound **1** standard calibration curve.

#### Drug Inhibition of the Orthotopic Tumor Growth Experiment

For orthotopic inoculation, CT-2A cells (1 × 10^5^) or CT-2A-R (2 × 10^5^) were suspended in 4 μL of DMEM and implanted into the brain of B6 mice. PT#3-R cells (5 × 10^5^) were suspended in 4 μL of DMEM and implanted into the brain of SCID mice. After cell implantation, mice were monitored daily for survival, and their body weight was measured every 3 days starting from the seventh day. The oral vehicle consisted of 40% ethyl oleate, 12% lecithin, 20% propylene glycol, and 28% Triton X-100. Mice were administered 5 μL of vehicle per gram of body weight along with 50 mg/kg TMZ or a combination of TMZ and compound **1**. Intraperitoneal injections were administered with DMSO (solvent control), TMZ (10 μg/kg), TMZ (10 μg/kg), and compound **1** (10 or 20 μg/kg). Upon the natural death of the mice, brains were extracted and entrusted to BioTools for sectioning and HE staining.

### Pharmacokinetics Study

The pharmacokinetic study protocol of compound **1** was approved by the IACUC of TMU (LAC-2022-0452), in compliance with National Research Council’s Guide for the Care and Use of Laboratory Animal. 8 Week-old male Sprague–Dawley rats were provided by BioLASCO (Taipei, Taiwan). Each rat (*n* = 3) was intravenously administered with a single dosage of 10 mg/kg A089 through the jugular vein. Blood samples were collected from the jugular vein at predetermined time points: 0.03, 0.08, 0.25, 0.5, 1, 2, 4, 6, 8, 12, and 24 h postadministration. The blood samples were centrifuged at 4 °C to obtain the plasma samples. For protein precipitation, an aliquot of acetonitrile was added to the plasma, followed by centrifugation and filtration using a 0.22 μm filter. The filtrate was then analyzed by ultraperformance liquid chromatography–tandem mass spectrometry (UPLC–MS/MS, TQ-XS, Waters Corp., Manchester, UK). Pharmacokinetic parameters were calculated by using a noncompartmental analysis with Phoenix WinNonlin software (Certara, Princeton, NJ, USA).

### Statistical Analyses

Statistical analyses of two groups of data from Western blotting, micronucleus assay, HR/NHEJ DNA repair assays, MTT, CCK8 assay, tumor weight of subcutaneously inoculated mice, RNA-seq data of the TCGA-GBM data set, qPCR, etc., were carried out using Student’s *t*-test with a two-tailed distribution. Multiple groups of data from the MTT assay and Western blotting were analyzed by two-way analysis of variance with subsequent Tukey’s multiple comparison test. The Gehan–Breslow–Wilcoxon test was used to compare the survival curves (Kaplan–Meier curve) of orthotopic GBM mice. Quantitative data (bar chart) are shown as mean ± SEM. A value of *p* < 0.05 was considered statistically significant (*p < 0.05; **p < 0.01; and ***p < 0.001).

## Supporting Information Available

Crystallographic data (CSV)
tPSA, partition coefficient (*P*) (log *P*), and molecular weight of approved drugs in relation to BBB penetration efficiency; nuclear magnetic resonance spectra 1H and 13C; HPLC purity analysis; inhibitory effects of compounds 1–10 on A172R and PT#3R TMZ-resistant cell line; schematic diagram of RAD51 amino acid sequence truncation and mutagenesis design; computational AI BBB penetration prediction; bioanalysis method validation; body weight changes in mice administered with vehicle, TMZ, and compound **1** in vivo; and reference (PDF)
