# Supplementary material for: Repurposing Linezolid in Conjunction with Histone Deacetylase Inhibitor Access in the Realm of Glioblastoma Therapies
Source: J Med Chem. 2025 Jan 21;68(3):2779–803. doi: 10.1021/acs.jmedchem.4c02086 (PMC11831592; doi:10.1021/acs.jmedchem.4c02086)
Supplement: Supplementary file 2 — jm4c02086_si_002.pdf [file jm4c02086_si_002.pdf]

Supporting Information

Repurposing Linezolid in Conjunction with a Histone Deacetylase Inhibitor Access in the Realm of Glioblastoma Therapies

I-Chung Chen[a#], Hong-Yi Lin[b,c#], Zheng-Yang Liu[a], Wen-Bin Yang[d,e,f], Hoang Yen Tran[a,g], Mei-Jung Lai[h], Chung-Han Wang[d], Tzu-Yuan Kao[d], Chia-Yang Hung[i], Tsung-I Hsu[d,e,f,h,j,k*] and Jing-Ping Liou[a,j,k*]

[#]Contributed equally to this work.

[a] School of Pharmacy, College of Pharmacy, Taipei Medical University, Taipei, Taiwan

[b] Taipei Neuroscience Institute, New Taipei City, Taiwan

[c] Graduate Institute of Medical Sciences, College of Medicine, Taipei Medical University Taipei, Taiwan

[d] Ph.D. Program in Medical Neuroscience, College of Medical Science and Technology, Taipei Medical University and National Health Research Institutes, Taipei, Taiwan

[e] International Master Program in Medical Neuroscience, College of Medical Science and Technology, Taipei Medical University Taipei, Taiwan

[f] TMU Research Center of Neuroscience, Taipei Medical University Taipei, Taiwan

[g] Department of Pharmacology and Clinical Pharmacy, Faculty of Pharmacy, Can Tho University of Medicine and Pharmacy, Vietnam

[h] TMU Research Center for Drug Discovery, Taipei Medical University, Taipei, Taiwan

[i] Department of Immuno-Oncology, Beckman Research Institute, City of Hope, Duarte, CA, USA 91010

[j] Ph.D. Program in Drug Discovery and Development Industry, College of Pharmacy, International Master Program in Medical Neuroscience, College of Medical Science and Technology, Taipei Medical University, Taipei, Taiwan

[k] TMU Research Center of Cancer Translational Medicine, Taipei Medical University, Taipei, Taiwan

Corresponding authors

Tsung-I Hsu - Ph.D. Program in Medical Neuroscience, College of Medical Science and Technology, Taipei Medical University and National Health Research Institutes, Taipei 110, Taiwan

TMU Research Center of Neuroscience, Taipei Medical University, Taipei 110, Taiwan

International Master Program in Medical Neuroscience, College of Medical Science and Technology, Taipei Medical University, Taipei 110, Taiwan

TMU Research Center of Cancer Translational Medicine, Taipei, 110 Taiwan

E-mail - dabiemhsu@tmu.edu.tw

Jing Ping Liou - School of Pharmacy, College of Pharmacy, Taipei Medical University, Taipei 110, Taiwan

TMU Research Center for Drug Discovery, Taipei Medical University, Taipei 110, Taiwan.

Ph.D. Program in Drug Discovery and Development Industry, College of Pharmacy, Taipei Medical University, Taipei 110, Taiwan.

E-mail - jpl@tmu.edu.tw

Contents:

Supplementary information I. The topological polar surface area (tPSA), partition coefficient (P) (logP), and molecular weight (M.W.) of approved drugs in relation to blood-brain barrier (BBB) penetration efficiency. 3

Supplementary information II. Nuclear magnetic resonance spectra 1H & 13C. 4

Supplementary information III. HPLC purity analysis. 32

Supplementary information IV. Inhibitory effects of compound 1-10 on A172R and PT#3R TMZ-resistant cell line. 46

Supplementary information V. Schematic diagram of RAD51 amino acid sequence truncation and mutagenesis design. 48

Supplementary information VI. Computational AI BBB penetration prediction. 49

Supplementary information VII. Bioanalysis method validation. 50

Supplementary information VIII. Body weight changes in mice administered with vehicle, TMZ, and Compound 1 *in vivo*. 52

Supplementary information IX. Supporting Reference 0

Supplementary information I. The topological polar surface area (tPSA), partition coefficient (P) (logP), and molecular weight (M.W.) of approved drugs in relation to blood-brain barrier (BBB) penetration efficiency.

	TMZ	Linezolid	Vorinostat	Panobinostat	Ganetespib
logP	-0.33	0.58	1.79	2.9	3.33
tPSA (Å^2)	103.72	71.11	78.43	73.39	88.4
M.W. (Da)	194.15	337.35	264.33	349.43	364.41
BBB penetration	~20 %[a]	~70 %[b]	<10 %[c]	<5 %[d]	N/A

	1	2	3	4	5	6	7	8
logP	2.31	1.43	1.97	2.9	0.61	2.1	2.64	2.93
tPSA (Å^2)	103.37	120.44	103.37	109.16	137.51	98.8	91.34	111.57
M.W. (Da)	470.50	466.51	444.46	519.58	520.53	489.50	449.86	473.50

	9	10	11	12	13	14
logP	3.17	3.5	0.34	1.22	1.81	0.88
tPSA (Å^2)	102.78	102.78	149.54	132.47	138.26	132.47
M.W. (Da)	487.53	501.56	523.56	527.55	576.63	501.52

a. *Clin Cancer Res*. **2004**, 10, 3728–36[1]
b. *Le Infezioni in Medicina*, **2005**, n. 2, 112-119,[2]
c. *PLoS One*. **2013**, 26, 8(7), e69964.[3]
d. *J Pharmacol Exp Ther*. **2023**, 387(3), 315-327.[4]

Supplementary information II. Nuclear magnetic resonance spectra 1H & 13C.

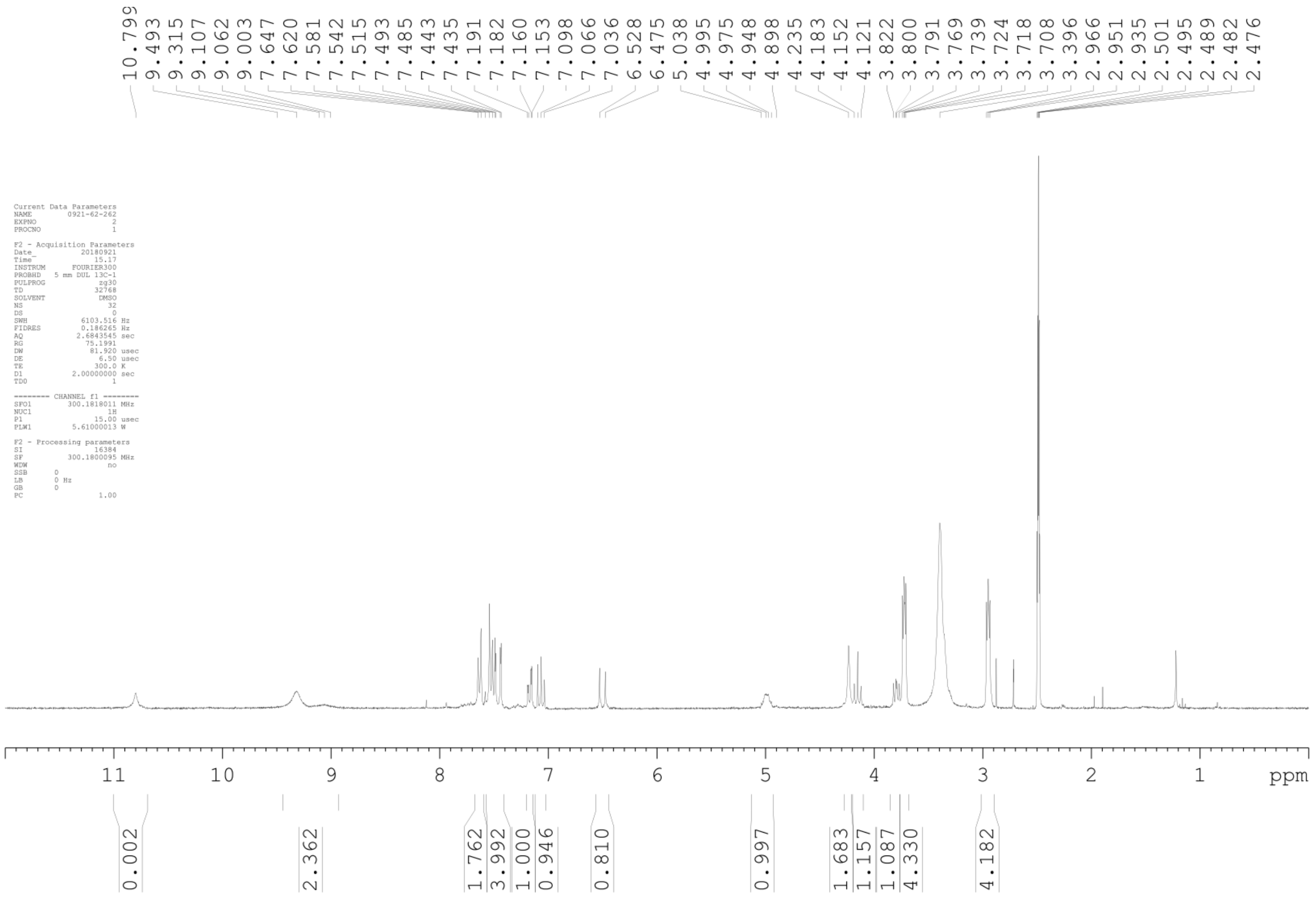

S-II-1. ^{1}H NMR spectrum of compound **1**

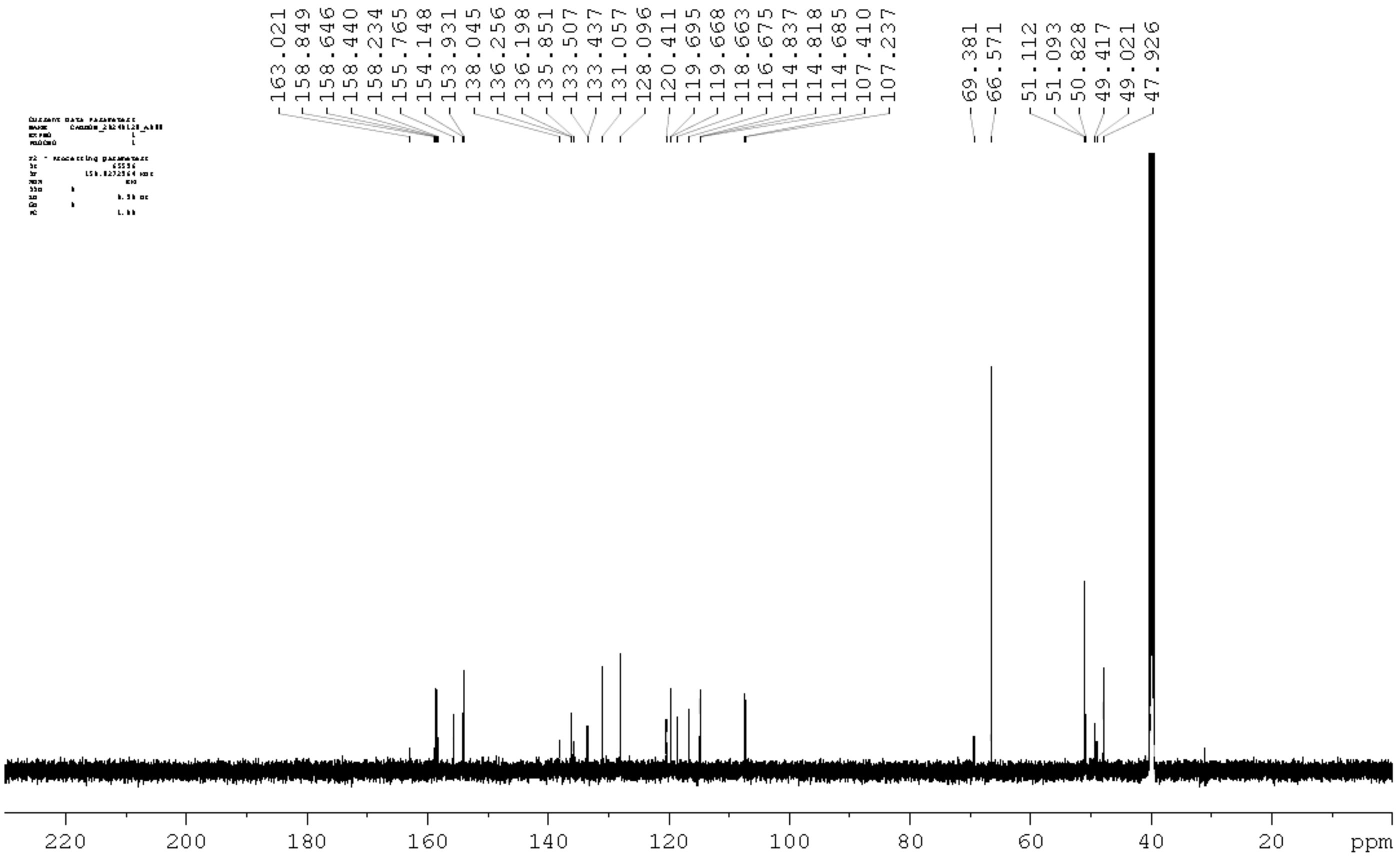

S-II-2. ^{13}C NMR spectrum of compound **1**

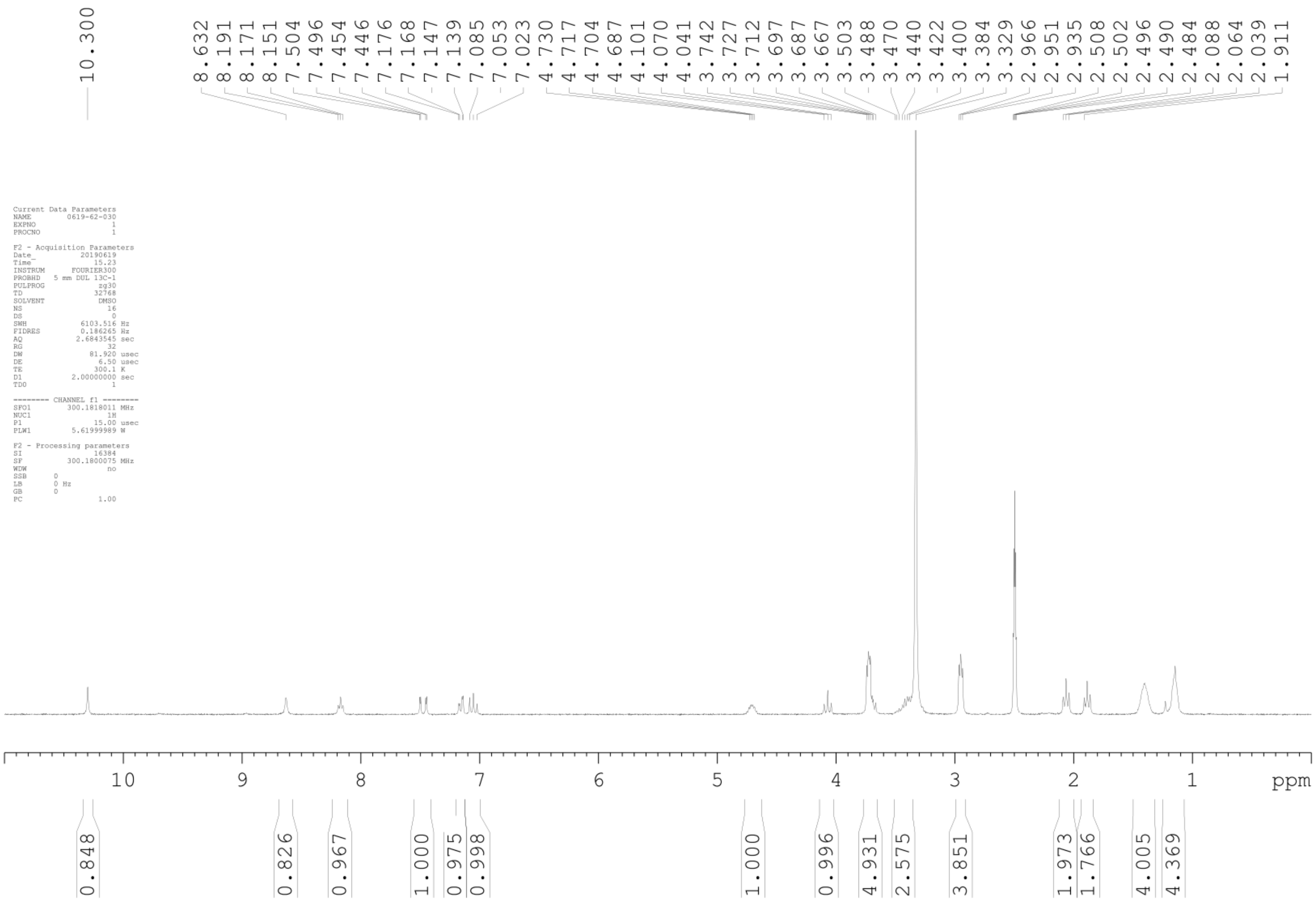

S-II-3. ^{1}H NMR spectrum of compound **2**

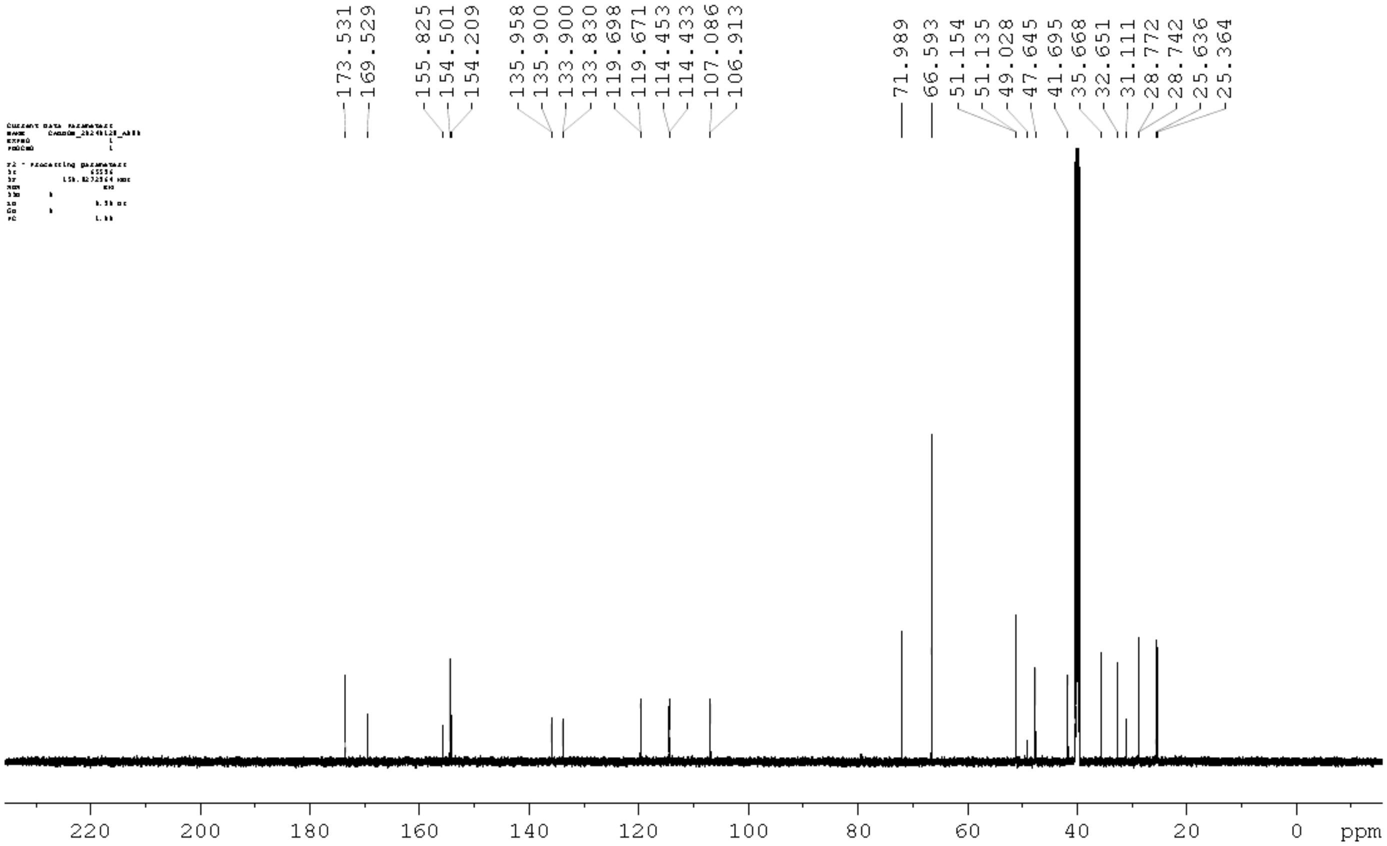

S-II-4. ^{13}C NMR spectrum of compound **2**

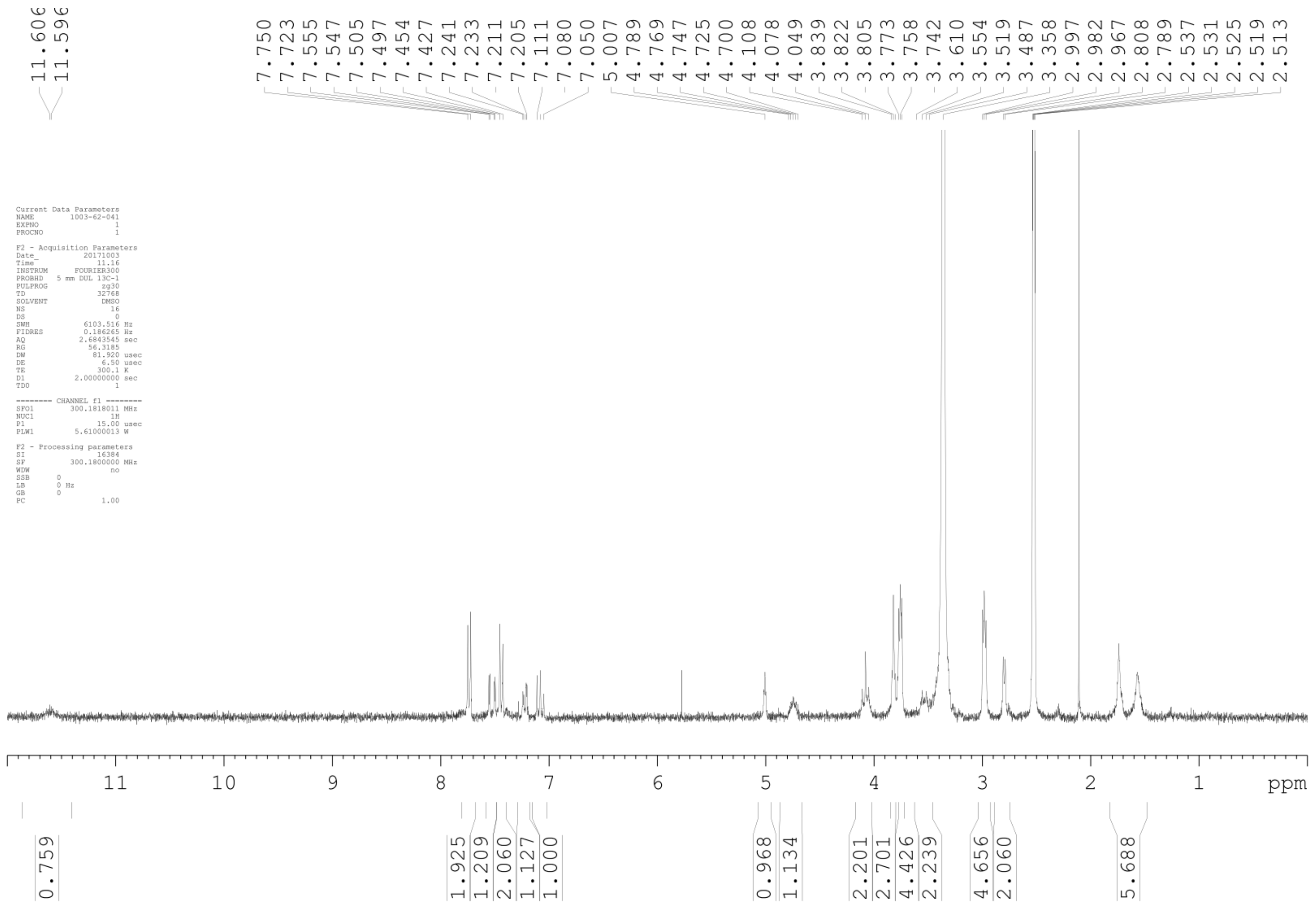

S-II-5. ^{1}H NMR spectrum of compound **3**

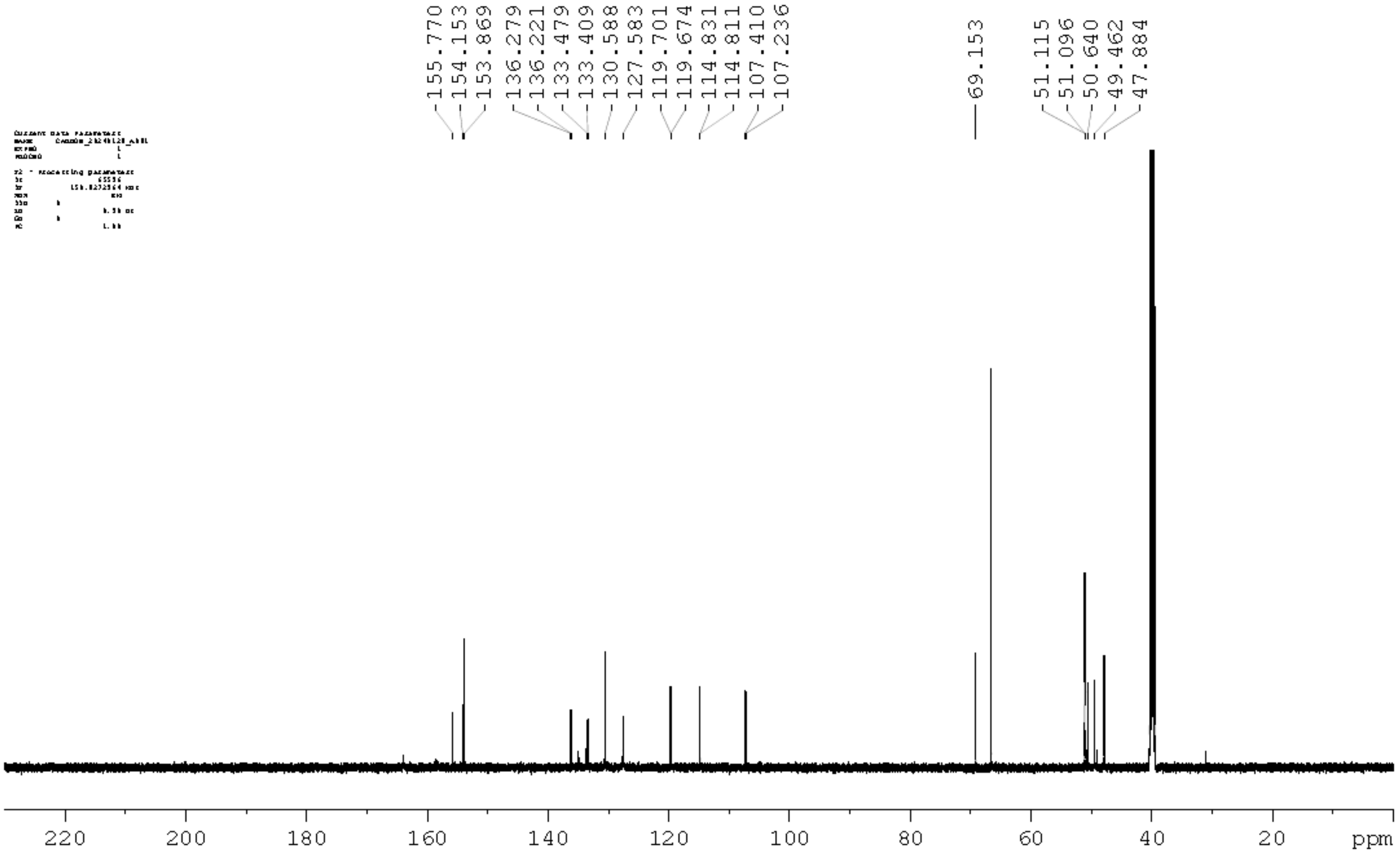

S-II-6. ^{13}C NMR spectrum of compound **3**

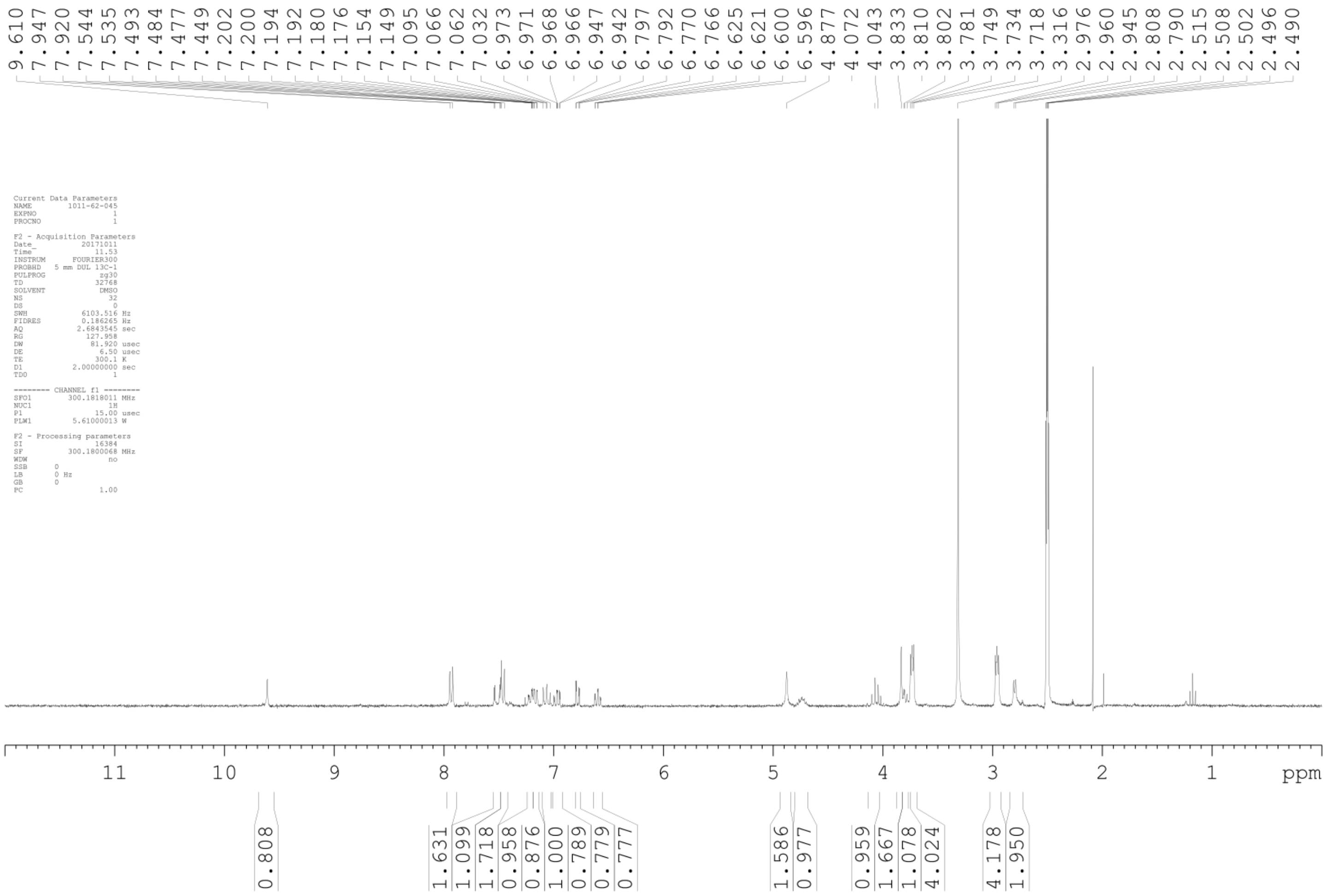

S-II-7. ^{1}H NMR spectrum of compound **4**

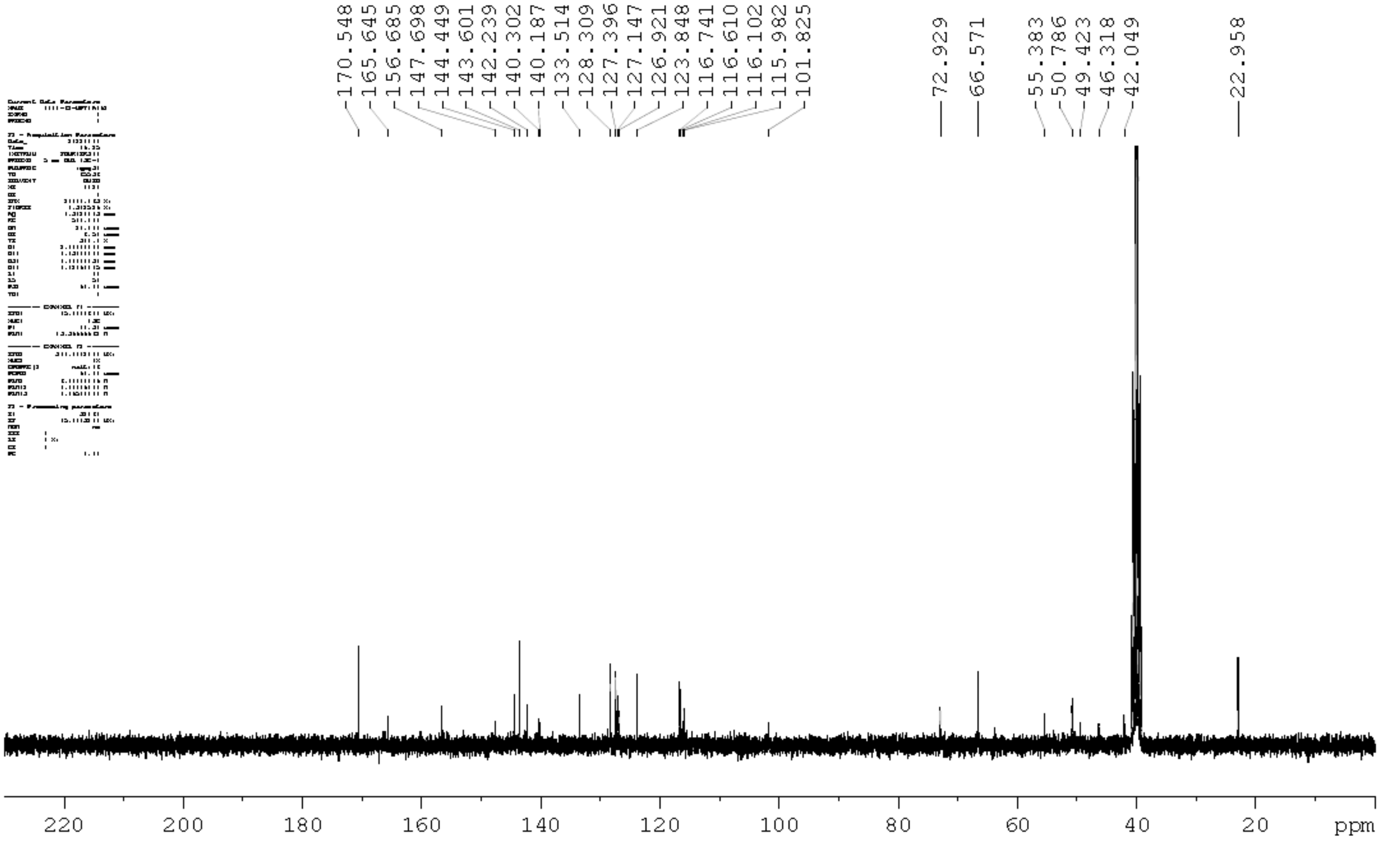

S-II-8. ^{13}C NMR spectrum of compound **4**

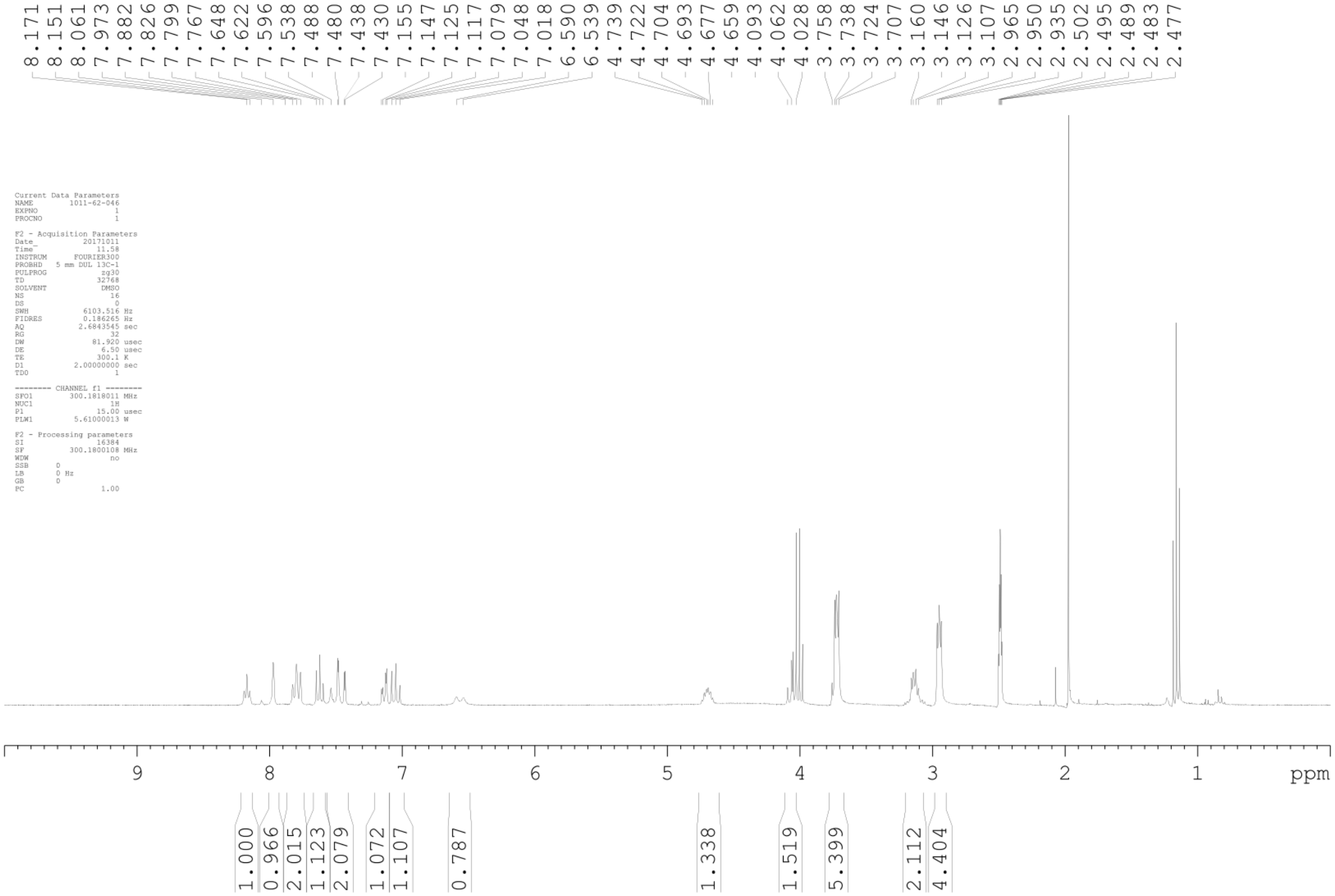

S-II-9. ^{1}H NMR spectrum of compound **5**

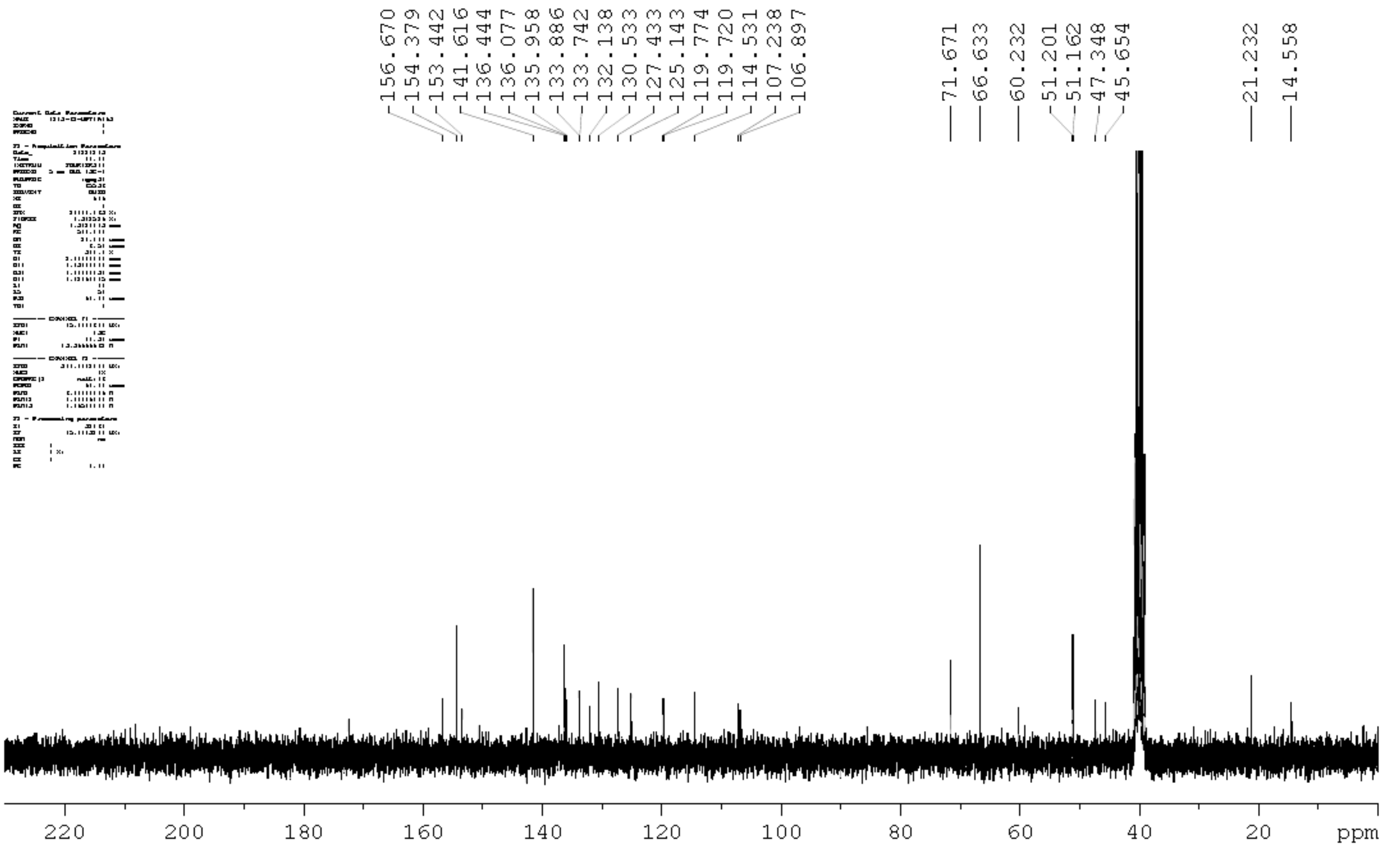

S-II-10. ^{13}C NMR spectrum of compound **5**

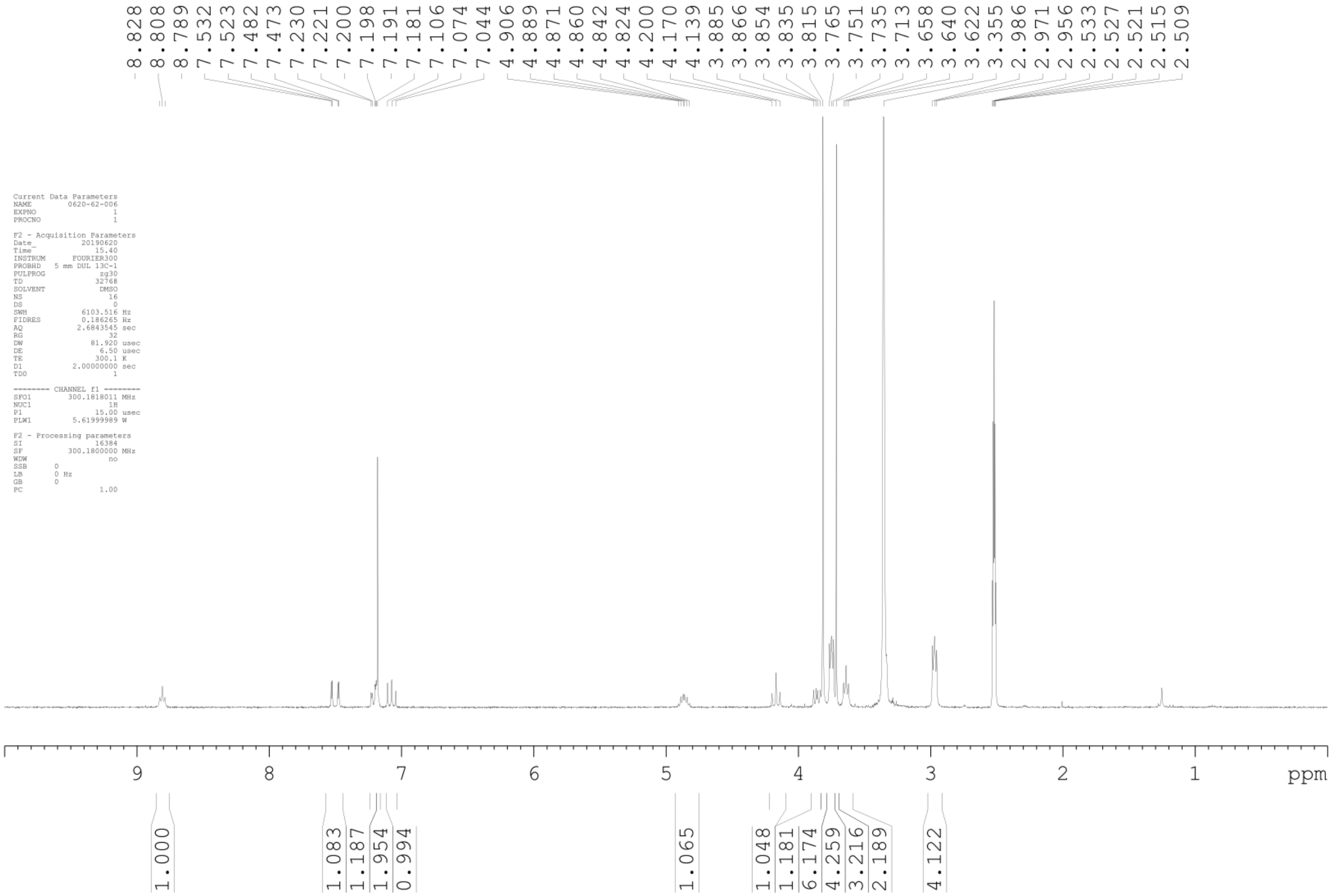

S-II-11. ^{1}H NMR spectrum of compound **6**

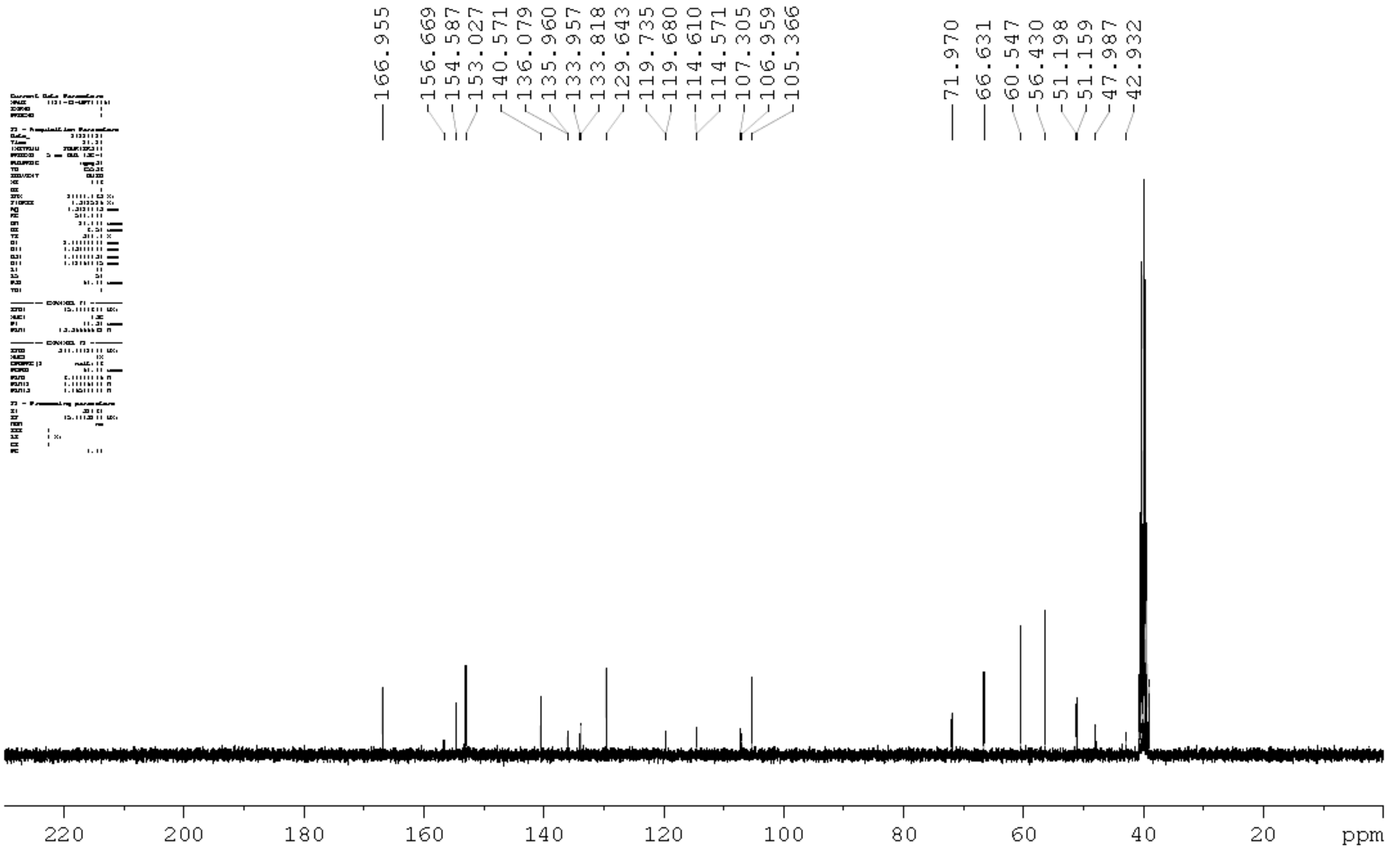

S-II-12. ^{13}C NMR spectrum of compound **6**

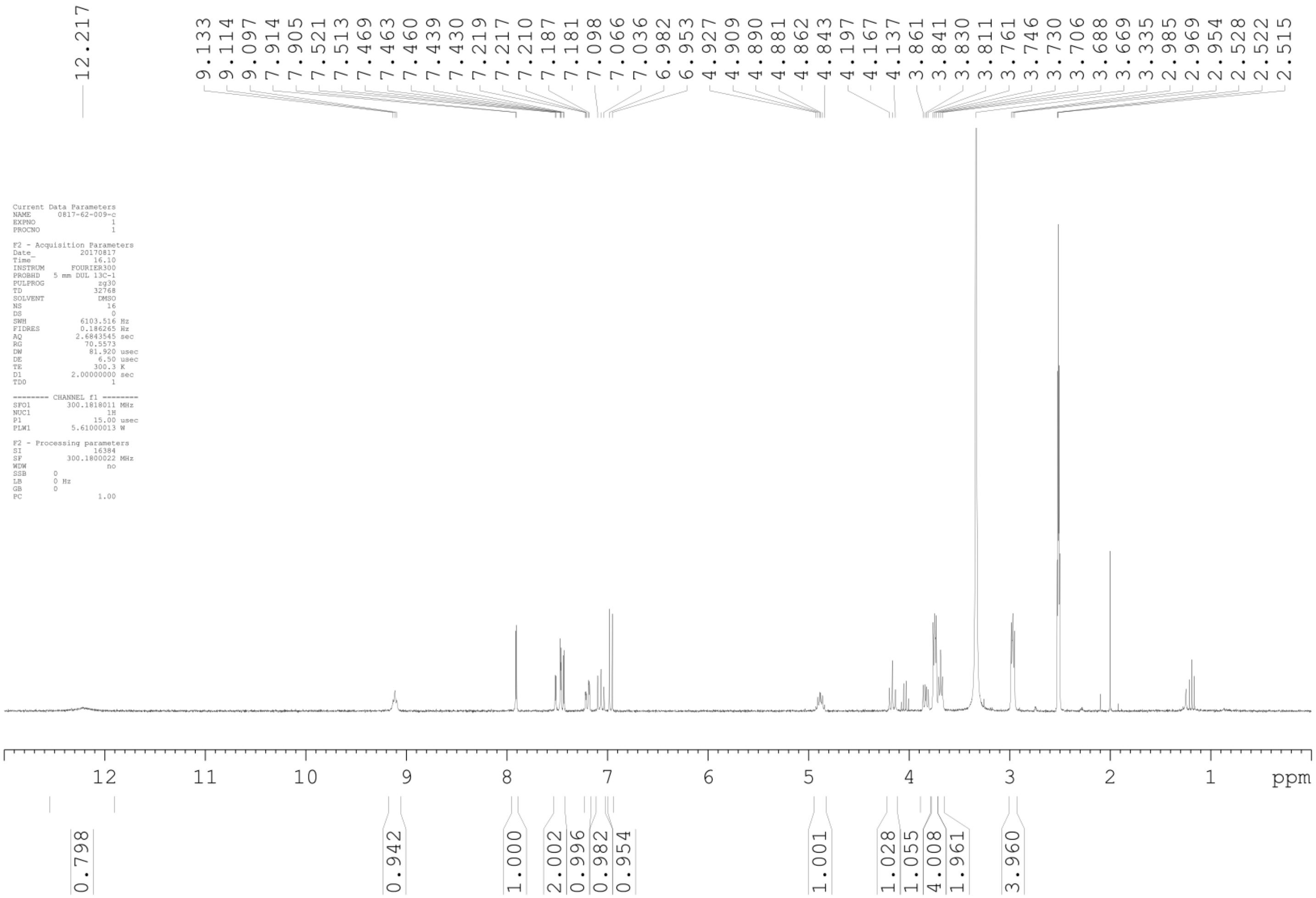

S-II-13. ^{1}H NMR spectrum of compound **7**

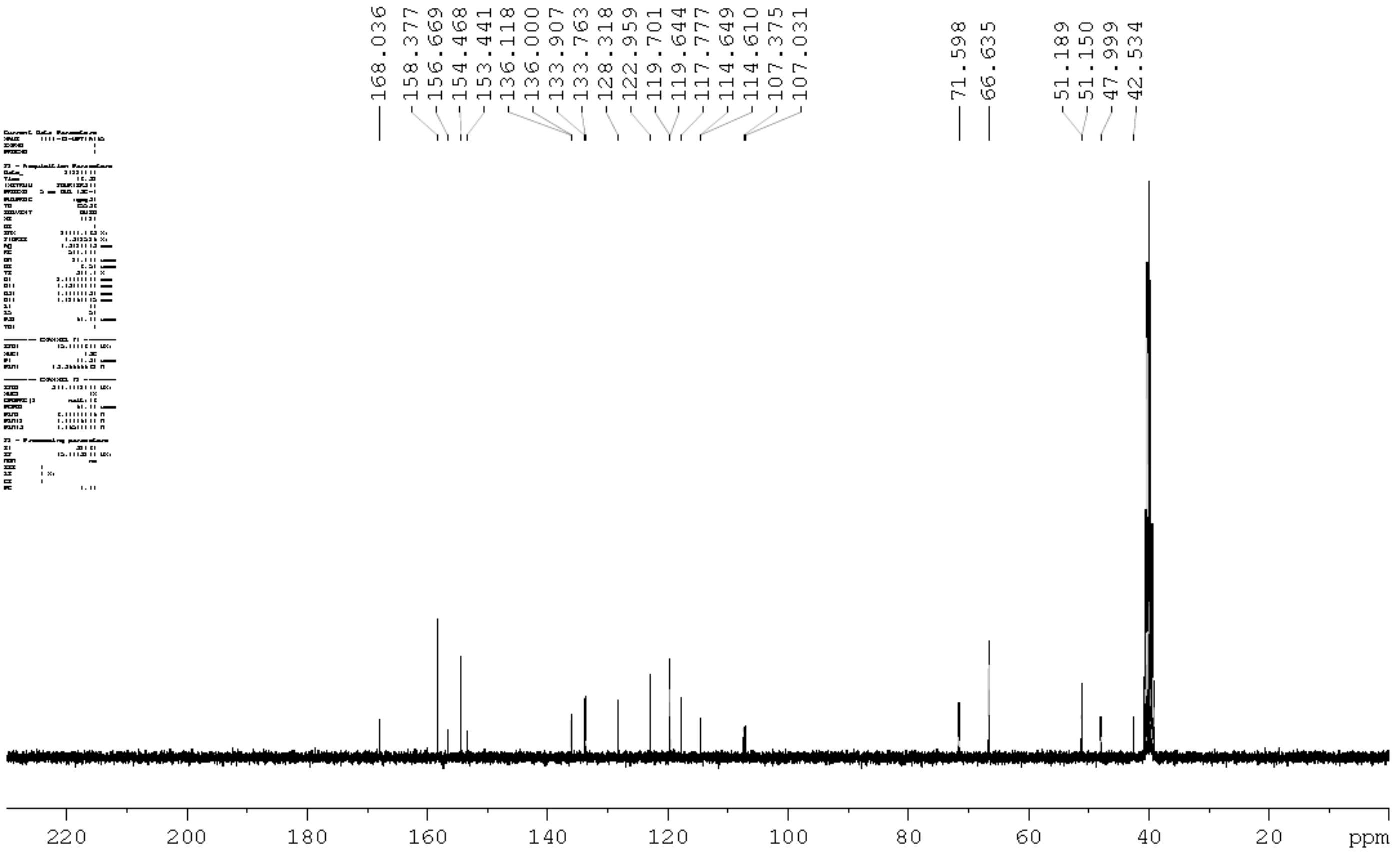

S-II-14. ^{13}C NMR spectrum of compound **7**

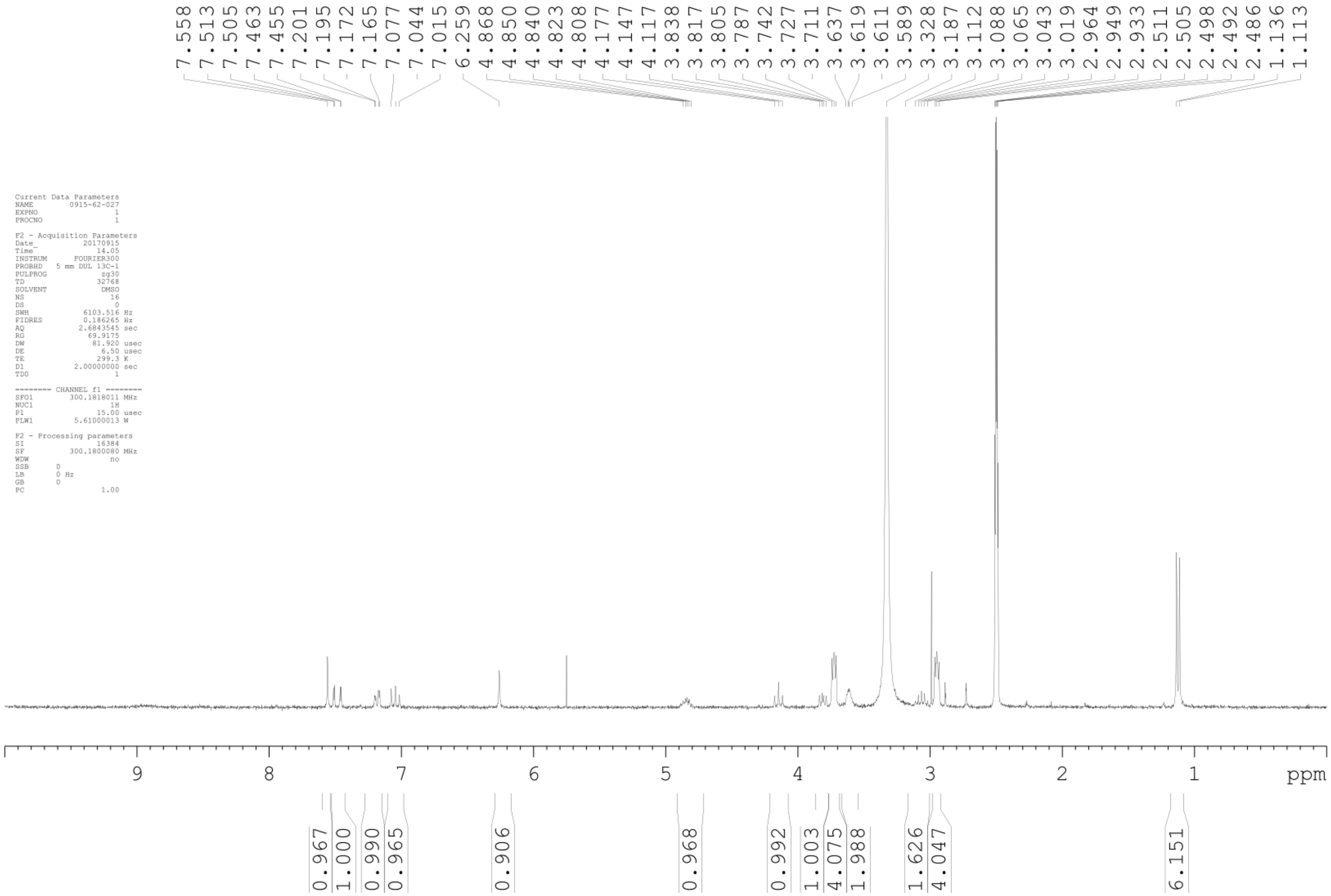

S-II-15. ^{1}H NMR spectrum of compound **8**

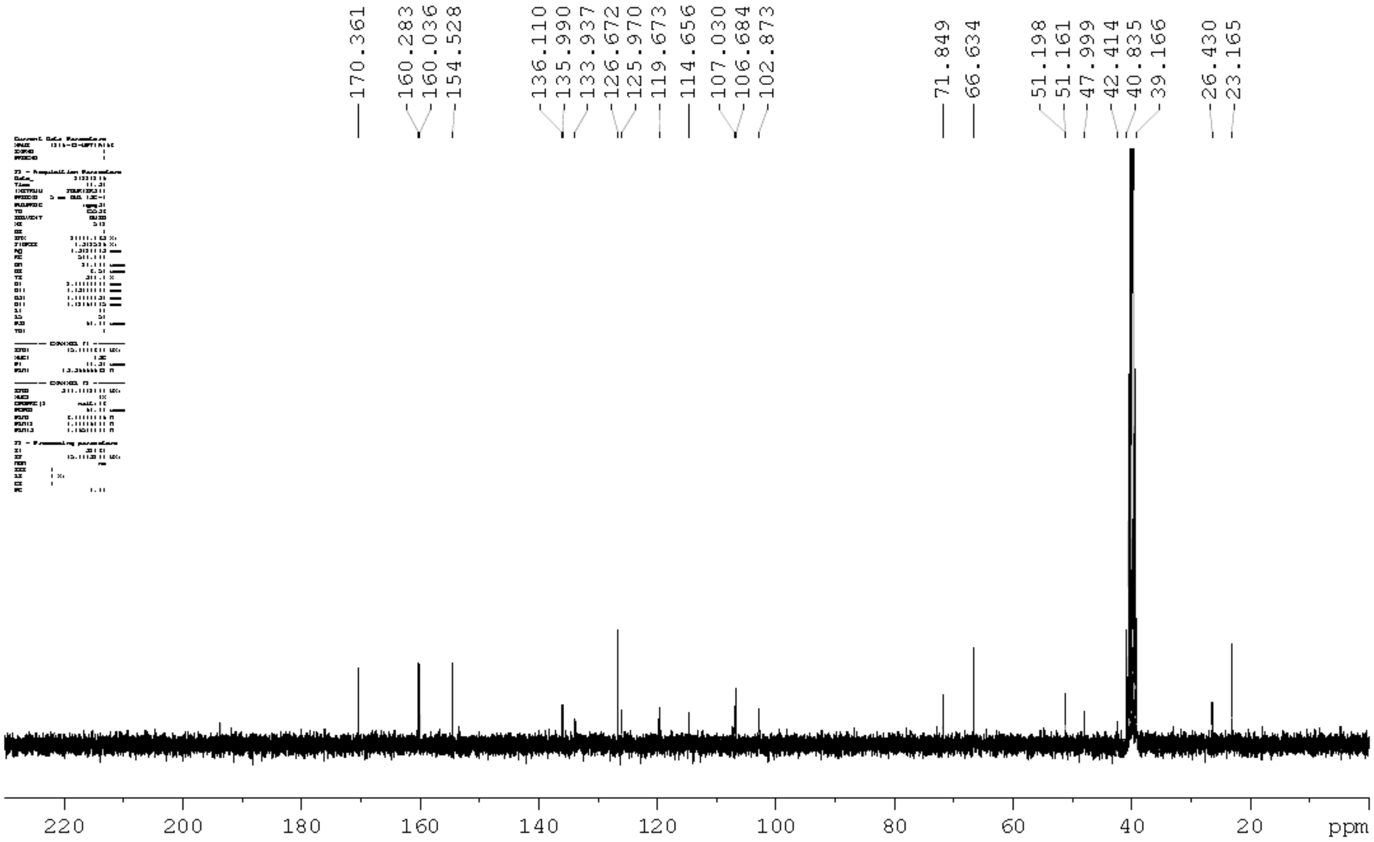

S-II-16. ^{13}C NMR spectrum of compound **8**

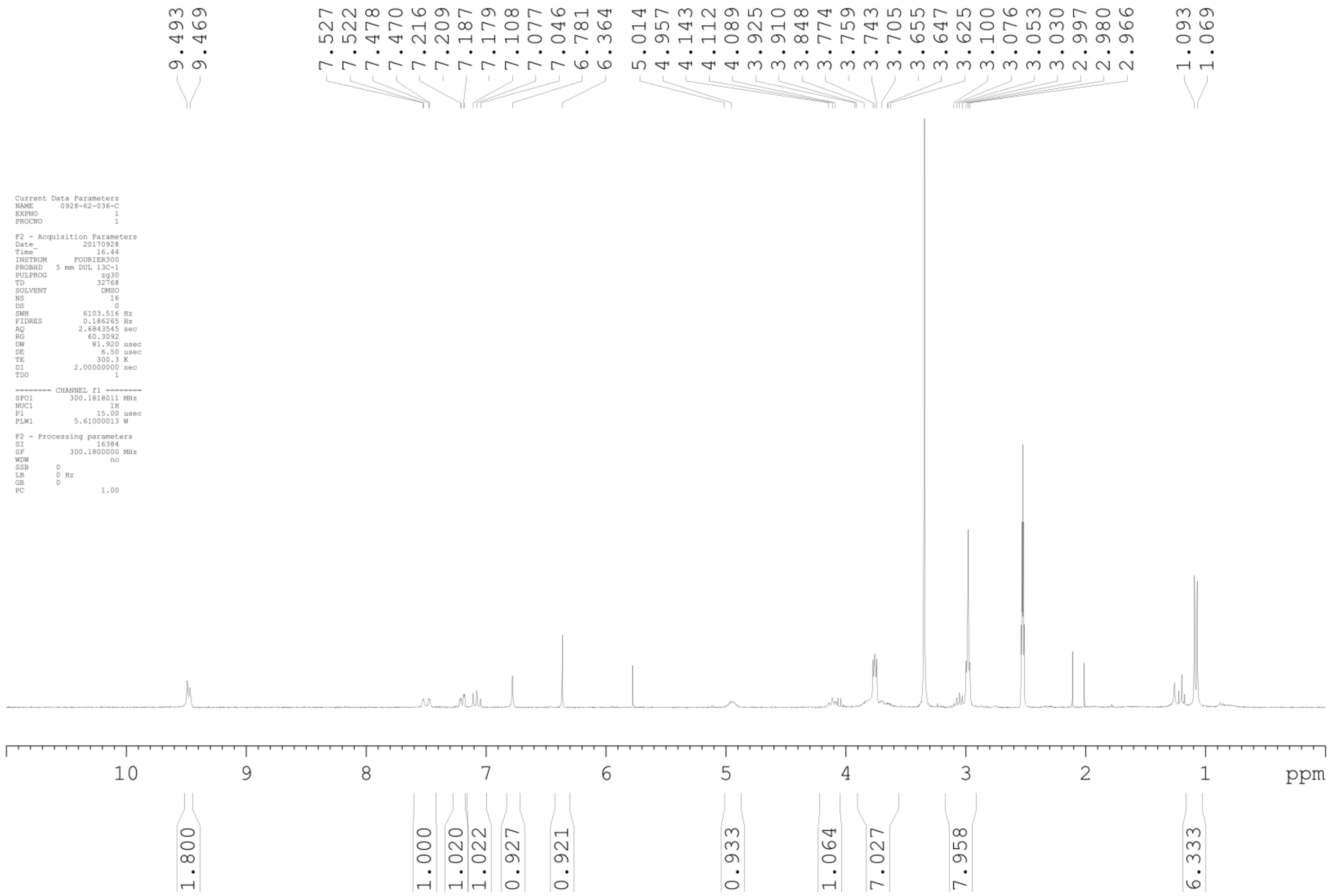

S-II-17. ^{1}H NMR spectrum of compound **9**

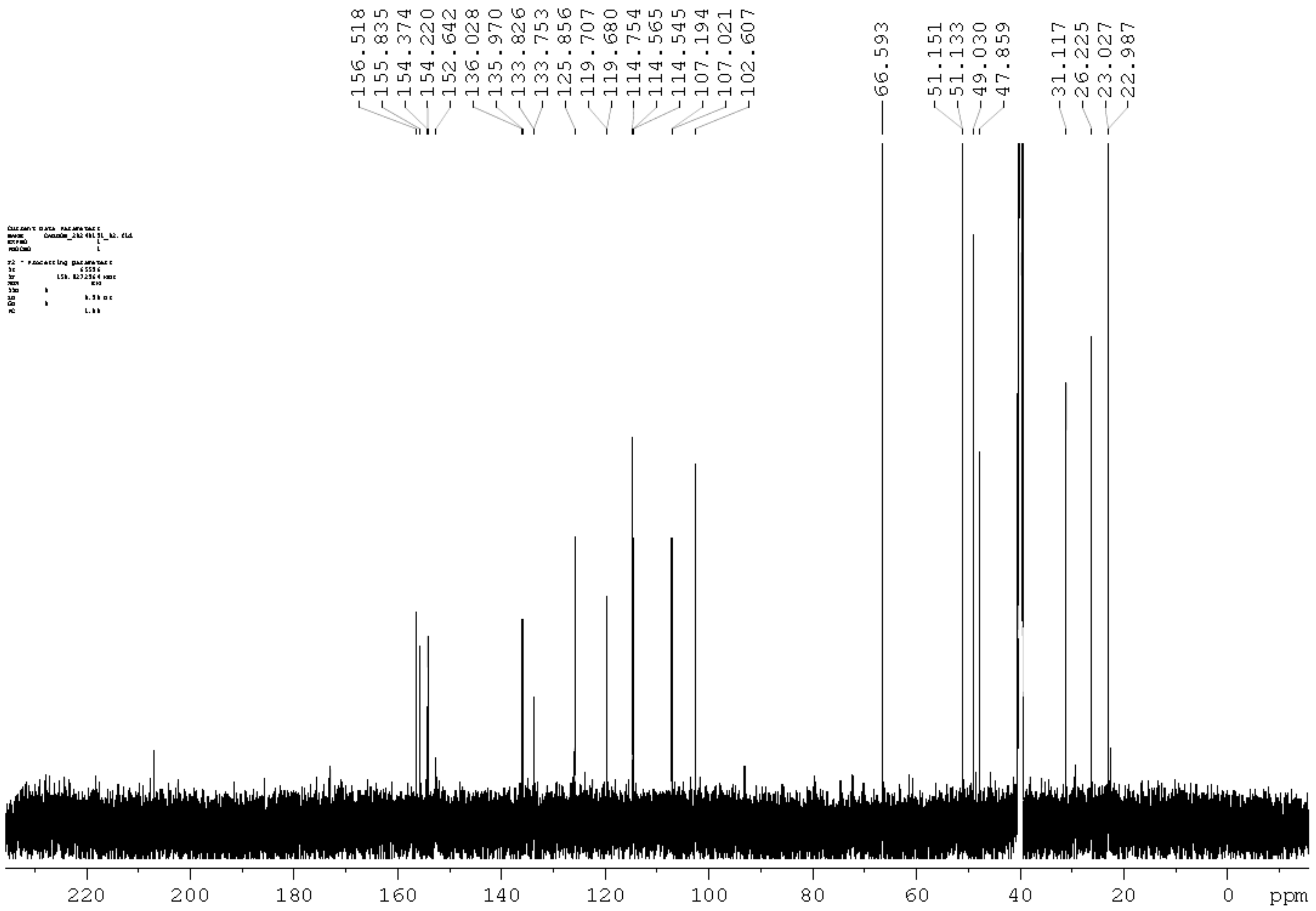

S-II-18. ^{13}C NMR spectrum of compound **9**

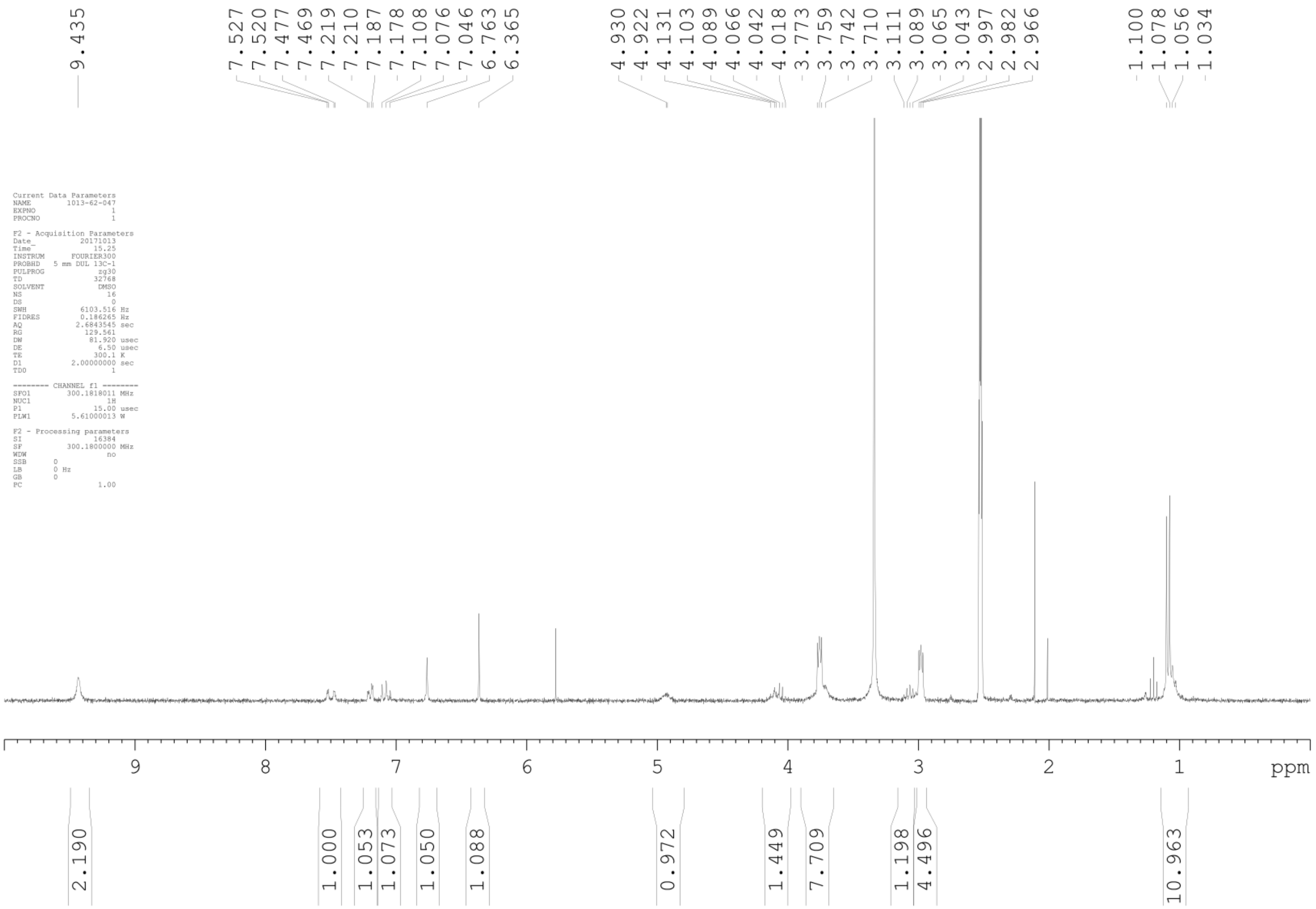

S-II-19. ^{1}H NMR spectrum of compound **10**

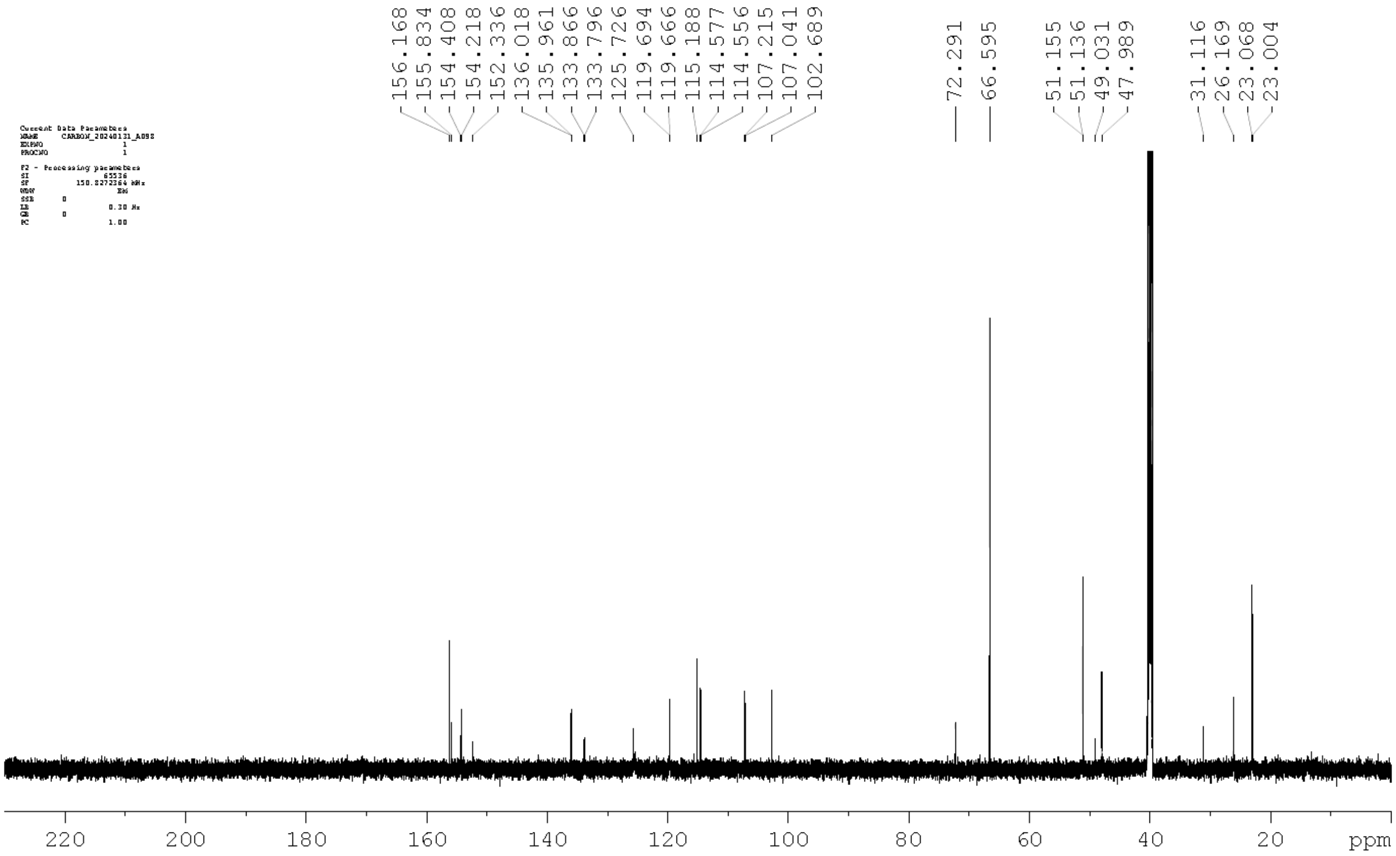

S-II-20. ^{1}H NMR spectrum of compound **10**

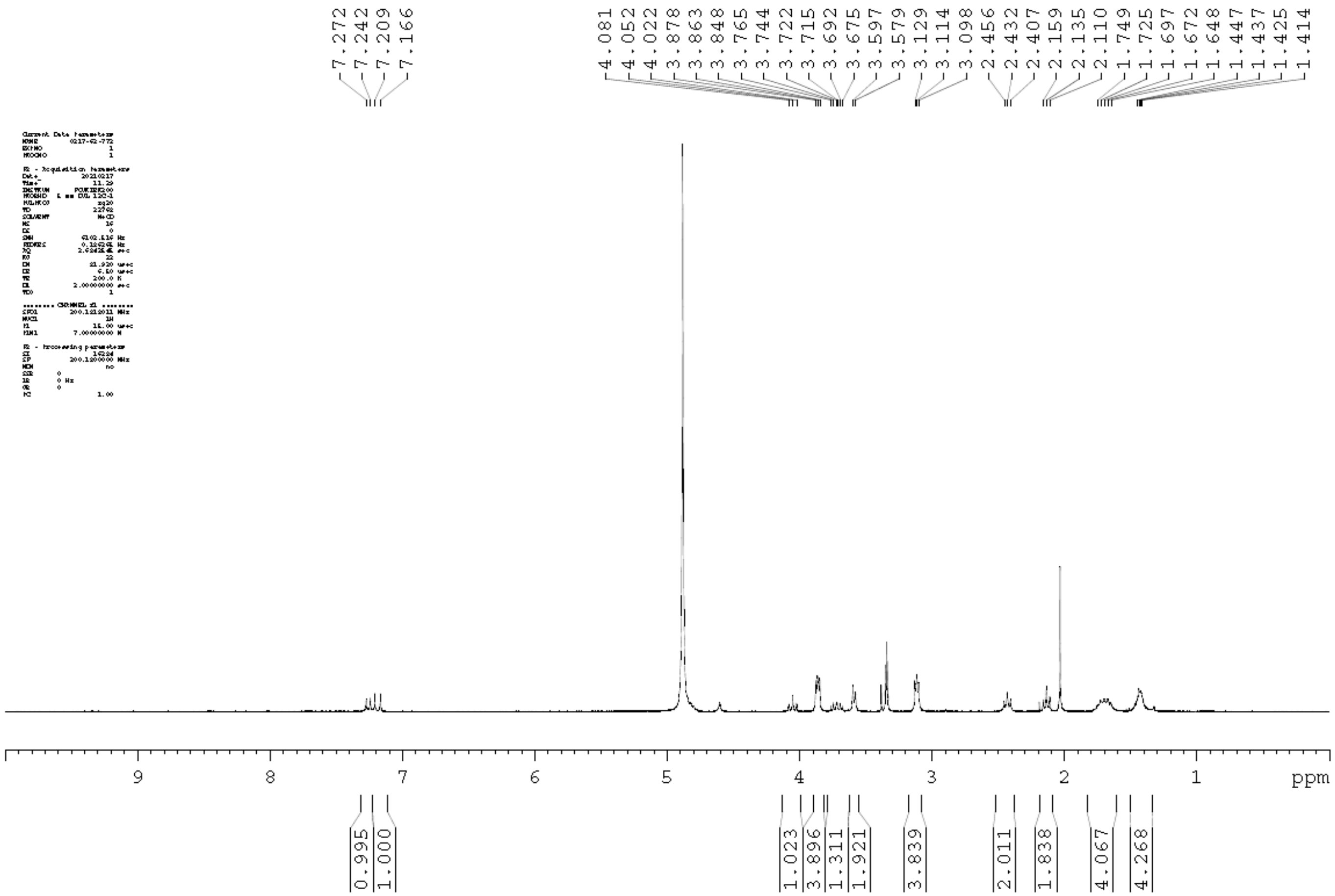

S-II-21. ^{1}H NMR spectrum of compound **11**

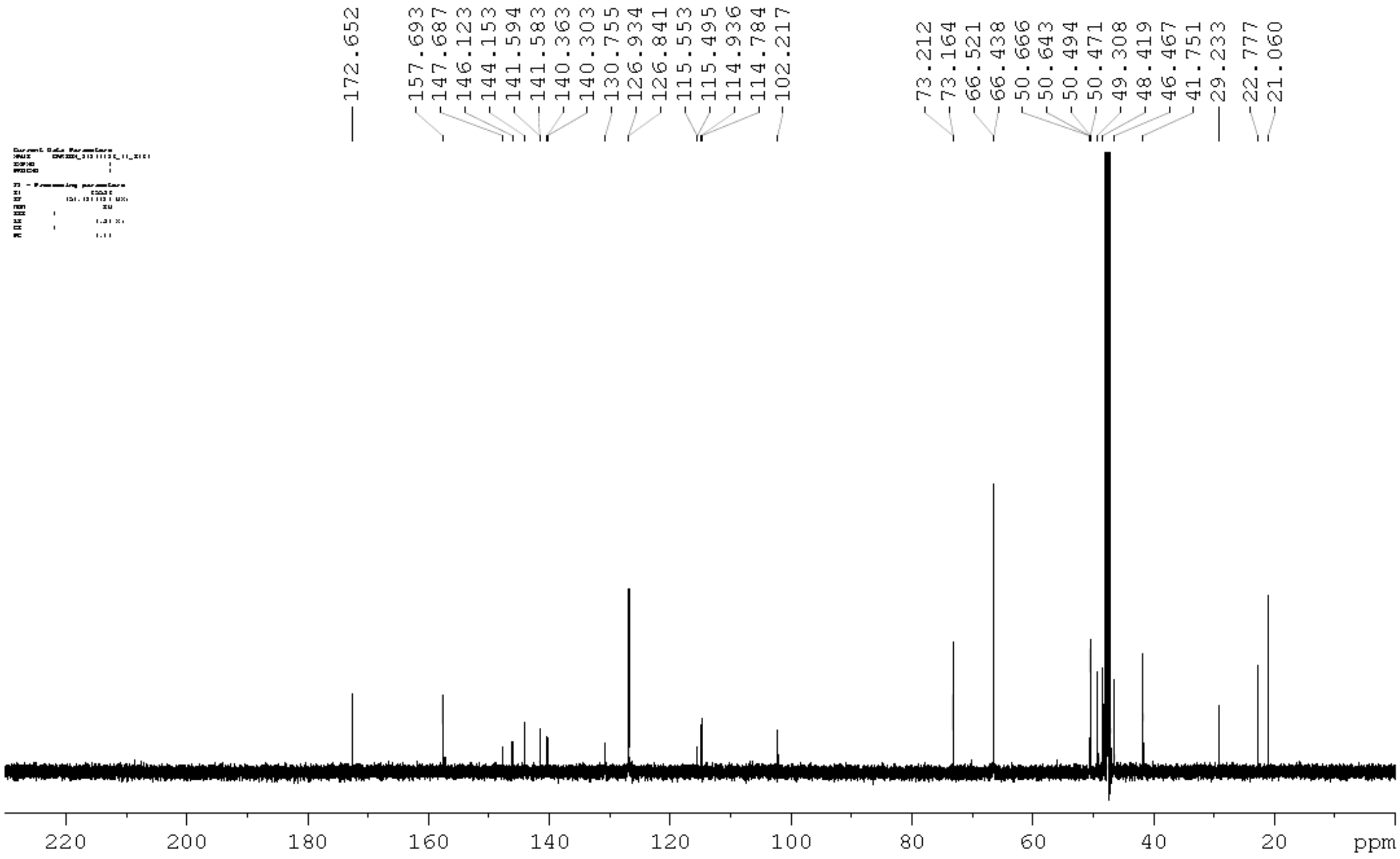

S-II-22. ^{13}C NMR spectrum of compound **11**

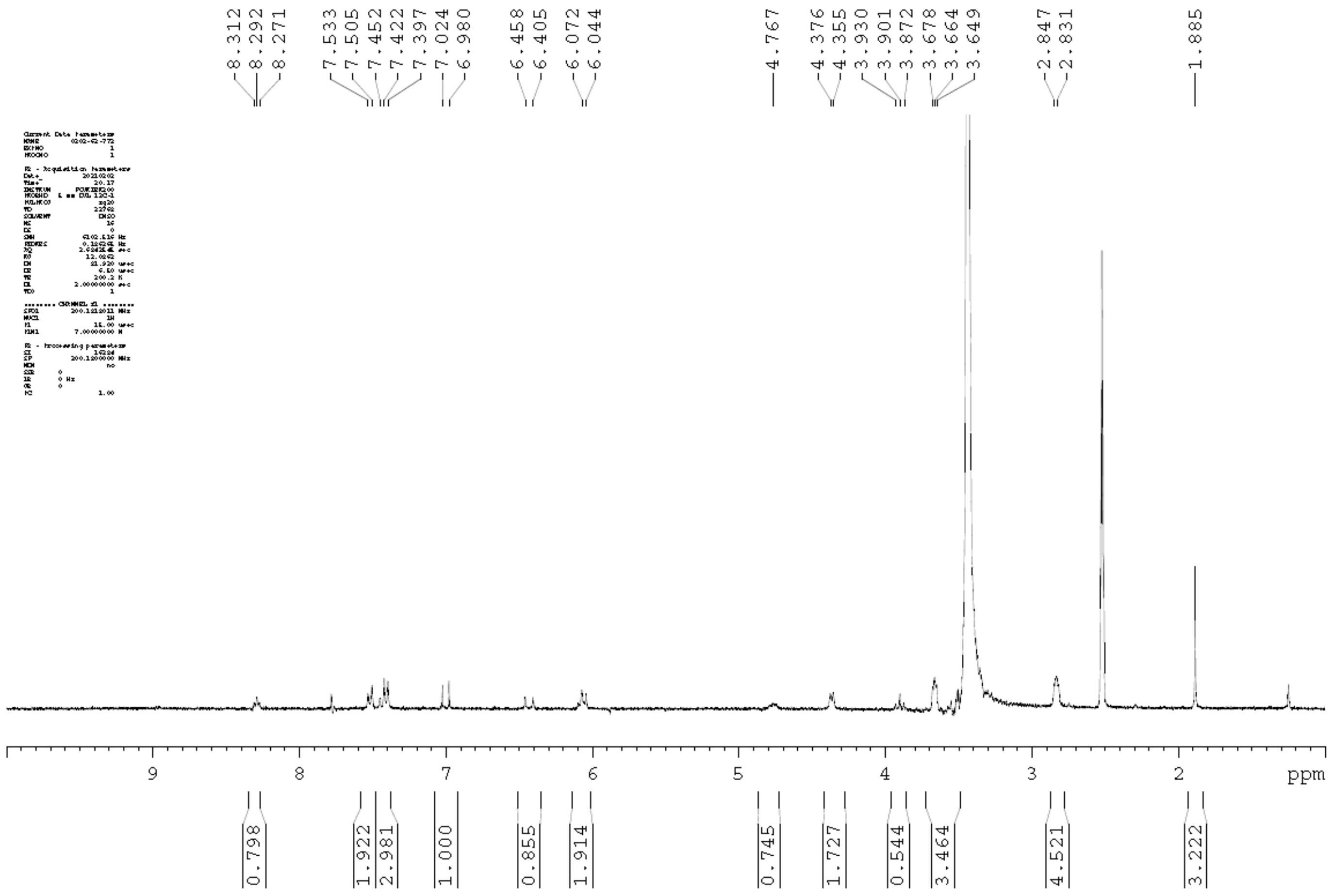

S-II-23. ^{1}H NMR spectrum of compound **12**

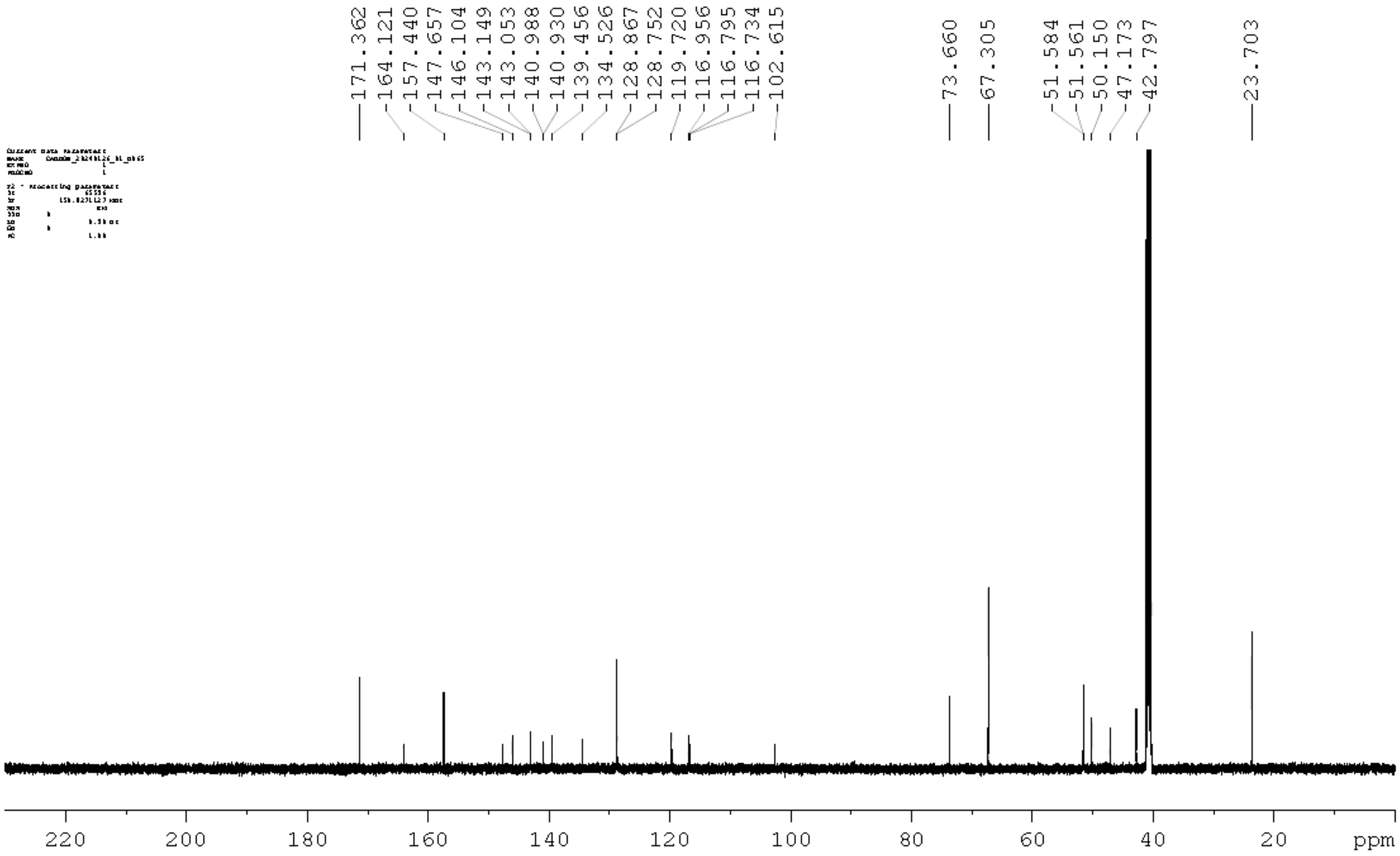

S-II-24. ^{13}C NMR spectrum of compound **12**

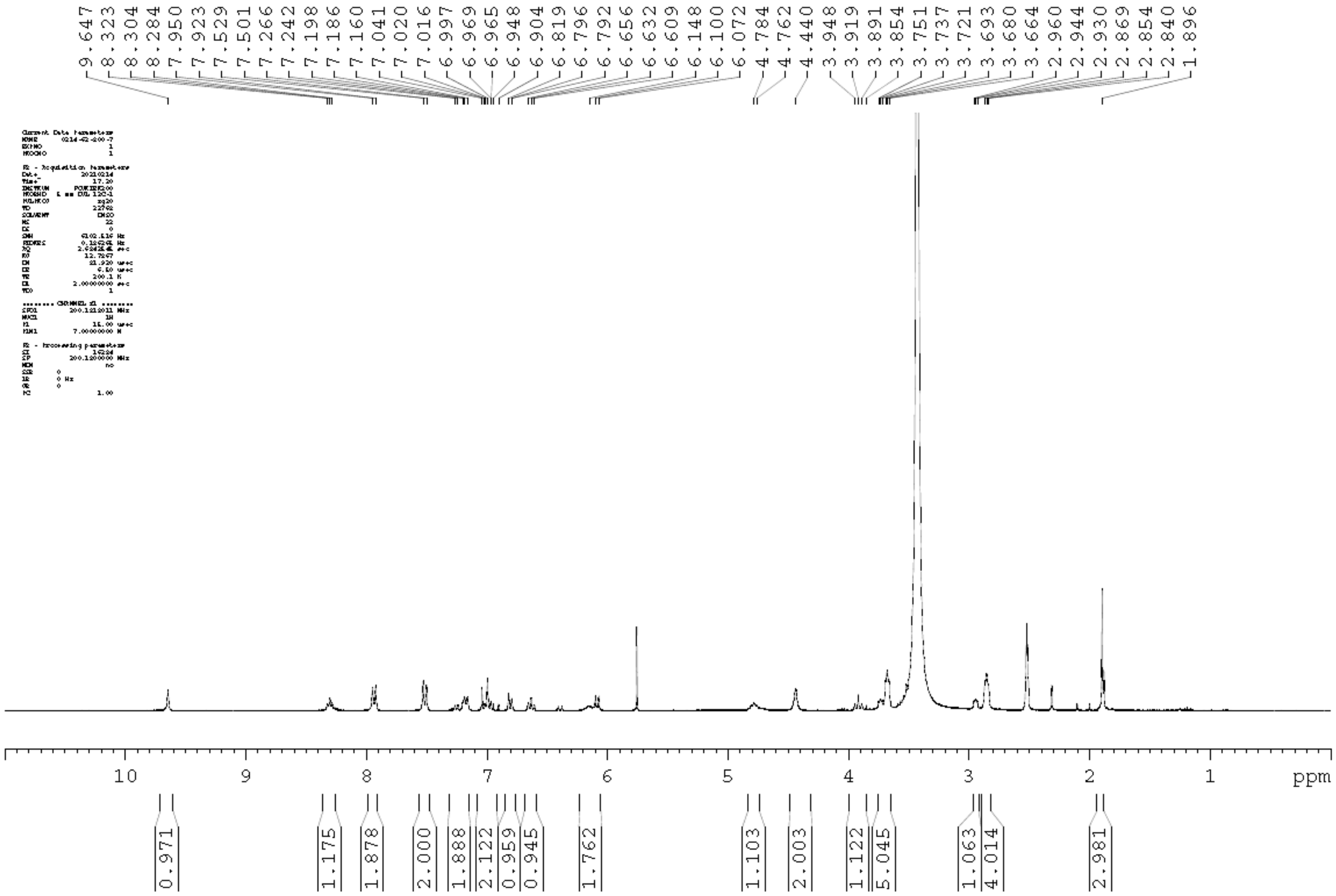

S-II-25. ^{1}H NMR spectrum of compound **13**

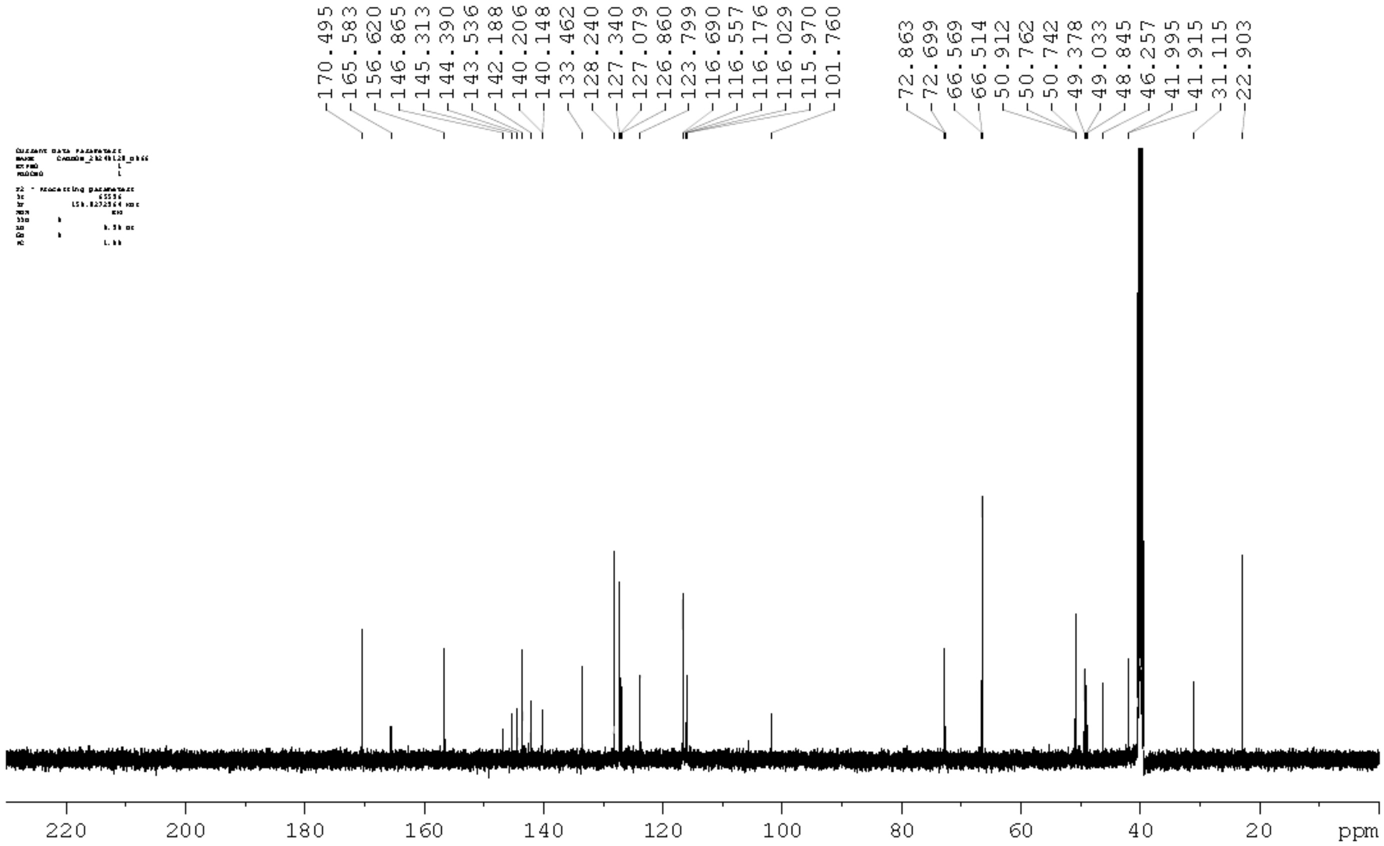

S-II-26. ^{13}C NMR spectrum of compound **13**

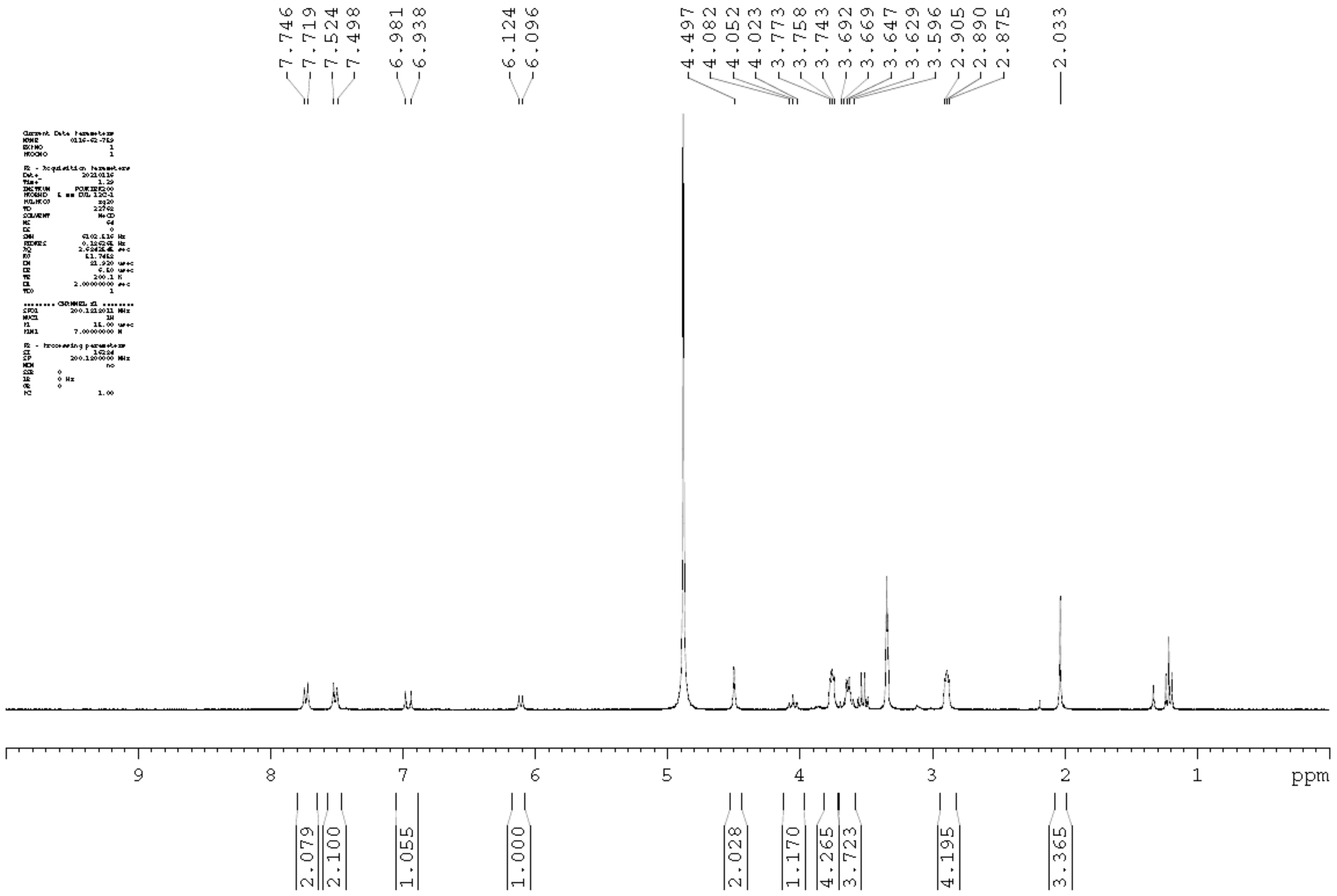

S-II-27. ^{1}H NMR spectrum of compound **14**

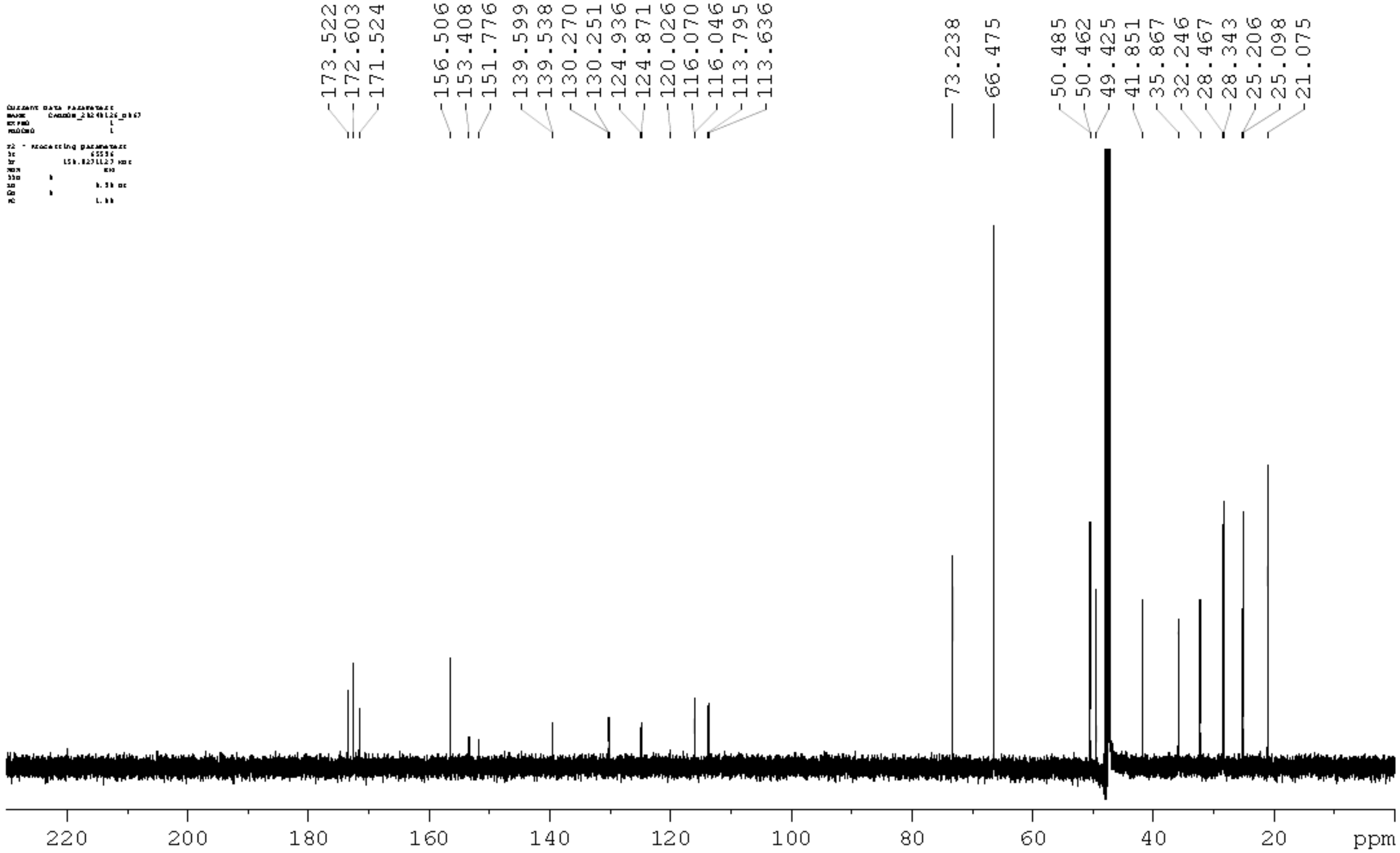

S-II-28. ^{13}C NMR spectrum of compound **14**

Supplementary information III. HPLC purity analysis.

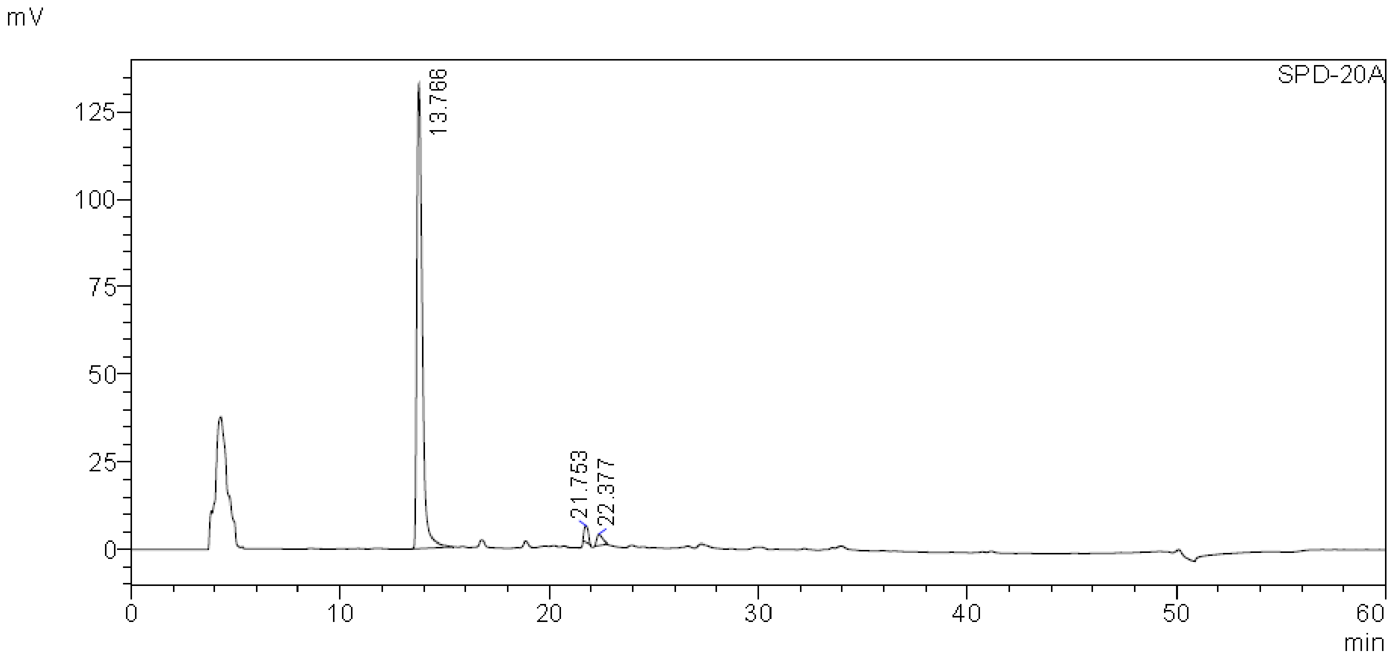

Peak Table

SPD-20A

Peak#	Ret. Time	Area	Height	Conc.	Area%
1	13.766	2403316	132695	95.136	95.136
2	21.753	62368	4701	2.469	2.469
3	22.377	60492	3177	2.395	2.395
Total		2526176	140573		100.000

S-III-1. HPLC purity analysis result of compound **1**

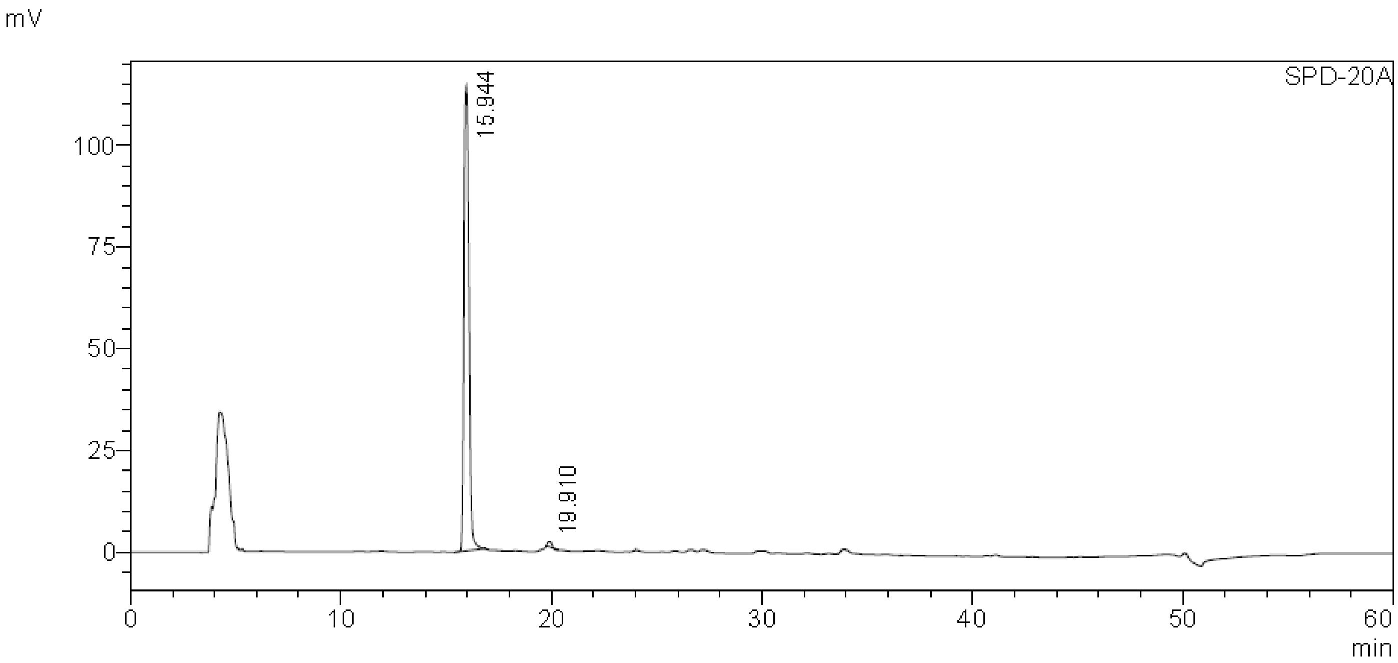

Peak Table

SPD-20A

Peak#	Ret. Time	Area	Height	Conc.	Area%
1	15.944	1948208	114207	99.119	99.119
2	19.910	17322	1280	0.881	0.881
Total		1965529	115487		100.000

S-III-2. HPLC purity analysis result of compound **2**

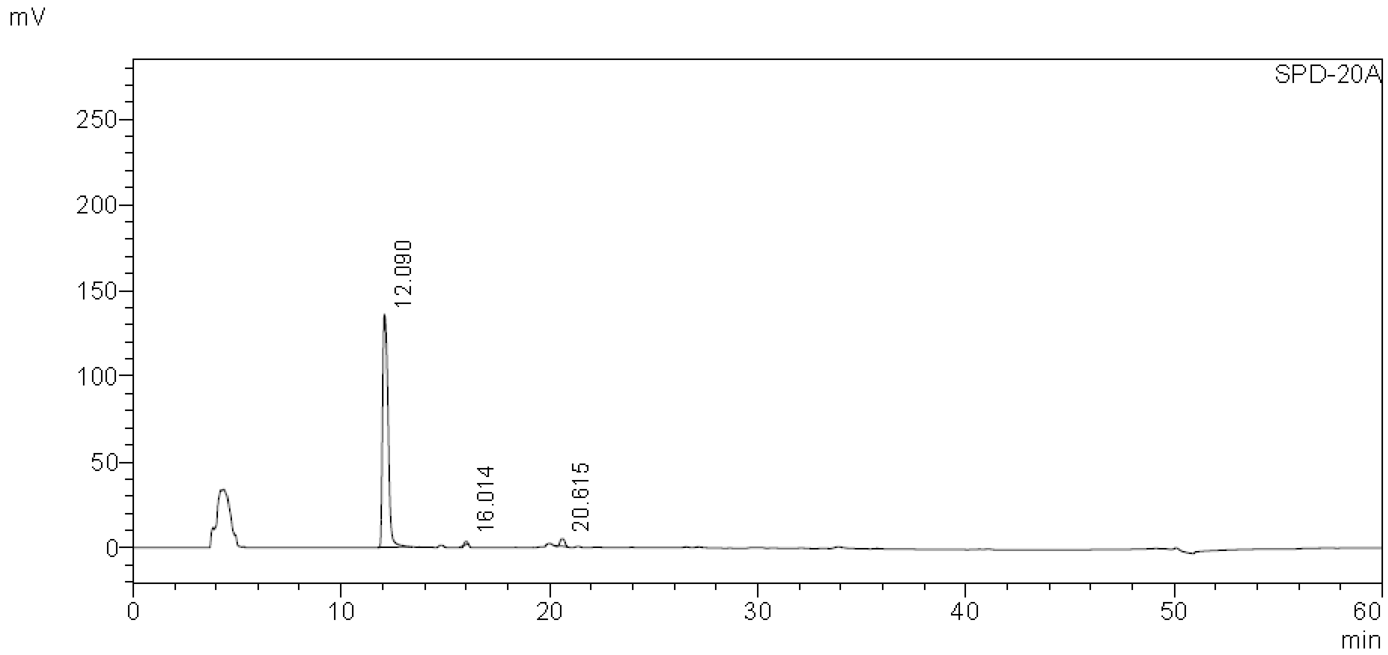

Peak Table

SPD-20A

Peak#	Ret. Time	Area	Height	Conc.	Area%
1	12.090	2312567	135011	96.551	96.551
2	16.014	14320	1759	0.598	0.598
3	20.615	68279	4423	2.851	2.851
Total		2395166	141193		100.000

S-III-3. HPLC purity analysis result of compound **3**

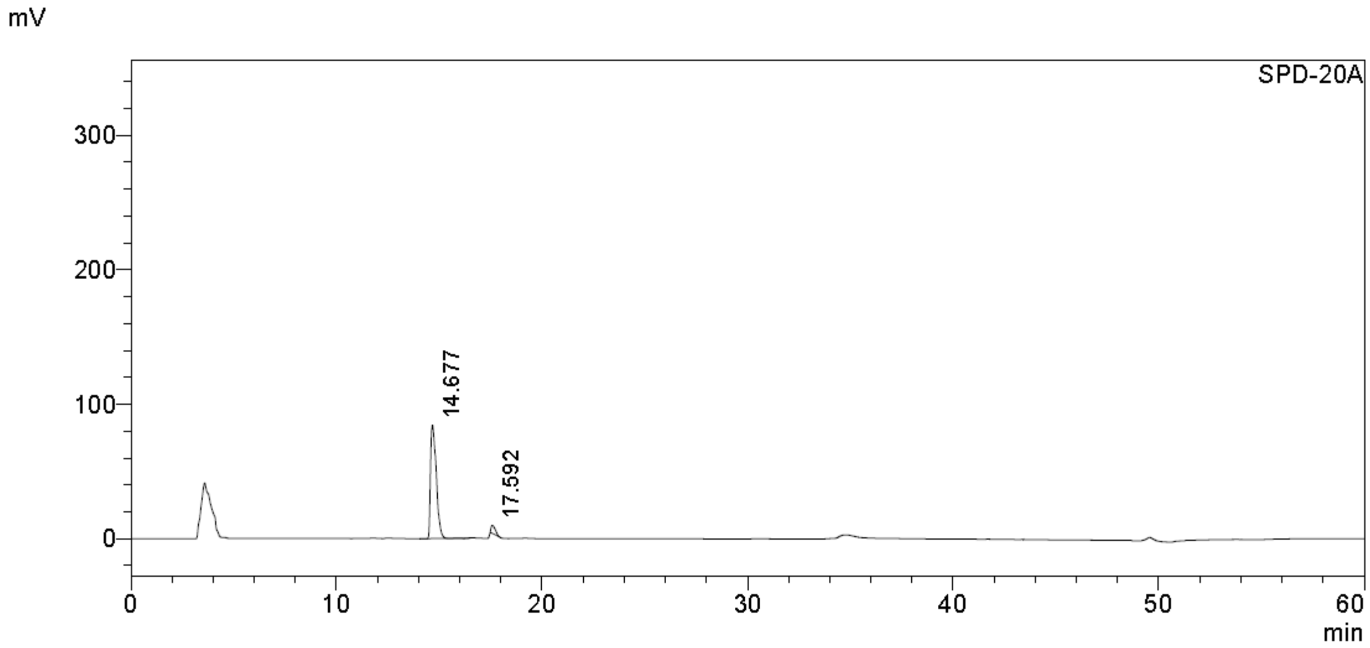

Peak Table

SPD-20A

Peak#	Ret. Time	Area	Height	Conc.	Area%
1	14.677	1624702	84405	95.069	95.069
2	17.592	84278	5969	4.931	4.931
Total		1708980	90374		100.000

S-III-4. HPLC purity analysis result of compound **4**

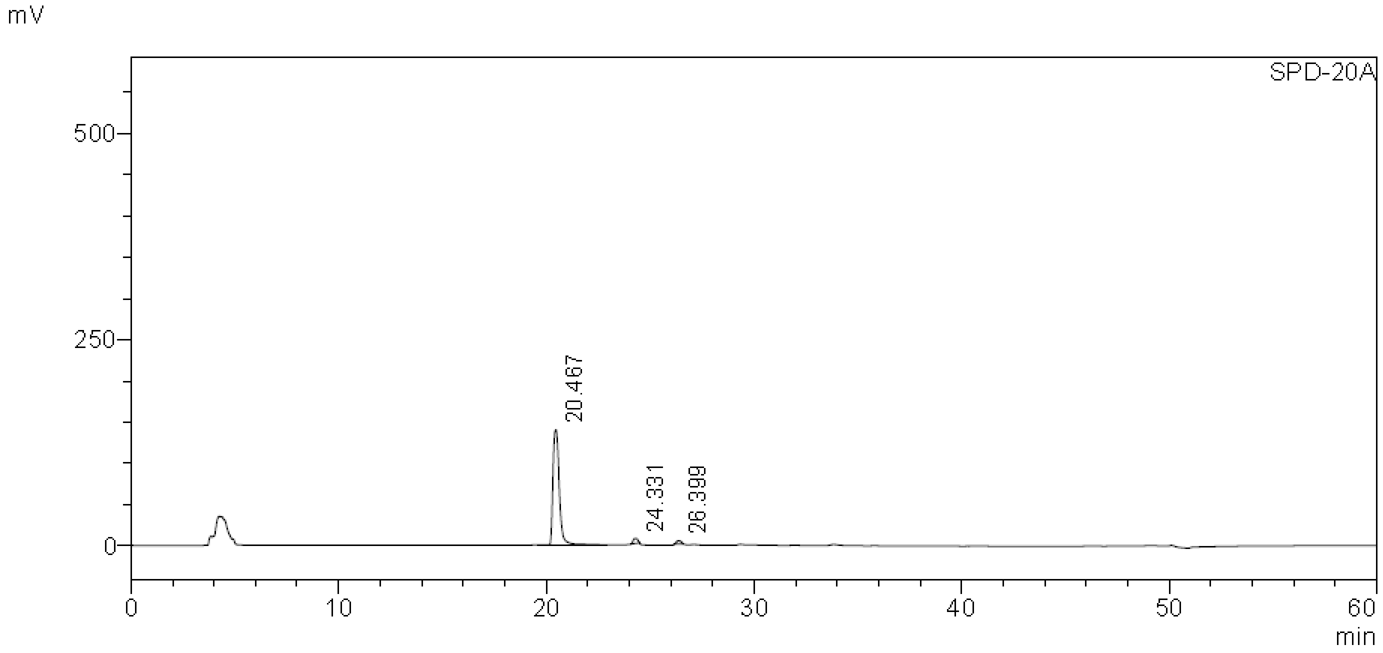

Peak Table

SPD-20A

Peak#	Ret. Time	Area	Height	Conc.	Area%
1	20.467	2919538	140001	95.068	95.068
2	24.331	90247	5943	2.939	2.939
3	26.399	61213	3920	1.993	1.993
Total		3070998	149864		100.000

S-III-5. HPLC purity analysis result of compound **5**

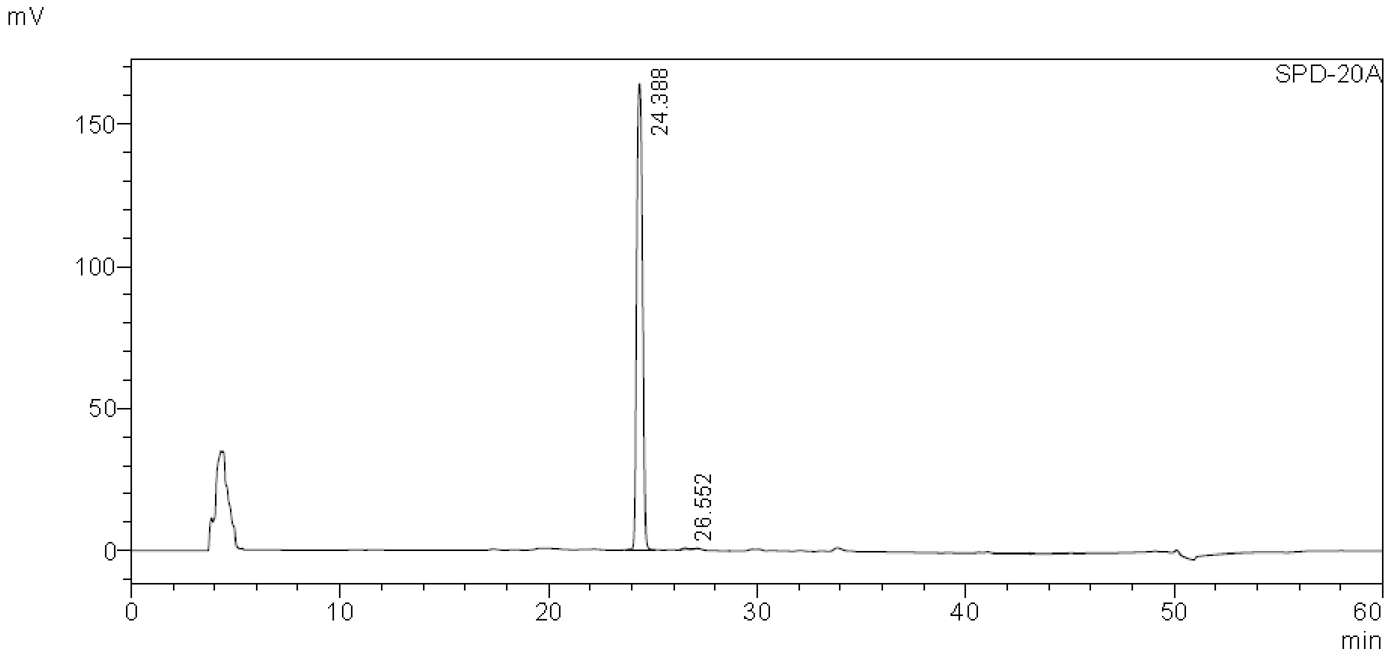

Peak Table

SPD-20A

Peak#	Ret. Time	Area	Height	Conc.	Area%
1	24.388	3134300	163958	99.886	99.886
2	26.552	3593	423	0.114	0.114
Total		3137892	164381		100.000

S-III-6. HPLC purity analysis result of compound **6**

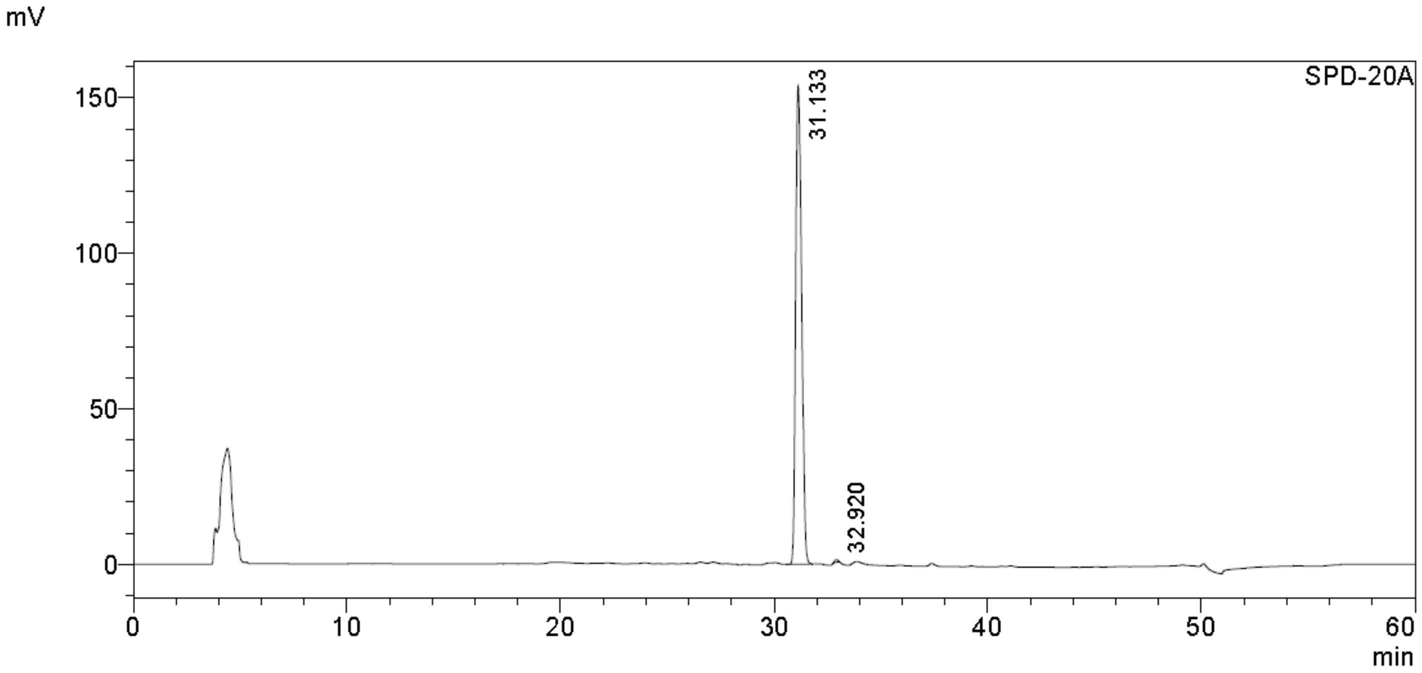

Peak Table

SPD-20A

Peak#	Ret. Time	Area	Height	Conc.	Area%
1	31.133	2978527	153798	99.689	99.689
2	32.920	9287	852	0.311	0.311
Total		2987814	154649		100.000

S-III-7. HPLC purity analysis result of compound **7**

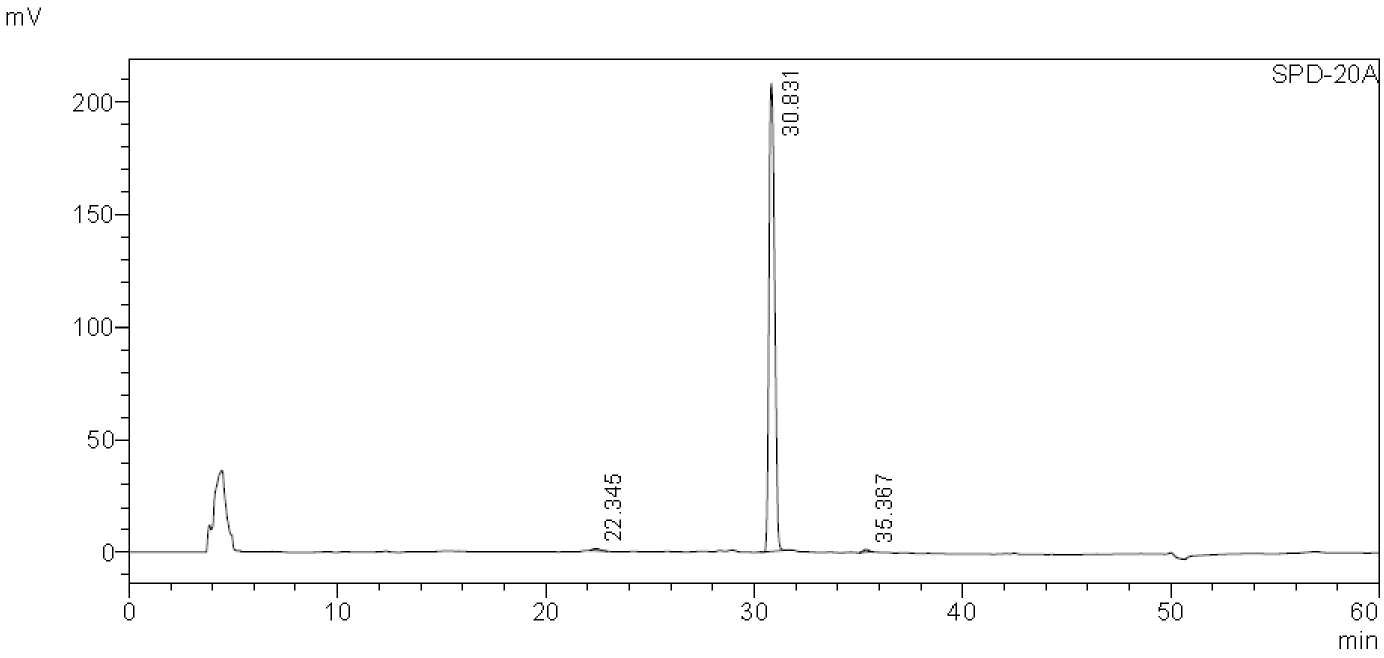

Peak Table

SPD-20A

Peak#	Ret. Time	Area	Height	Conc.	Area%
1	22.345	20533	825	0.513	0.513
2	30.831	3960768	207636	98.975	98.975
3	35.367	20486	1096	0.512	0.512
Total		4001787	209557		100.000

S-III-8. HPLC purity analysis result of compound **8**

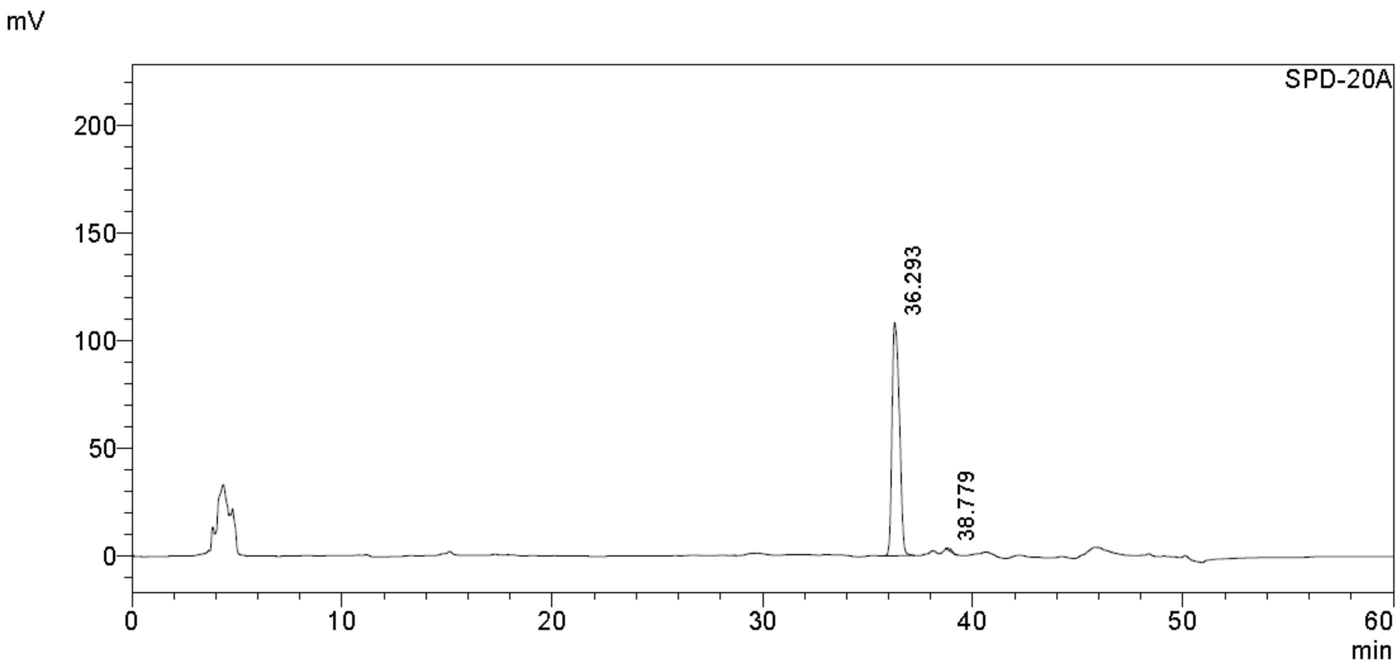

Peak Table

SPD-20A

Peak#	Ret. Time	Area	Height	Conc.	Area%
1	36.293	2651501	108085	99.559	99.559
2	38.779	11737	519	0.441	0.441
Total		2663237	108604		100.000

S-III-9. HPLC purity analysis result of compound **9**

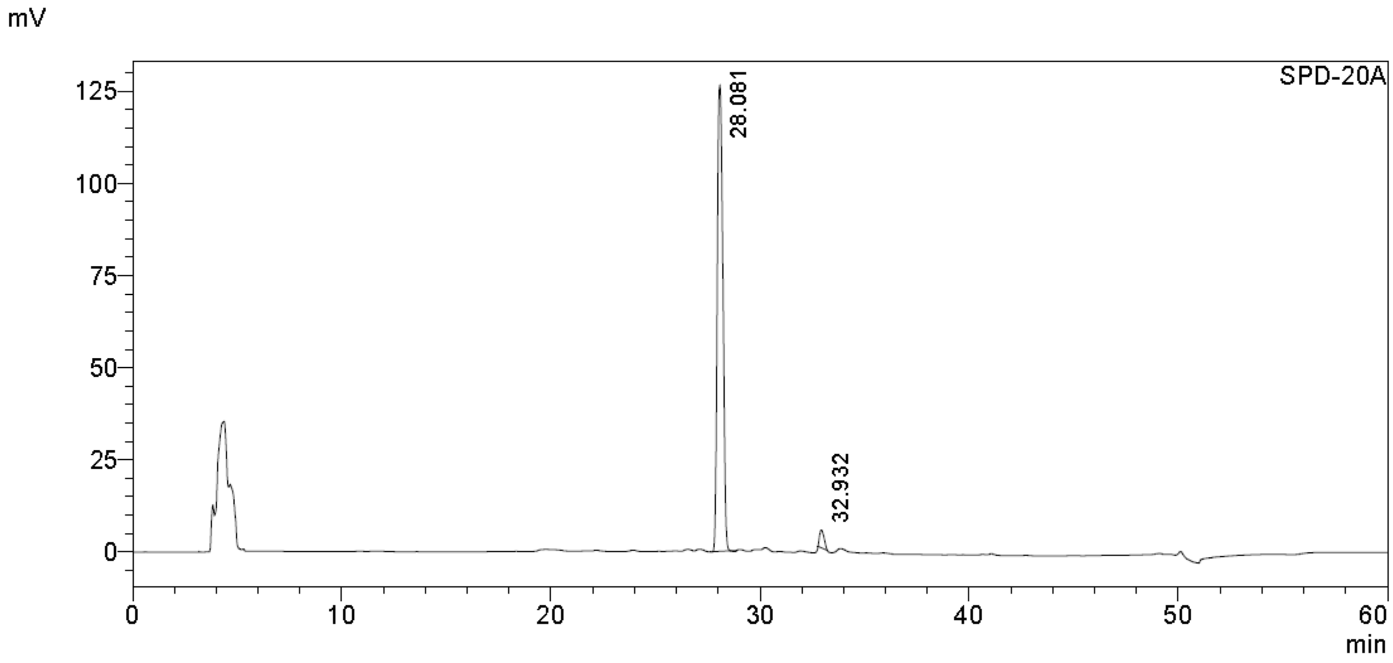

Peak Table

SPD-20A

Peak#	Ret. Time	Area	Height	Conc.	Area%
1	28.081	2365628	126408	96.977	96.977
2	32.932	73750	4891	3.023	3.023
Total		2439378	131299		100.000

S-III-10. HPLC purity analysis result of compound **10**

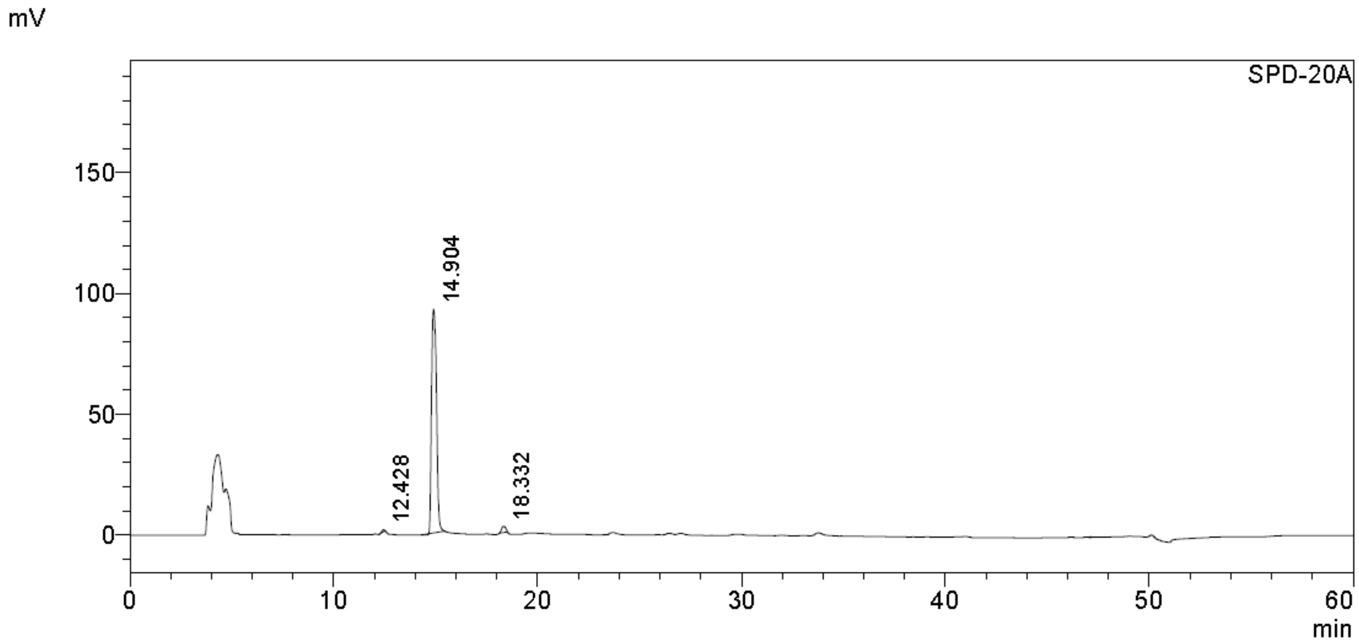

Peak Table

SPD-20A

Peak#	Ret. Time	Area	Height	Conc.	Area%
1	12.428	7669	816	0.486	0.486
2	14.904	1534314	92440	97.188	97.188
3	18.332	36720	2546	2.326	2.326
Total		1578703	95802		100.000

S-III-11. HPLC purity analysis result of compound **11**

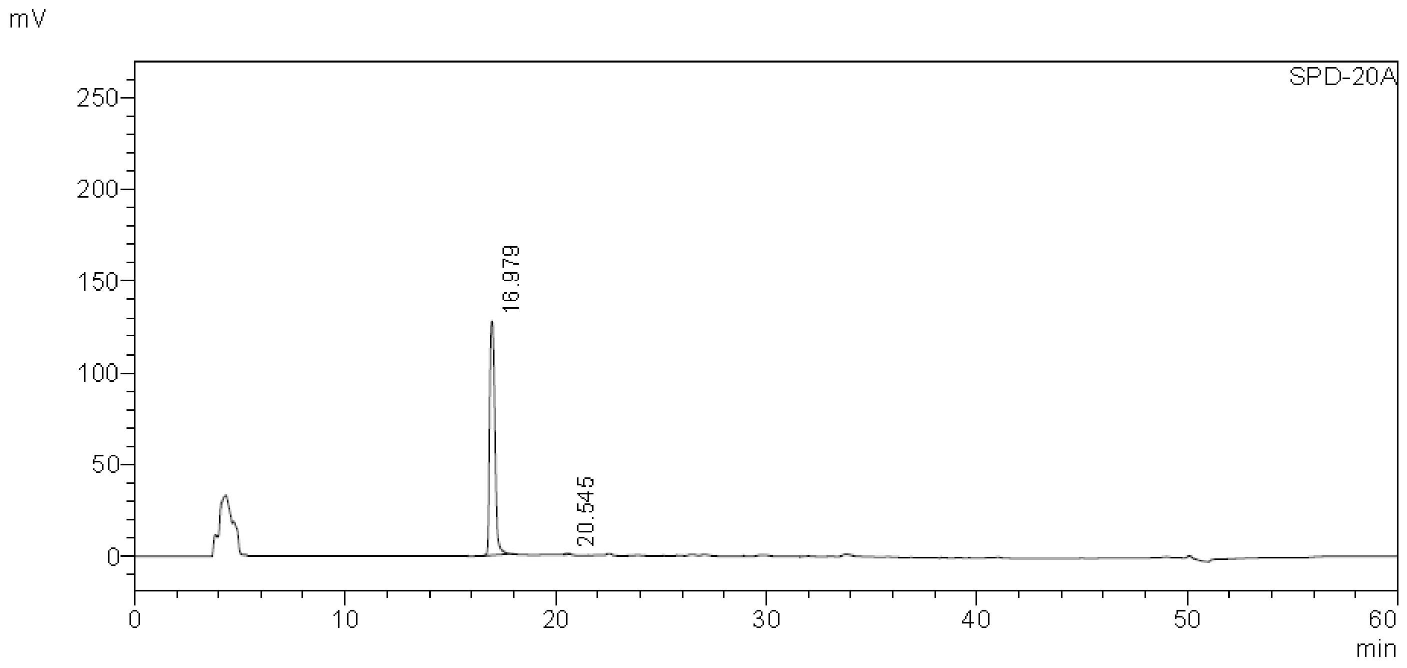

Peak Table

SPD-20A

Peak#	Ret. Time	Area	Height	Conc.	Area%
1	16.979	2265774	127499	99.672	99.672
2	20.545	7467	568	0.328	0.328
Total		2273241	128067		100.000

S-III-12. HPLC purity analysis result of compound **12**

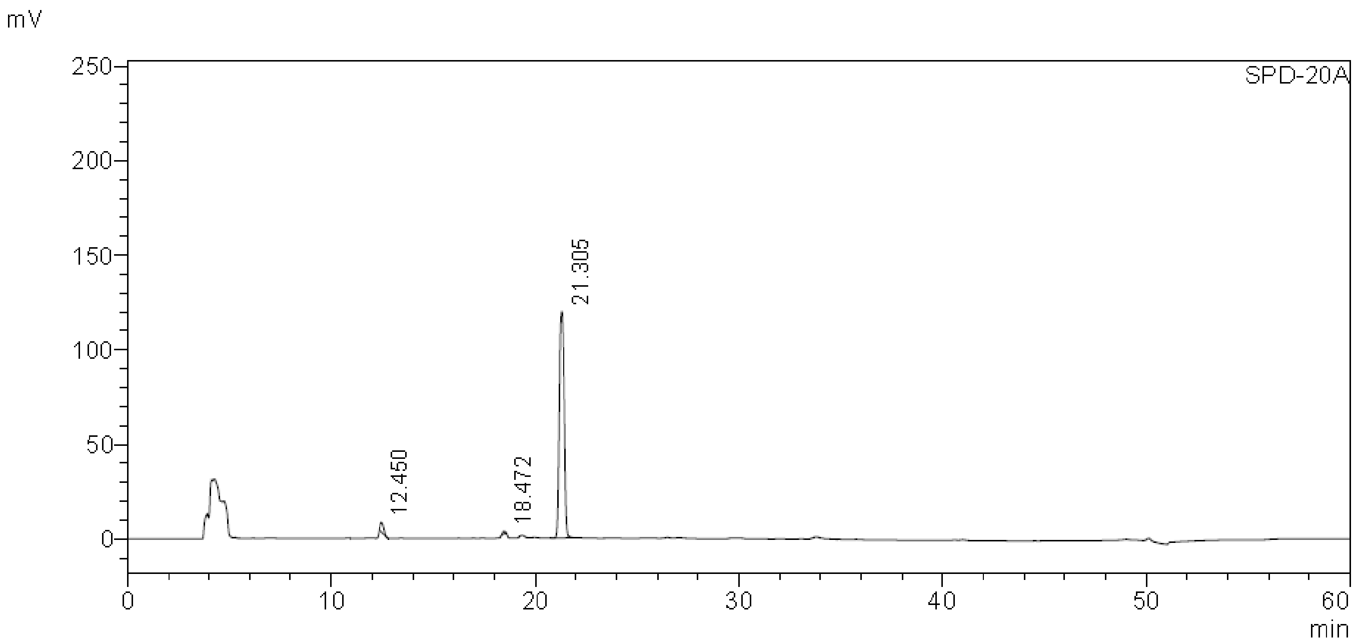

Peak Table

SPD-20A

Peak#	Ret. Time	Area	Height	Conc.	Area%
1	12.450	56723	4852	2.621	2.621
2	18.472	12770	1357	0.590	0.590
3	21.305	2094664	119526	96.789	96.789
Total		2164157	125736		100.000

S-III-13. HPLC purity analysis result of compound **13**

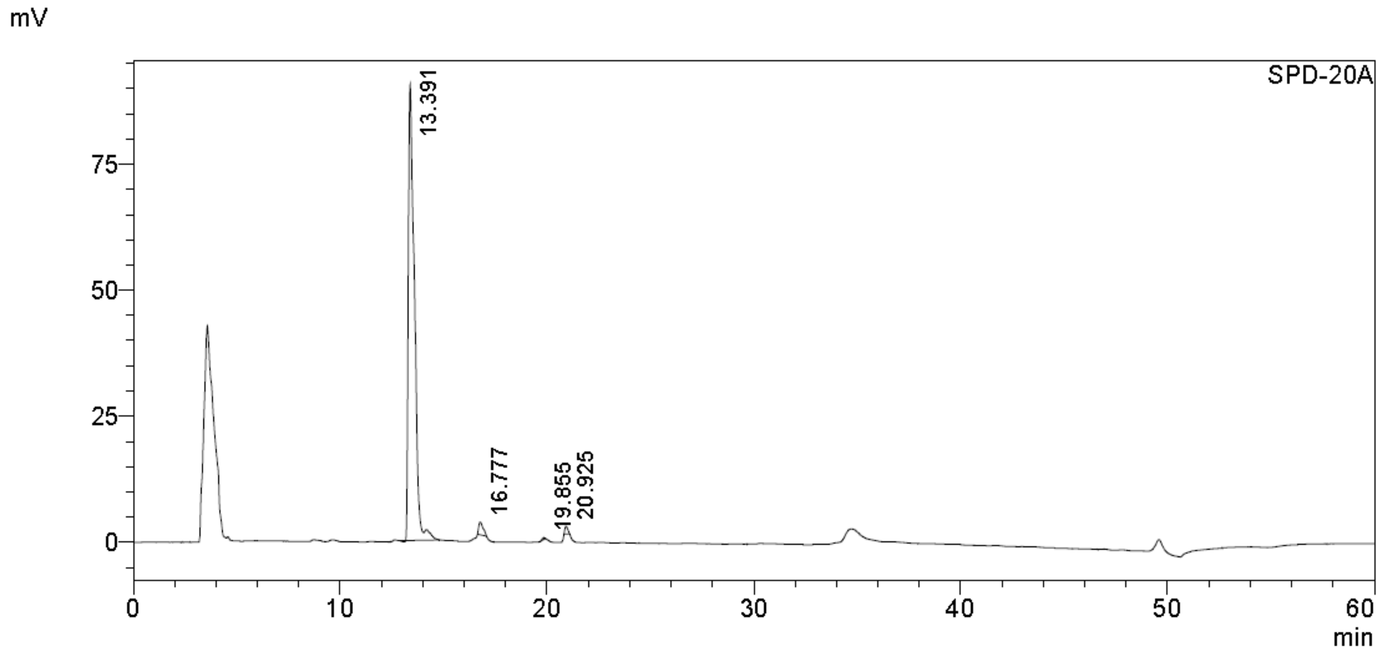

Peak Table

SPD-20A

Peak#	Ret. Time	Area	Height	Conc.	Area%
1	13.391	1841854	90400	97.066	97.066
2	16.777	37432	2537	1.973	1.973
3	19.855	3089	357	0.163	0.163
4	20.925	15150	1551	0.798	0.798
Total		1897525	94846		100.000

S-III-14. HPLC purity analysis result of compound **1**

Supplementary information IV. Inhibitory effects of compound 1-10 on A172R and PT#3R TMZ-resistant cell line.

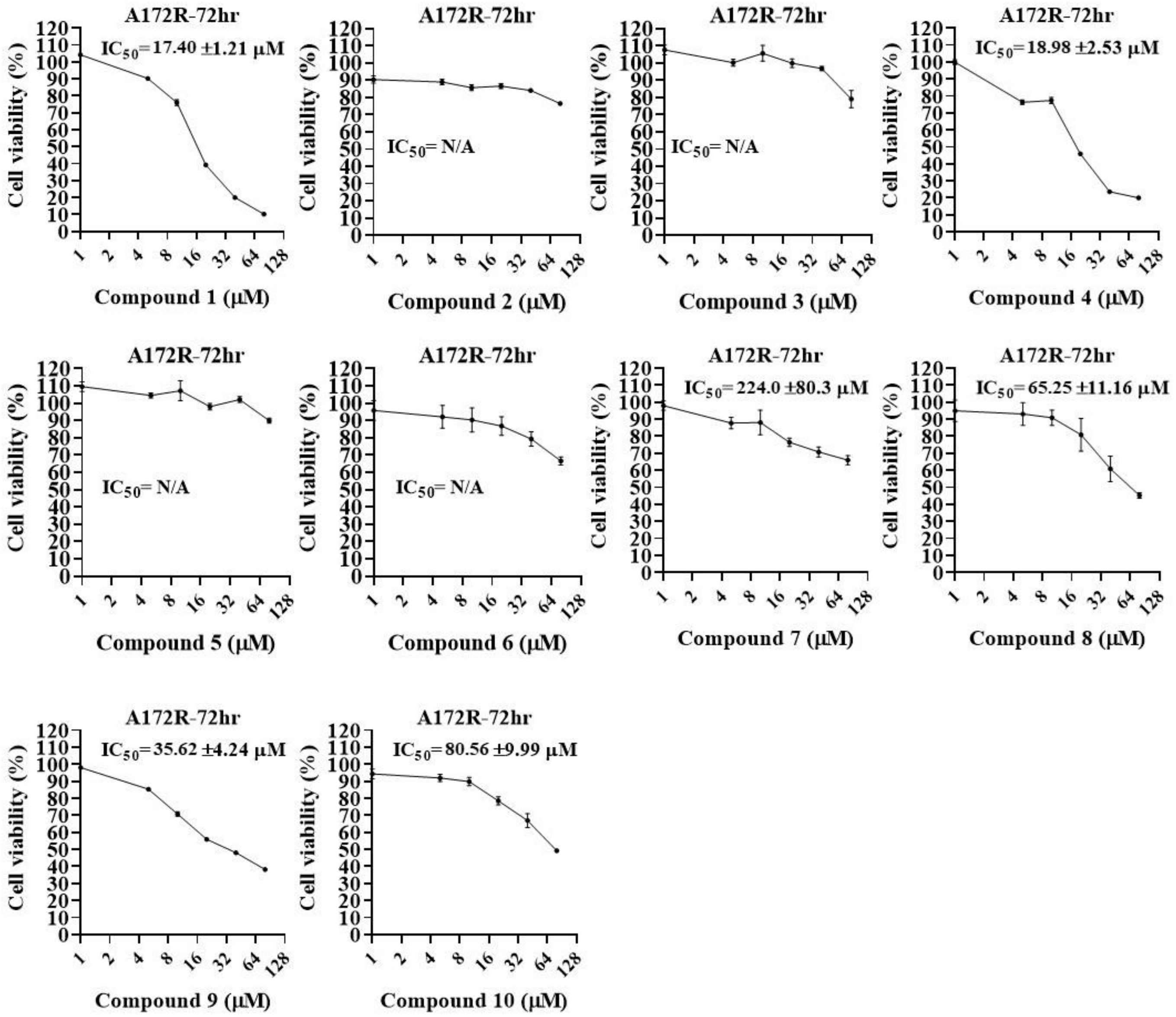

S-IV-1. Inhibitory effects of compound 1-10 on A172R TMZ-resistant GBM cell line were assessed using the MTT assay.

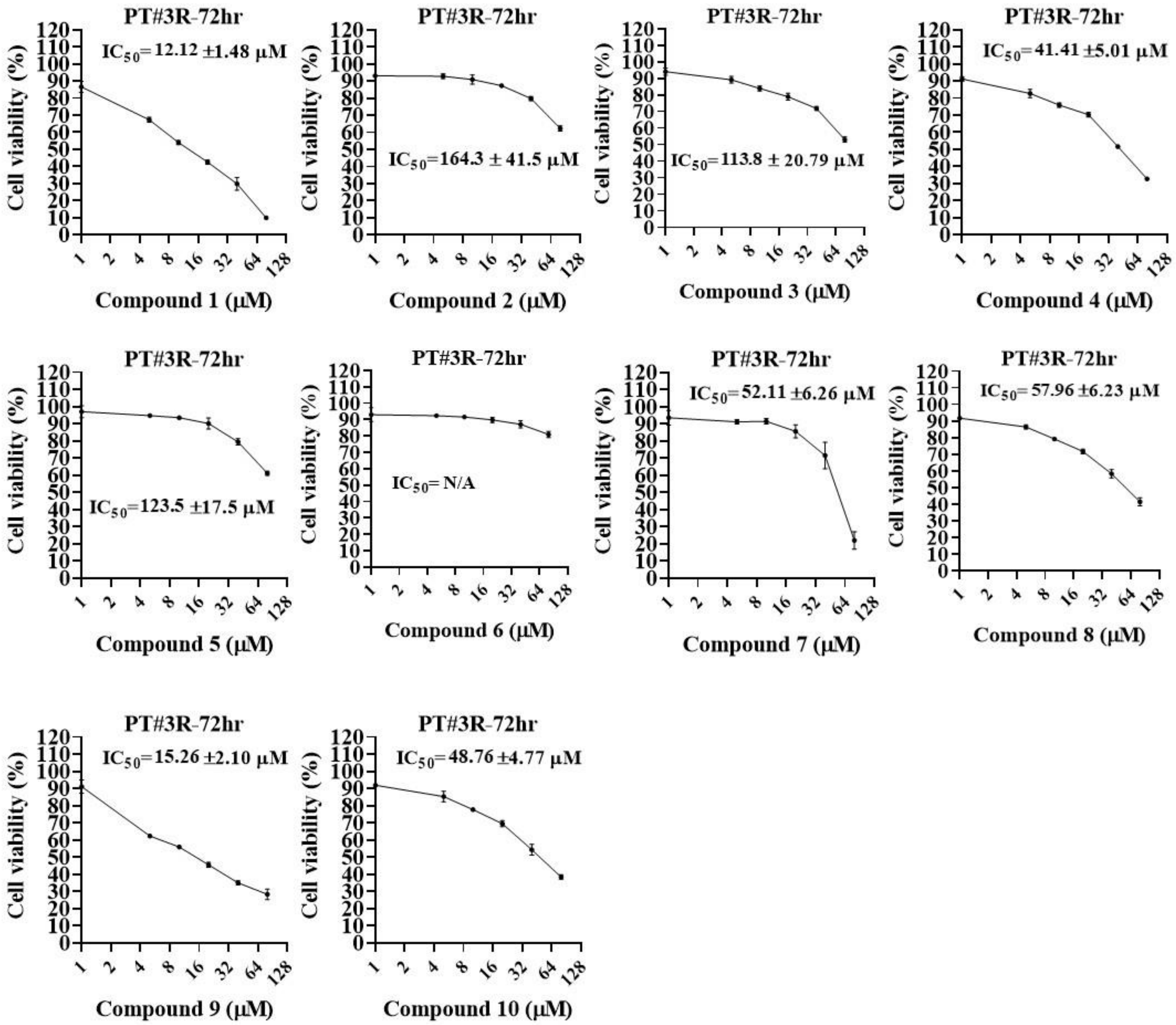

S-IV-2. Inhibitory effects of compound 1-10 on PT#3R TMZ-resistant GBM cell line were assessed using the MTT assay.

Supplementary information V. Schematic diagram of RAD51 amino acid sequence truncation and mutagenesis design.

RAD51-T1

MAMQMQLEANADTSVEEESFGPQPISRLEQCGINANDVKKLEEAGFHTVEAVA
YAPKKELINIKGISEAKADKILTESRSVARLECNSVILVYCTLRLSGSSDSPASASR
VVGTT

RAD51-T2

GGIETGSITEMFGEFRTGKTQICHTLAVTCQLPIDRGGGEGKAMYIDTEGTFRPE
RLLAVAERYGLSGSDVLDNVAYARAFNTDHQTQLL

RAD51-T3

YQASAMMVESRYALLIVDSATALYRTDYSGRGELSARQMHLARFLRMLLRLA
DEFGVAVVITNQVVAQVDGAAMFAADPKKPIGGNIIAHASTTRLYLRKGRGETR
ICKIYDSPCLPEAEAMFAINADGVGDAKD

Helix-hairpin-helix domain

ATPase motifs domain Walker A

ATPase motifs domain Walker B

Nuclear export signal; masked by the interaction with BRCA2

ATP binding site [chemical binding site]

S-V-1. Schematic diagram of RAD51 amino acid sequence truncation and design.

RAD51 Amino Acid sequence (T3-WT):

YKSGLRSRAYQASAMMVESRYALLIVDSATALYRTDYSGRGELSARQMHLARFLRMLLRLADEF
GVAVVITNQVVAQVDGAAMFAADPKKPIGGNIIAHASTTRLYLRKGRGETRICKIYDSPCLPEAEA
MFAINADGVGDAKD-PPGSTGSR-LI

Amino Acid sequence (T3-Mut1-K285286A) :

YKSGLRSRAYQASAMMVESRYALLIVDSATALYRTDYSGRGELSARQMHLARFLRMLLRLADEF
GVAVVITNQVVAQVDGAAMFAADPAAPIGGNIIAHASTTRLYLRKGRGETRICKIYDSPCLPEAEA
MFAINADGVGDAKD-PPGSTGSR-LI

Amino Acid sequence (T3-Mut2-K305A) :

YKSGLRSRAYQASAMMVESRYALLIVDSATALYRTDYSGRGELSARQMHLARFLRMLLRLADEF
GVAVVITNQVVAQVDGAAMFAADPKKPIGGNIIAHASTTRLYLRAGRGETRICKIYDSPCLPEAEA
MFAINADGVGDAKD-PPGSTGSR-LI

Amino Acid sequence (T3-Mut3-K314A) :

YKSGLRSRAYQASAMMVESRYALLIVDSATALYRTDYSGRGELSARQMHLARFLRMLLRLADEF
GVAVVITNQVVAQVDGAAMFAADPKKPIGGNIIAHASTTRLYLRKGRGETRICAIYDSPCLPEAEA
MFAINADGVGDAKD-PPGSTGSR-LI

Amino Acid sequence (T3-Mut4-K339A):

YKSGLRSRAYQASAMMVESRYALLIVDSATALYRTDYSGRGELSARQMHLARFLRMLLRLADEF
GVAVVITNQVVAQVDGAAMFAADPKKPIGGNIIAHASTTRLYLRKGRGETRICKIYDSPCLPEAEA
MFAINADGVGDAAD-PPGSTGSR-LI

S-V-2. Schematic diagram of RAD51 amino acid sequence mutagenesis design.

Supplementary information VI. Computational AI BBB penetration prediction.

Machine learning model predictions indicate that structural modifications successfully preserve the ability to penetrate the blood-brain barrier while enhancing the potency of compound 1 in treating GBM. A dataset comprising over 2,000 compounds was used to train a machine learning model for predicting blood-brain barrier permeability, achieving an overall accuracy of 0.92 on the test dataset. This model was then applied to evaluate the blood-brain barrier penetration potential of both linezolid and compound 1. Although compound 1 demonstrated a slightly lower score compared to linezolid, it achieved a confidence score of 0.6921, exceeding the threshold of 0.5. These findings confirm that the structural modifications of linezolid retained its ability to cross the blood-brain barrier.

Confidence score	Non-penetrate	Penetrate
**Linezolid**	0.1994	0.8006
**compound 1**	0.3079	0.6921

S-VI-Prediction of blood-brain barrier penetration of Linezolid and compound 1 (Compound **1**) by the K-nearest neighbors (KNN) algorithm.

Supplementary information VII. Bioanalysis method validation.

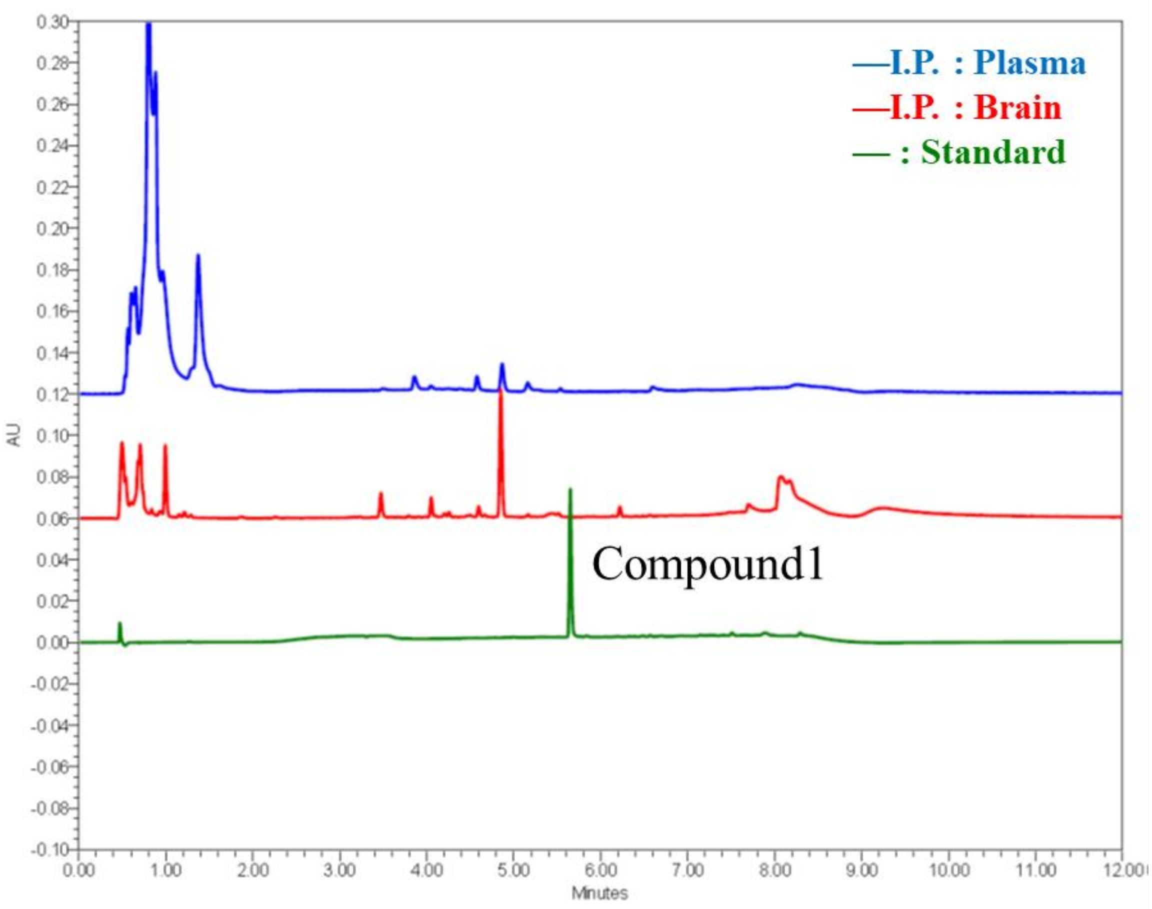

S-VII-1. Representative chromatogram of compound **1** and blank samples (specificity against different matrices)

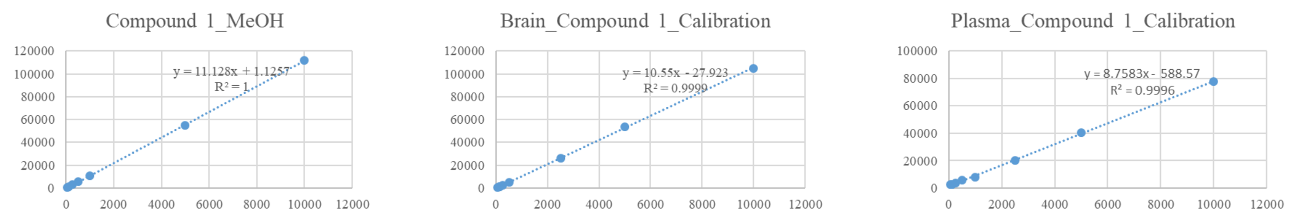

Matrix	Equation	R^2 (linearity)	Matrix Effect (%)
Standard	y = 11.128x + 1.1257	0.9999	-
Brain	y = 10.55x - 27.923	0.9999	-5.5
Plasma	y = 8.7583x - 588.57	0.9995	-21.3

S-VII-2. Standard curves, correlation coefficients in various of matrices all three curves ranging from 125-10000 ng/mL

Sample	QC conc. (ng/mL)	Intra-day (n=6)		Inter-day (n=3)	
		Precision(RSD, %)	Accuracy (mean, %)	Precision(RSD, %)	Accuracy (mean, %)
Brain	125	14.11	-0.47	8.30	12.68
	500	9.36	-4.89	6.14	-6.46
	1000	4.58	-3.14	0.07	-2.59
	10000	3.72	-2.84	4.54	-1.02

Sample	QC conc. (ng/mL)	Intra-day (n=6)		Inter-day (n=3)	
		Precision(RSD, %)	Accuracy (mean, %)	Precision(RSD, %)	Accuracy (mean, %)
Plasma	125	19.29	-6.21	17.67	-9.37
	500	7.99	-6.67	5.51	-5.23
	1000	7.52	-3.89	4.43	7.39
	10000	2.74	7.85	4.54	12.59

S-VII-3. Precision, Accuracy of compound **1** in brain tissue and plasma sample of mice.

Matrix	Compound **1** added (ng/mL)	Recovery (n=3), % (RSD)
Brain	5000	90.06 (12.68)
	10000	64.05 (11.93)

Matrix	Compound **1** added (ng/mL)	Recovery (n=3), % (RSD)
Plasma	5000	87.17 (3.93)
	10000	69.57 (3.77)

S-VII-4. Extraction recovery of compound **1** in brain tissue and plasma of mice.

Supplementary information VIII. Body weight changes in mice administered with vehicle, TMZ, and Compound 1 *in vivo*.

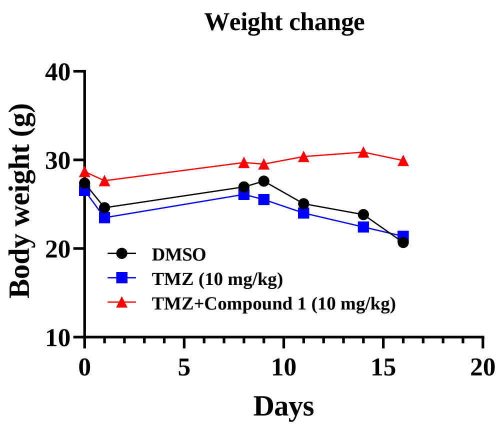

S-VIII-1. Body weight changes in mice administered with DMSO, TMZ, and Compound **1** intraperitoneally following orthotopic CT-2A tumor implantation.

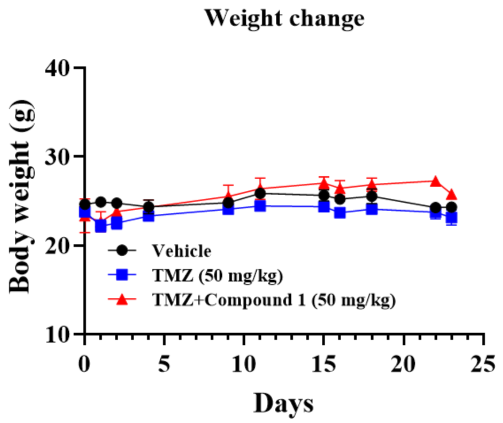

S-VIII-2. Body weight changes in mice administered with vehicle, TMZ, and Compound **1** intraperitoneally following orthotopic CT-2AR tumor implantation.

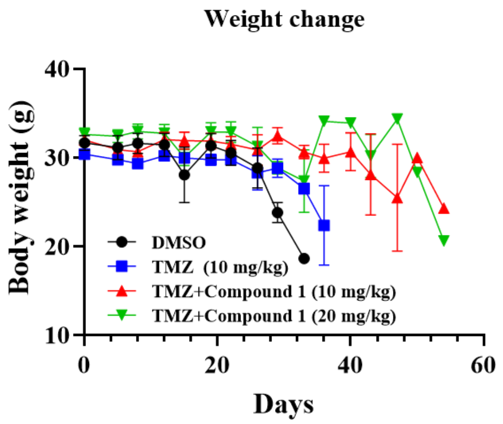

S-VIII-3. Body weight changes in mice administered with vehicle, TMZ, and Compound **1** intraperitoneally following orthotopic PT#3-R tumor implantation.

Supplementary information IX. Supporting Reference

1. Ostermann, S.; Csajka, C.; Buclin, T.; Leyvraz, S.; Lejeune, F.; Decosterd, L. A.; Stupp, R., Plasma and cerebrospinal fluid population pharmacokinetics of temozolomide in malignant glioma patients. *Clin Cancer Res* **2004,** *10* (11), 3728-36.

2. Sabbatani, S.; Manfredi, R.; Frank, G.; Chiodo, F., Linezolid in the treatment of severe central nervous system infections resistant to recommended antimicrobial compounds. *Infez Med* **2005,** *13* (2), 112-9.

3. Hanson, J. E.; La, H.; Plise, E.; Chen, Y. H.; Ding, X.; Hanania, T.; Sabath, E. V.; Alexandrov, V.; Brunner, D.; Leahy, E.; Steiner, P.; Liu, L.; Scearce-Levie, K.; Zhou, Q., SAHA enhances synaptic function and plasticity in vitro but has limited brain availability in vivo and does not impact cognition. *PLoS One* **2013,** *8* (7), e69964.

4. Zhang, W.; Oh, J. H.; Zhang, W.; Rathi, S.; Larson, J. D.; Wechsler-Reya, R. J.; Sirianni, R. W.; Elmquist, W. F., Central Nervous System Distribution of Panobinostat in Preclinical Models to Guide Dosing for Pediatric Brain Tumors. *J Pharmacol Exp Ther* **2023,** *387* (3), 315-327.
